# Structure Reassignment of Laurefurenynes A and B by Computation and Total Synthesis

**DOI:** 10.1002/chem.201302349

**Published:** 2013-08-21

**Authors:** David J Shepherd, Phillip A Broadwith, Bryony S Dyson, Robert S Paton, Jonathan W Burton

**Affiliations:** [a] Department of Chemistry, University of Oxford, Chemistry Research Laboratory, Mansfield RoadOxford, OX1 3TA (UK)

**Keywords:** density functional calculations, natural products, NMR spectroscopy, structure determination, total synthesis

**Figure 1:**
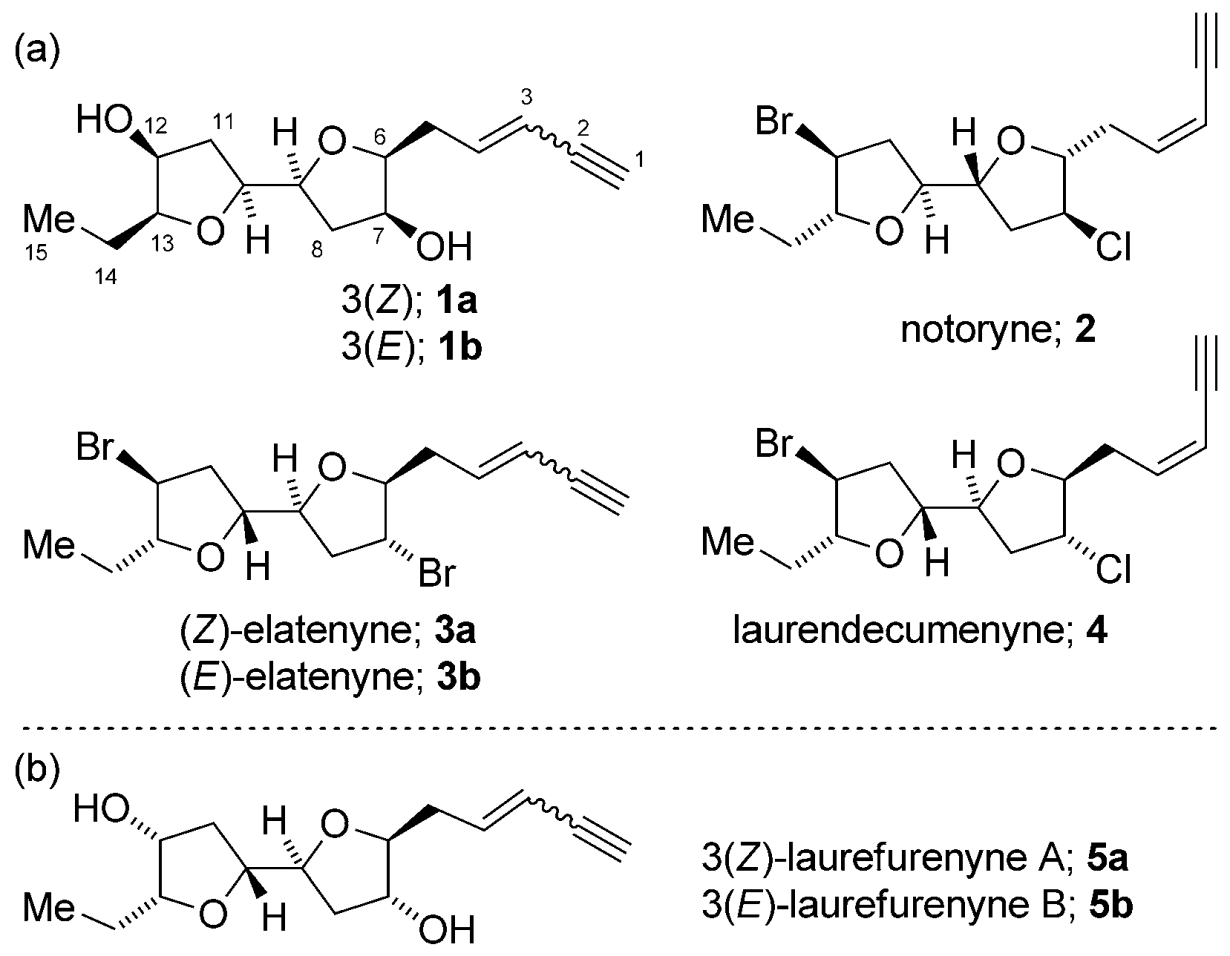
a) Reported structures of laurefurenynes A and B 1, and structures of other 2,2′-bifuranyl natural products. b) Reassigned stereostructures of laurefurenynes A and B (5).

In 2010, the structures of six new cyclic ethers isolated from *Laurencia* spp. were reported and named the laurefurenynes.^1^ Laurefurenynes A and B were assigned the 2,2′-bifuranyl structures **1** on the basis of extensive 1D and 2D NMR experiments with the relative configuration being assigned on the basis of ^1^H NMR NOESY experiments in conjunction with molecular modelling (Figure 1 a). Laurefurenynes A and B are structurally related to a number of other 2,2′-bifuranyl natural products from *Laurencia* spp. including notoryne **2**,^2^ (*Z*)-3, 4 and (*E*)-elatenyne **3**,^5^ and laurendecumenyne **4**.^4^ The structure of elatenyne was originally assigned as a pyrano[3,2-*b*]pyran.^3^ We have previously reassigned the originally proposed structure of elatenyne on the basis of DFT calculations of NMR chemical shifts,^6^ biosynthetic postulates and total synthesis.^7^–^10^ Herein, we report the reassignment of the stereostructures of laurefurenynes A and B as **5** on the basis of a ^13^C NMR chemical shift/relative configuration correlation coupled with DP4 analysis (Figure 1 b).^11^, ^12^ Additionally, confirmation of the reassigned stereostructure of laurefurenyne B **5 b** is reported on the basis of total synthesis both by us and by the Britton research group.^13^ The reassigned structures of laurefurenynes A and B fit with our recently proposed biogenesis of elatenyne.^8^, ^10^ This work further demonstrates the power of this combined computational/synthetic approach for the structure determination of natural products and small highly flexible organic molecules.

**Scheme 1:**
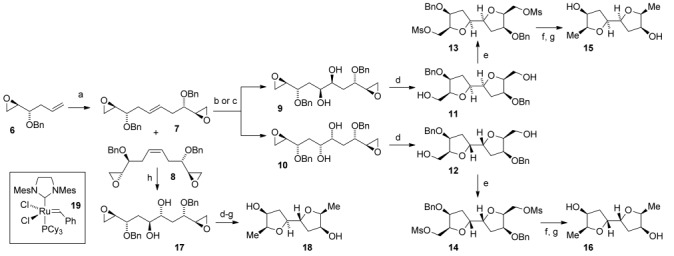
Synthesis of model 2,2′-bifuranyls. a) Catalyst 19 (1 mol %), CH_3_CO_2_H (10 mol %), CH_2_Cl_2_, reflux, 3:1 partially separable mixture of 7/8, 67 %; b) (DHQ)_2_PHAL, K_3_FeCN_6_, K_2_OsO_4_⋅2 H_2_O, K_2_CO_3_, CH_3_SO_2_NH_2_, *t*BuOH, water, 0 °C, 3.5:1 mixture of 9/10 with cyclized 11 and 12, 97 %; c) (DHQD)_2_PHAL, K_3_FeCN_6_, K_2_OsO_4_⋅2 H_2_O, K_2_CO_3_, CH_2_SO_3_NH_2_, *t*BuOH, water, 0 °C, 1:7 mixture of 9/10, with cyclized 11 and 12 (quant.); d) Amberlyst-15, CDCl_3_, RT; e) MsCl, Et_3_N, CH_2_Cl_2_, 0 °C→RT, 13 (72 %), 14 (20 %) from 3.5:1 mixture of 9/10; 13 (12 %), 14 (83 %) from 1:7 mixture of 9/10; f) (CH_3_CH_2_)_3_BHLi, THF, 0 °C→RT; g) H_2_, Pd/C, EtOH, RT, 15 (80 %) from 13, 16 (60 %) from 14, 18 (27 %) from 8; h) K_2_OsO_4_⋅2 H_2_O, 4-methylmorpholine *N*-oxide, acetone, water, 0 °C→RT, 97 %. Bn=benzyl, Ms=methanesulfonyl.

**Figure 2:**
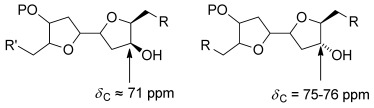
Chemical shift stereochemistry correlation; R=CH_3_ or CH=CH_2_, R′=CH_3_ or CH=CH_2_, P=H, benzyl, 4-methoxybenzyl, 4-bromobenzyl or 4-nitrobenzoyl.

Our synthetic and computational interest in 2,2′-bifuranyl natural products coupled with their embedded *C*_2_ symmetry made laurefurenynes A and B (**1**) attractive targets for total synthesis. As part of our structure determination research program, we had synthesized the 2,2′-bifuranyl **15** with the same relative configuration as the originally proposed structures for laurefurenynes A and B (**1**), along with two further related 2,2′-bifuranyls **16** and **18** (Scheme 1). The synthesis of the 2,2′-bifuranyls **15**, **16** and **18** followed a similar course to our recently reported synthesis of elatenyne **3 a**.^8^ Self-metathesis of the known epoxy alkene **6**^14^ by using Grubbs’ second generation catalyst **19** in the presence of acetic acid to minimize isomerization of the starting material,^15^ followed by an oxidative workup to remove ruthenium residues^16^ gave a 3:1 mixture of partially separable *E*/*Z* geometric isomers **7** and **8** in 67 % yield. Diastereoselective Sharpless dihydroxylation^17^ of the pure (*E*)-alkene **7** with the hydroquinine 1,4-phthalazinediyl diether ((DHQ)_2_PHAL) ligand gave a 3.5:1 mixture of the diols **9** and **10** along with a small amount of the corresponding cyclized material in 97 % overall yield. Separation of the individual diols **9** and **10** was not possible, because silica gel caused further cyclisation to give the highly polar 2,2′-bifuranyls **11** and **12**. Cyclisation was further promoted by the use of Amberlyst acidic resin to give a mixture of 2,2′-bifuranyls which were immediately converted into the separable dimesylates **13** and **14**. The major dimesylate **13** could be readily converted into the model 2,2′-bifuranyl **15** by reduction with Superhydride followed by hydrogenolysis of the benzyl protecting groups. We also prepared two related 2,2′-bifuranyls **16** and **18** by Sharpless dihydroxylation^17^ of the alkene **7** using the (DHQD)_2_PHAL ligand (1:7 mixture **9**:**10**, and some cyclized material), and from the (*Z*)-alkene **8** according to the routes shown in Scheme 1. Comparison of the ^13^C NMR spectra of the three model 2,2′-bifuranyls with those of the natural product led us to question the assigned stereostructure of laurefurenynes A and B (**1**). In particular, we noted that with the asymmetric 2,2′-bifuranyl **18** derived from the (*Z*)*-*alkene **8**, the ^13^C NMR chemical shifts of the ring methylene carbons were more in keeping with those of the natural products than for the *C*_2_-symmetric derivatives **15** and **16**.^18^ As part of our work on the structure determination of elatenyne **3 a**, we had also synthesized several 2,2′-bifuranyls with hydroxyl groups at C-7 and C-12 (laurefurenyne numbering);^8^ close structural analogues of laurefurenynes A and B (**1**). Examination of the ^13^C NMR chemical shifts of seven synthetic 2,2′-bifuranyls indicated that when the hydroxyl group and the adjacent side chain are *cis*-related, C–OH resonates at *δ*≈71 ppm; however, when the hydroxyl group and the adjacent side chain are *trans*-related, C–OH resonates at *δ*≈75–76 ppm (Figure 2).^19^

This is clearly a small sample; however, comparison with the reported ^13^C NMR chemical shifts of laurefurenynes A and B was informative. The relevant ^13^C NMR chemical shifts in laurefurenynes A and B occur at *δ*≈75 ppm (C-7, CDCl_3_ or [D_6_]DMSO) and *δ*≈71 ppm (C-12, CDCl_3_ or [D_6_]DMSO), respectively. On the basis of these data, we propose that laurefurenynes A and B (**1**) are not pseudo-*C*_2_ symmetric, and specifically that the C-6/C-7 substituents are *trans*-disposed (rather than *cis*-disposed) with the C-12/C-13 substituents being *cis*-disposed.

In tandem with our synthetic studies, we also turned to quantum-chemical calculations to compare the predicted ^1^H and ^13^C NMR data of the reported structures for laurefurenynes A and B against the experimental values obtained for the natural products.^20^ The relative accuracy and affordability of ^1^H and ^13^C NMR chemical shifts obtained from DFT data means that such calculations are increasingly used to probe and validate structural hypotheses for small to medium-sized organic molecules.^21^, ^22^ To benchmark our calculations, we computed GIAO ^13^C NMR chemical shifts for 113 rigid small molecules containing only C, H, N, O and F^23^ at the mPW1PW91/6-311G(d,p)/wB97XD/6-31G(d) level, which (following linear scaling)^21^ gave a small mean unsigned error (MUE) of 1.6 ppm and standard deviation of 1.5 ppm with respect to experiment demonstrating the accuracy of the technique. Nevertheless, laurefurenynes A and B pose a considerable challenge for computation, in large part due to the flexibility of the two rings and freely rotatable single bonds, which give rise to large numbers of thermally accessible conformers that must be taken into consideration. Rotation about the central inter-ring torsion also makes the determination of relative stereochemistry of the two THF rings difficult. Given these computationally challenging molecules prompted us to examine whether the sensitivity of DFT-computed chemical shifts is sufficient to discriminate between correct and incorrect structures by using various metrics. In fact, as is described below, our computations cast doubt over the previous assignment and accurately predicted the correct stereostructure **5 b** for laurefurenyne B.

**Figure 3:**
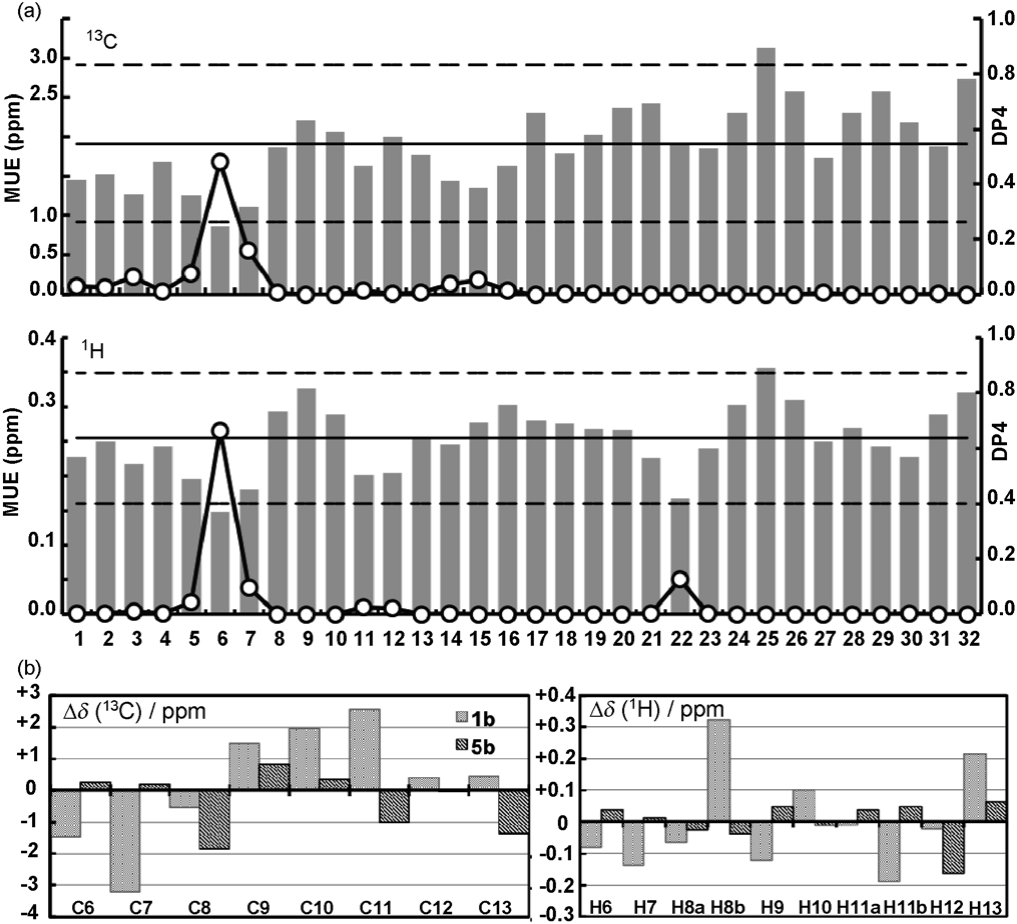
a) Mean unsigned errors (ppm, bars) and DP4 probability (white circles) for the 32 diastereomers of 1 b with respect to the natural-product data. Horizontal lines show mean error±two standard deviations. b) ^13^C and ^1^H NMR errors, *δ*_calcd_−*δ*_exptl_ (ppm) for the central ring regions of structures 1 b and 5 b.

For each of the 32 possible diastereomers of laurefurenyne B, we carried out a Monte Carlo multiple minimum (MCMM)^24^ conformational search with MMFF^25^ and subsequently reoptimized all low energy conformers (within 10 kJ mol^−1^) at the dispersion-corrected DFT, wB97XD/6-31G(d), level in CHCl_3_.^26^ This choice was motivated by the observation that the potential energy hypersurface is characterized by intramolecular hydrogen bonding and medium and long-range non-bonding interactions. The number of conformers for each diastereomer ranges from 10 to 167, for which ^13^C NMR and ^1^H NMR GIAO-mPW1PW91/6-311G(d,p) chemical shifts were calculated in CHCl_3_.^27^ For comparison against experimental values, the average isotropic shielding tensors for the conformational ensemble was computed using Boltzmann factors from the electronic energies at 298 K; conversion into chemical shifts was performed following a linear regression against the experimental data.^21^ Chemical shifts for pairs of diastereotopic protons were automatically assigned so as to minimize the computational errors.^28^ Computed mPW1PW91/6-311G(d,p)//wB97XD/6-31G(d) chemical shifts for all 32 diastereomers of **1 b** were compared against the natural product data along with DP4 analysis (Figure 3 a).^11^, ^29^

Across all diastereomers, the MUEs span the range of *δ*=0.9–3.1 ppm (^13^C) and 0.15–0.36 ppm (^1^H). Linear regression gives *R*^2^ values all close to unity (see the Supporting Information).^21^ The previous stereochemical assignment, **1 b**, which corresponds to diastereomer #1, has an MUE of 1.5 and 0.23 ppm for ^13^C and ^1^H NMR shifts, respectively. In contrast to the MUE, the DP4 metric rules out structures from consideration that have one or more significant errors in predicted ^1^H and/or ^13^C NMR chemical shift; under this metric diastereomer #1 is highly unlikely to be correct (Figure 3 a). Computationally, structure **5 b** (diastereomer #6 in the computational studies) has the smallest errors for ^13^C and ^1^H chemical shifts and correspondingly the highest DP4 probability (Figure 3 a and b). In accord with our experimental observations, the C6-C7 *cis*-stereochemistry in **1 b** leads to a computed ^13^C NMR chemical shift value at C-7 that is >3 ppm below that of C-7 of the natural product. This relationship between relative configuration and chemical shift is true across all 32 computed diastereomers. All of our synthetic and computational data gave us confidence that the actual stereostructures of laurefurenynes A and B are as represented by **5**.

**Scheme 2:**
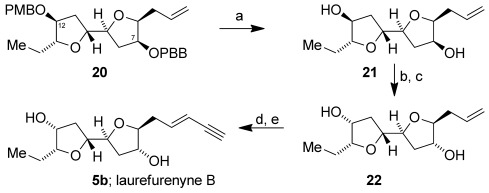
Total synthesis of laurefurenyne B 5 b. a) BCl_3_, CH_2_Cl_2_, 94 %; b) DIAD, Ph_3_P, *para*-nitrobenzoic acid, THF, 0 °C→RT; c) K_2_CO_3_, MeOH, 75 % from 21; d) crotonaldehyde, Grubbs II 19, CH_2_Cl_2_, 40 °C, 1.5 h, then DMSO, RT, 16 h; e) TMSCH_2_N_2_, LDA, THF, −78 to 0 °C, 1.5 h, then HCl (2 m), 45 % from 22.

We had previously prepared the protected 2,2′-bifuranyl **20** as an intermediate en route to elatenyne.^8^ Converting this bis-benzyl ether into the reassigned structure of laurefurenyne B required inversion of configuration at both C-12 and C-7. Deprotection of both the 4-methoxybenzyl (PMB) and the 4-bromobenzyl (PBB) groups in **20** was readily achieved by using boron trichloride,^8^ and the resultant diol **21** was inverted at C-7 and C-12 (laurefurenyne numbering) by using a Mitsunobu reaction giving **22** (Scheme 2). Conversion of the alkene **22** into a terminal *E*-enyne was readily achieved by using Kim’s method^8^, ^30^ by cross metathesis with crotonaldehyde followed by Colvin–Ohira homologation. The ^1^H and ^13^C NMR spectra of synthetic **5 b** were in excellent agreement with the corresponding reported data for natural laurefurenyne B. This work defines the stereostructures of laurefurenynes A and B as **5**. Moreover, the optical rotation of synthetic **5 b** ([*α*]_D_^20^ −20 (*c*=0.1 in MeOH)) was in agreement with that of natural laurefurenyne B ([*α*]_D_^20^ −13 (*c*=0.1 in MeOH)),^1^ indicating that the likely absolute configurations of laurefurenynes A and B are represented by **5** (Figure 1).

**Scheme 3:**
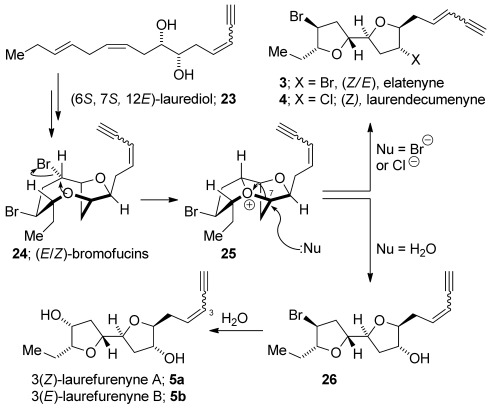
Plausible biogenesis of laurefurenynes A and B (30).

We recently proposed a biosynthesis of (*E*)- and (*Z*)-elatenyne **3** and laurendecumenyne **4**^8^ closely paralleling previous work on the biogenesis of C_15_ halogenated marine natural products from *Laurencia* spp.^10^ Close inspection of the stereostructures of laurefurenynes A and B indicates that they may be biosynthesized similarly from (*E*)-31, 32 or (*Z*)-bromofucin **24**,^33^ which may be biosynthesized from (3*E*/*Z*,6*S*,7*S*,12*E*)-laurediol **23** (Scheme 3).^34^, ^35^ Transannular expulsion of bromide leads to the tricyclic oxonium ion **25** that may be opened by bromide, to give elatenyne **3** or by chloride to give laurendecumenyne **4**.^36^, ^37^ Opening of the same oxonium ion **25** by water would give **26** with displacement of the bromide by water^38^ giving laurefurenynes A and B **5**. In terms of natural products, the missing links on the proposed biosynthetic pathway towards the laurefurenynes are the bromoalcohols **26**, which we postulate as yet-to-be-isolated natural products. This biogenesis places laurefurenynes A and B in the same absolute stereochemical series as that proposed for (*Z*)-elatenyne.^8^

In summary, reassignment of the stereostructure of laurefurenynes A and B was achieved on the basis of close analysis of NMR data in model compounds and DFT calculations of NMR chemical shifts. Total synthesis of the proposed structure of laurefurenyne B confirmed the reassigned structures, which places laurefurenynes on the same biosynthetic pathway, as was recently proposed for elatenyne. This work further highlights the difficulty of unambiguously assigning relative configuration in highly flexible organic molecules by using NMR methods, and the power of a combined computational/synthetic approach for structure determination. Further application of this approach to the structure determination of small molecules along with a full discussion of the computational aspects of this work will be reported in due course.
