# Supplementary material for: Structure Reassignment of Laurefurenynes A and B by Computation and Total Synthesis
Source: Chemistry. 2013 Aug 21;19(38):12644–8. doi: 10.1002/chem.201302349 (PMC4280896; doi:10.1002/chem.201302349)
Supplement: Supplementary file 1 [file chem0019-12644-SD1.pdf]

CHEMISTRY
A EUROPEAN JOURNAL

Supporting Information

© Copyright Wiley-VCH Verlag GmbH & Co. KGaA, 69451 Weinheim, 2013

Structure Reassignment of Laurefurenynes A and B by Computation and Total Synthesis

David J. Shepherd, Phillip A. Broadwith, Bryony S. Dyson, Robert S. Paton,* and Jonathan W. Burton*[a]

chem_201302349_sm_miscellaneous_information.pdf

Structure Reassignment of Laurefurenynes A and B by Computation and Total Synthesis

David J. Shepherd, Phillip A. Broadwith, Bryony S. Dyson, Robert S. Paton and Jonathan W. Burton**

robert.paton@chem.ox.ac.uk / paton.chem.ox.ac.uk / jonathan.burton@chem.ox.ac.uk / burton.chem.ox.ac.uk
Chemistry Research Laboratory, Mansfield Road, Oxford OX1 3TA, UK

Table of Contents:

Synthetic Supporting Information 2

1.1 General 2
1.2 Experimental Procedures and Data 3
1.3 Figures and Tables 17
1.4 Synthetic References 20
1.5 ^{1}H NMR and ^{13}C NMR Spectra 21

Computational Supporting Information 55

2.1 Benchmarking the GIAO calculations 55
2.2 Computational Protocol for Predicting the Structure of Laurefurenynes A and B 57
2.3 Results from chemical shift comparison with Laurefurenyne B 58
2.4 Are DFT Optimizations Necessary? 59
2.5 Comparison of the previous and reassigned structures of Laurefurenyne B 59
2.6 Ensemble Averaged Shielding Tensors and Chemical Shifts 60
2.7 Python Scripts for the Automated Analysis of Multiple Conformations 63
2.8 Computational References 75

Synthetic Supporting Information

1.1 General

Proton (^{1}H) and carbon (^{13}C) NMR spectra were recorded on a Bruker AV 500 (500/125 MHz), Bruker AV 400 (400/100 MHz) or Bruker DPX 200 (200/50 MHz) spectrometer. Proton and carbon chemical shifts (δ_H, δ_C) are quoted in ppm and referenced to tetramethylsilane with residual protonated solvent as internal standard. Resonances are described as s (singlet), d (doublet), t (triplet), q (quartet), m (multiplet), br (broad), dd (double doublet) and so on. Coupling constants (*J*) are given in Hz and are rounded to the nearest 0.1 Hz. H and H' refer to diastereotopic protons attached to the same carbon and imply no particular stereochemistry. CHOR refers to bridgehead moieties and in the 2,2'-bifuranyls **13-16**, **18**, **20-22** and **5b** assignments should be read from the leftmost to rightmost side of the compound as drawn. Low resolution mass spectra were recorded on a Fisons Platform spectrometer (ES). High resolution mass spectra were recorded by the mass spectrometry staff at the Chemistry Research Laboratory, University of Oxford, using a Bruker Daltronics microTOF spectrometer (ES) or a Micromass GCT (FI). *m/z* values are reported in Daltons with their percentage abundances and, where known, the relevant fragment ions in parentheses. High resolution values are calculated to four decimal places from the molecular formula, all found values being within a tolerance of 5 ppm. Infrared spectra were recorded on a Bruker Tensor 27 Fourier Transform spectrometer, as a thin film on diamond ATR. Absorption maxima (ν_{max}) are quoted in wavenumbers (cm^{-1}). Optical rotations were measured using a Perkin-Elmer 241 polarimeter in a cell of 1 dm path length (l). TLC was performed on Merck DC-Alufolien 60F254 0.2 mm precoated plates and visualised using an acidic vanillin or basic potassium permanganate dip. Retention factors (R_f) are reported with the solvent system used in parentheses. Flash column chromatography was performed on Merck 60 silica (particle size 40-63 μm, pore diameter 60 Å) and the solvent system used is recorded in parentheses. $AgNO_3$ impregnated silica and TLC plates were prepared according to a modified procedure of Li *et al.*[1]

All non-aqueous reactions were carried out in oven-dried glassware under an inert atmosphere of nitrogen and employing standard techniques for handling air-sensitive materials. Solvents and commercially available reagents were dried and purified before use, as appropriate. In particular DCM and THF were distilled from CaH_2 and stored over 3 Å molecular sieves. 'Petrol' refers to the fraction of light petroleum ether boiling in the range 40-60 °C unless otherwise stated. All water used

experimentally was distilled and the term 'brine' refers to a saturated solution of sodium chloride in water.

1.2 Experimental Procedures and Data

***(1S,6S,E)-1,6-Bis(benzyloxy)-1,6-di((R)-oxiran-2-yl)hex-3-ene* 7**

***(1S,6S,Z)-1,6-Bis(benzyloxy)-1,6-di((R)-oxiran-2-yl)hex-3-ene* 8**

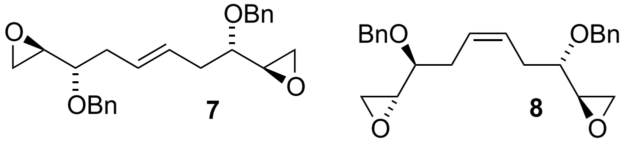

(*R*)-2-((*S*)-1-(Benzyloxy)but-3-enyl)oxirane **6**[2] (>95% ee, 950 mg, 4.66 mmol) and acetic acid (28 mg, 27 mL, 0.46 mmol) were dissolved in degassed CH_2Cl_2 (6 mL) and heated to reflux. A solution of Grubbs' second generation catalyst (40 mg, 0.05 mmol)[3] in CH_2Cl_2 (1 mL) was added dropwise over 6 h and the mixture heated for a further 10 h. The reaction mixture was cooled to room temperature, diluted with CH_2Cl_2 (45 mL) and stirred with 15% aq. hydrogen peroxide (50 mL),[4] cooling with ice as required until effervescence ceased. The phases were separated and the organic phase washed with sat. aq. $Na_2S_2O_3$ (50 mL), dried ($MgSO_4$), filtered, concentrated *in vacuo* and purified by flash column chromatography (10→20% ethyl acetate in petrol) gave the product as a colorless liquid (594 mg, 1.56 mmol, 67%, ~3:1 *E/Z* ratio) along with returned starting material (247 mg, 1.21 mmol, 26%). Further chromatographic purification on silver nitrate-doped silica[1] (15:1 CH_2Cl_2:ethyl acetate) allowed partial separation of the *E* and *Z* alkenes. The structural assignment of the two alkene geometric isomers was made by ^{13}C NMR chemical shifts of the allylic carbon nuclei. The allylic carbon atoms of (*Z*)-disubstituted alkenes generally resonate at lower chemical shift (4-6 ppm) compared with the corresponding carbon atoms of the (*E*)-disubstituted alkenes. The chemical shifts of the allylic carbon atoms of **8** were at δ_C = 30.8 ppm, and for **7** were at δ_C = 36.0 ppm.

E alkene **7**: R_f 0.17 (4:1 petrol:ethyl acetate); ν_{max}/ cm^{-1} (thin film) 3032, 2995, 2920, 2867(C-H), 1606 (w, C=C); δ_H (400 MHz $CDCl_3$) 7.36-7.27 (10H, m, Ar*H*), 5.69-5.60 (2H, m, (C=C*H*), 4.63 (2H, d, *J* = 11.8 Hz, C*H*H'Ar), 4.53 (2H, d, *J* = 11.8 Hz, OCH*H'*Ar), 3.30 (2H, dt, *J* = 6.6, 5.3 Hz, C*H*OBn), 2.97 (2H, ddd, *J* = 5.3, 3.9, 2.7 Hz, C*H*OCH2), 2.77 (2H, dd, *J* = 5.3, 3.9 Hz, CHOC*H*H'), 2.72 (2H, dd, *J* = 5.3, 2.7 Hz, CHOCH*H'*), 2.47-2.35 (4H, dt, *J* = 9.9, 5.4 Hz, C*H*2CH=CH); δ_C (125 MHz $CDCl_3$) 138.4 (Ar), [128.4, 128.3, 127.6, 127.6 (3 × Ar and C=C)], 77.9 (*C*HOBn), 72.1 (*C*H2Ph), 53.1 (*C*HOCH2),

45.6 (CHO*C*H_2), 36.0 (*C*H_2); *m/z* (ES^+) 403 ($M+Na^+$, 100), Accurate mass (ES^+): found 403.1875, $C_{24}H_{28}O_4Na^+$ ($M+Na^+$), requires 403.1880; $[\alpha]_D^{20}$ +1.4 (*c* = 0.35 in CH_2Cl_2).

Z alkene **8**: R_f 0.17 (4:1 petrol:ethyl acetate); ν_{max}/ cm^{-1} (thin film) 3028, 2992, 2923, 2862 (C-H), 1630 (C=C); δ_H (400 MHz $CDCl_3$) 7.36-7.26 (10H, m, Ar*H*), 5.71-5.63 (2H, m, (C=C*H*), 4.68 (2H, d, *J* = 11.8 Hz, C*H*H'Ar), 4.52 (2H, d, *J* = 11.7 Hz, CH*H'*Ar), 3.33 (2H, dt, *J* = 6.5, 5.4 Hz, C*H*OBn), 2.96 (2H, ddd, *J* = 5.4, 3.8, 2.7 Hz, C*H*OCH_2), 2.76 (2H, dd, *J* = 5.3, 3.9 Hz, CHOC*H*H'), 2.72 (2H, dd, *J* = 5.3, 2.6 Hz, CHOCH*H'*), 2.50-2.41 (4H, t, *J* = 5.9 Hz, CH_2CH=CH); δ_C (62.5 MHz $CDCl_3$) 138.4 (Ar), [128.3, 127.6, 127.0 (Ar and C=C)], 77.8 (*C*HOBn), 72.1 (*C*H_2Ph), 53.1 (*C*HOCH_2), 45.6 (CHO*C*H_2), 30.8 (*C*H_2); *m/z* (ES^+) 403 ($M+Na^+$, 100); Accurate mass (ES^+): found 403.1877, $C_{24}H_{28}O_4Na^+$ ($M+Na^+$), requires 403.1880; $[\alpha]_D^{20}$ +12.3 (*c* = 0.27 in CH_2Cl_2).

***(1S,3S,4S,6S)-1,6-Bis(benzyloxy)-1,6-di((R)-oxiran-2-yl)hexane-3,4-diol* 9**

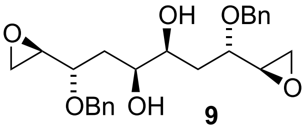

9

(1*S*,6*S*,*E*)-1,6-Bis(benzyloxy)-1,6-di((*R*)-oxiran-2-yl)hex-3-ene **7** (300 mg, 0.79 mmol), methanesulfonamide (75 mg, 0.79 mmol), $(DHQ)_2$PHAL (12 mg, 16 mmol), potassium hexacyanoferrate(III) (780 mg, 2.37 mmol) and potassium carbonate (328 mg, 2.37 mmol) were dissolved in a mixture of *tert*-butanol and water (1:1 v/v, 6 mL) and cooled to 0 °C with vigorous stirring. Potassium osmate dihydrate (3 mg, 8 mmol) was added and the reaction stirred for 16 h at 0 °C. Sodium sulfite (500 mg) was added and the reaction stirred at room temperature for 30 min. The mixture was diluted with sufficient water to just dissolve any solid residue (~1-2 mL) and extracted with ethyl acetate (3 × 15 mL). The combined organic phases were washed with aq. NaOH (0.1 M, 15 mL) which was back-extracted with ethyl acetate (15 mL). The combined organics were dried ($MgSO_4$), filtered and concentrated *in vacuo*. The crude residue was filtered through a plug of silica, flushing with neat ethyl acetate to give the product as a mixture of diastereomers along with some cyclised material (316 mg, 0.77 mmol, ~97%). The data below are obtained on the crude material as attempted purification by flash chromatography caused further cyclisation occur. The structural assignment of the diols was made on the basis of the Sharpless mnemonic.[5] Data for **9**: R_f 0.2 (ethyl acetate); ν_{max}/ cm^{-1} (thin film) 3441 (br, O-H), 3061, 3030, 2923, 2864 (C-H), 1496, 1453; δ_H (500

MHz $CDCl_3$) 7.45-7.29 (10H, m, Ar*H*), 4.75 (2H, d, *J* = 11.4 Hz, C*H*H'Ar), 4.56 (2H, d, *J* = 11.4 Hz, CH*H'*Ar), 3.79-3.73 (2H, br d, *J* = 9.3 Hz, C*H*OH), 3.70 (2H, ddd, *J* = 8.5, 5.2, 3.2 Hz, C*H*OBn), 3.03 (2H, m, C*H*OCH_2), 2.86 (2H, ddd, *J* = 5.2, 3.9, 0.6 Hz, CHOC*H*H'), 2.80 (2H, dd, *J* = 5.4, 2.7 Hz, CHOCH*H'*), 1.94 (2H, ddd, *J* = 14.5, 9.8, 3.3 Hz, C*H*H'CHOH), 1.87 (2H, ddd, *J* = 14.5, 8.6, 2.2 Hz, CH*H'*CHOH); *m/z* (ES^+) 437 ($M+Na^+$, 100); Accurate mass (ES^+): found 437.1944, $C_{24}H_{30}O_6Na^+$ ($M+Na^+$), requires; optical rotation not reported as analysis was on crude material.

***((2S,2'S,4S,4'S,5S,5'S)-4,4'-Bis(benzyloxy)octahydro-2,2'-bifuran-5,5'-diyl)bis(methylene) dimethanesulfonate* 13**

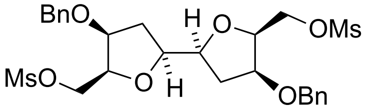

13

Part of the product of the previous reaction (130 mg, 0.31 mmol) was dissolved in $CDCl_3$ (3 mL) and a small amount of Amberlyst-15® resin added. The reaction was stirred at room temperature for 20 min before being filtered, concentrated *in vacuo* and purified by flash column chromatography (5% methanol in ethyl acetate) to give an inseparable mixture of two diastereomeric cyclised diols (**11** and **12**). This mixture was dissolved in CH_2Cl_2 (5 mL), cooled to 0 °C and triethylamine (385 mg, 530 mL, 3.80 mmol) added, followed by dropwise addition of methanesulfonyl chloride (290 mg, 200 mL, 2.53 mmol). The stirred reaction mixture was allowed to warm to room temperature over 16 h before being quenched with aq. HCl (2 M, 5 mL) and extracted with CH_2Cl_2 (2 × 10 mL). The combined organics were dried ($MgSO_4$), filtered and concentrated *in vacuo*. Purification by flash column chromatography (15:1→4:1 CH_2Cl_2:ethyl acetate) gave the product **13** as colorless oil (105 mg, 0.18 mmol, 72%) along with the diastereomer corresponding to opposite facial selectivity in the SAD reaction **14** (25 mg, 0.05 mmol, 20%); see below for independent synthesis of the minor diastereoisomer **14**, ((2*R*,2'*R*,4*S*,4'*S*,5*S*,5'*S*)-4,4'-bis(benzyloxy)octahydro-2,2'-bifuran-5,5'-diyl)bis(methylene) dimethanesulfonate).

Data for **13**: R_f 0.16 (1:1 petrol:ethyl acetate); ν_{max}/ cm^{-1} (thin film) 3029, 2936, 2867 (C-H), 1498, 1453, 1352, 1171 ($O-SO_2$); δ_H (400 MHz $CDCl_3$) 7.38-7.26 (10H, m, Ar*H*), 4.54 (2H, d, *J* = 11.8 Hz, C*H*H'Ph), 4.48 (2H, dd, *J* = 11.0, 4.2 Hz, C*H*H'OMs), 4.42 (2H, dd, *J* = 11.0, 6.7 Hz, CH*H'*OMs), 4.39 (2H, d, *J* = 11.5 Hz, CH*H'*Ph), 4.21 (2H, ddd, *J* = 6.3, 5.4, 4.2 Hz, C*H*OBn), 4.15 (2H, ddd, *J* =

6.7, 5.4, 4.3 Hz, C*H*CH_2OMs), 4.12-4.07 (2H, m, C*H*OR), 2.99 (6H, s, SO_2*Me*), 2.22-2.16 (2H, m, C*H'*HCHOBn), 1.87 (2H, dddd, *J* = 13.4, 5.6, 4.1, 1.8 Hz, CH*H'*CHOBn); δ_C (62.5 MHz $CDCl_3$); 137.4 (Ar), 128.5 (Ar), 127.9 (Ar), 127.5 (Ar), 80.2 (*C*HOBn) 79.2 (*C*HOR), 78.3 (*C*HCH_2OMs), 71.4 (*C*H_2Ph), 69.0 (*C*H_2SO_2Me), 37.4 (SO_2*Me*), 33.7 (*C*H_2CHOBn); *m/z* (ES^+) 593 ($M+Na^+$, 100); Accurate mass (ES^+): found 593.1483, $C_{26}H_{34}O_{10}S_2Na^+$ ($M+Na^+$), requires; $[\alpha]_D^{20}$ +25.1 (*c* = 0.35 in CH_2Cl_2).

(2S,2'S,4S,4'S,5S,5'S)-4,4'-Bis(benzyloxy)-5,5'-dimethyloctahydro-2,2'-bifuran

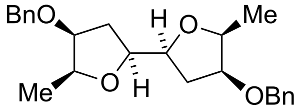

((2*S*,2'*S*,4*S*,4'*S*,5*S*,5'*S*)-4,4'-Bis(benzyloxy)octahydro-2,2'-bifuran-5,5'-iyl)bis(methylene) dimethanesulfonate **13** (126 mg, 0.22 mmol) was dissolved in THF (2 mL), cooled to 0 °C and SuperHydride™ (2.2 mL of a 1.0 M solution in diethyl ether, 2.2 mmol) was added dropwise. The reaction was warmed to room temperature and stirred for 16 h before being quenched with sat. aq. NH_4Cl (5 mL). The mixture was diluted with water until the precipitated salts dissolved (<1 mL). The phases were separated and the aqueous extracted with ethyl acetate (2 × 10 mL), the combined organic phases were dried ($MgSO_4$), filtered and concentrated *in vacuo*. Purification of the residue by flash column chromatography (1:4→1:1 ethyl acetate:petrol) gave the product as a colorless oil (67 mg, 0.18 mmol, 80%). R_f 0.56 (1:1 petrol:ethyl acetate); ν_{max}/ cm^{-1} (thin film) 3063, 3031, 2976, 2933, 2862 (C-H), 1497, 1451; δ_H (500 MHz $CDCl_3$) 7.35-7.26 (10H, m, Ar*H*), 4.54 (2H, d, *J* = 12.3 Hz, C*H*H'Ph), 4.41 (2H, d, *J* = 12.3 Hz, CH*H'*Ph), 3.98-3.90 (6H, m, MeC*H*C*H*OBnCH_2C*H*)), 2.21-2.16 (2H, m, C*H*H'), 1.76 (2H, dddd, *J* = 13.4, 5.3, 3.3, 1.7 Hz, CH*H'*), 1.35 (6H, d, *J* = 6.0 Hz, *Me*); δ_C (125 MHz $CDCl_3$) 138.5 (Ar), 128.3 (Ar), 127.4 (Ar), 127.1 (Ar), 80.1 (*C*HOBn), 79.5 *C*HOR), 78.5 (Me*C*H), 71.0 (*C*H_2Ph), 34.6 (*C*H_2), 14.8 (*Me*); *m/z* (ES^+) 405 ($M+Na^+$, 100); Accurate mass (ES^+): found 405.2027, $C_{24}H_{30}O_4Na^+$ ($M+Na^+$), requires; $[\alpha]_D^{20}$ +56.3 (*c* = 0.65 in MeOH).

***(2S,2'S,4S,4'S,5S,5'S)-5,5'-Dimethyloctahydro-2,2'-bifuran-4,4'-diol* 15**

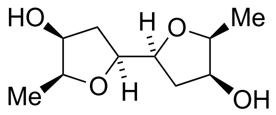

15

(2*S*,2'*S*,4*S*,4'*S*,5*S*,5'*S*)-4,4'-Bis(benzyloxy)-5,5'-dimethyloctahydro-2,2'-bifuran (57 mg, 155 mmol) was dissolved in ethanol (5 mL) and 10% palladium on charcoal (8 mg, 8 mmol Pd) was added. The vessel was evacuated and back-filled with nitrogen three times, then hydrogen three times and the reaction stirred at room temperature under hydrogen atmosphere for 16 h. The atmosphere was exchanged back to nitrogen and the reaction mixture filtered through Celite®, flushing with ethyl acetate. The solvent was removed *in vacuo* to give the product as a colorless oil (32 mg, 155 mmol, quant.). R_f 0.07 (ethyl acetate); ν_{max}/ cm^{-1} (thin film) 3363 (O-H), 2985, 2924, 2851 (C-H); δ_H (500 MHz $CDCl_3$) 4.17 (2H, d, *J* = 11.1 Hz, O*H*), 4.11 (2H, dd, *J* = 10.1, 2.3 Hz, C*H*OR), 3.92-3.88 (4H, m, MeC*H*C*H*OH), 2.40 (2H, ddd, *J* = 14.1, 10.5, 5.1 Hz, C*H*H'), 2.02 (2H, ddd, *J* = 14.1, 2.5 Hz, CH*H'*), 1.34 (6H, d, *J* = 6.1 Hz, *Me*); δ_C (125 MHz $CDCl_3$) 81.0, 79.1 (*C*HOR), 72.0 (Me*C*H), 37.9 (*C*H_2), 13.7 (*Me*); *m/z* (ES^+) 225 ($M+Na^+$, 100); Accurate mass (ES^+): found 225.1102, $C_{10}H_{18}O_4Na^+$ ($M+Na^+$), requires 225.1097; $[\alpha]_D^{20}$ +21.5 (*c* = 1.0 in MeOH).

***((2R,2'R,4S,4'S,5S,5'S)-4,4'-Bis(benzyloxy)octahydro-2,2'-bifuran-5,5'-diyl)bis(methylene) dimethanesulfonate* 14**

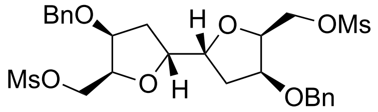

14

(1*S*,6*S*,*E*)-1,6-Bis(benzyloxy)-1,6-di((*R*)-oxiran-2-yl)hex-3-ene **7** (240 mg, 0.63 mmol), methanesulfonamide (60 mg, 0.63 mmol), $(DHQD)_2PHAL$ (10 mg, 13 mmol), potassium hexacyanoferrate(III) (623 mg, 1.89 mmol) and potassium carbonate (261 mg, 1.89 mmol) were dissolved in a mixture of *tert*-butanol and water (1:1 v/v, 6 mL) and cooled to 0 °C with vigorous stirring. Potassium osmate dihydrate (3 mg, 6 mmol) was added and the reaction stirred for 16 h at 0 °C. Sodium sulfite (500 mg) was added and the reaction stirred at room temperature for 30 min. The

mixture was diluted with sufficient water to just dissolve any solid residue (~1-2 mL) and extracted with ethyl acetate (3 × 15 mL). The combined organic phases were washed with aq. NaOH (0.1 M, 15 mL) which was back-extracted with ethyl acetate (15 mL). The combined organics were dried ($MgSO_4$), filtered and concentrated *in vacuo*. The crude residue was filtered through a plug of silica, flushing with neat ethyl acetate, to give the product as a mixture of diastereomers along with some cyclised material, which was used directly in the next reaction (266 mg, ~quant). The data below are obtained on the crude material as attempted purification by flash chromatography caused further cyclisation occur. The structural assignment of the diols was made on the basis of the Sharpless mnemonic.[5] Data for **10**: R_f 0.17 (1:2 petrol:ethyl acetate); ν_{max}/ cm^{-1} (thin film) 3412 (O-H), 3031, 2923, 2866 (C-H), 1497, 1454; δ_H (500 MHz $CDCl_3$) 7.44-7.31 (10H, m, Ar*H*), 4.77 (2H, d, *J* = 11.4 Hz, C*H*H'Ar), 4.54 (2H, d, *J* = 11.4 Hz, OCH*H*'Ar), 3.79-3.73 (2H, m, C*H*OH), 3.56 (2H, ddd, *J* = 8.5, 5.4, 4.5 Hz, C*H*OBn), 3.35 (2H, d, *J* = 3.2 Hz, O*H*), 3.01 (2H, ddd, *J* = 5.5, 3.8, 2.6 Hz, C*H*OCH$_2$), 2.86 (2H, dd, *J* = 5.2, 3.8 Hz, CHOC*H*H'), 2.79 (2H, dd, *J* = 5.2, 2.7 Hz, CHOCH*H'*), 1.94 (2H, ddd, *J* = 14.2, 4.2, 3.1 Hz, C*H*H'), 1.87 (2H, dd, *J* = 14.2, 8.6 Hz, CH*H'*); *m/z* (ES$^+$) 437 (M+Na$^+$, 100); Accurate mass (ES$^+$): found 437.1933, $C_{24}H_{30}O_6Na^+$ (M+Na$^+$), requires 437.1935; optical rotation not reported as analysis was on crude material.

The product of the previous reaction (210 mg, 0.51 mmol) was dissolved in $CDCl_3$ (5 mL) and a small amount of Amberlyst-15® resin added. The reaction was stirred at room temperature for 20 min before being filtered, concentrated in vacuo and purified by flash column chromatography (5% methanol in ethyl acetate) to give an inseparable mixture of cyclised diols. This mixture was dissolved in CH_2Cl_2 (10 mL), cooled to 0 °C and triethylamine (775 mg, 1.07 mL, 7.61 mmol) added, followed by dropwise addition of methanesulfonyl chloride (581 mg, 392 mL, 5.07 mmol). The stirred reaction mixture was allowed to warm to room temperature over 16 h before being quenched with aq. HCl (2 M, 10 mL) and extracted with CH_2Cl_2 (2 × 15 mL). The combined organics were dried ($MgSO_4$), filtered and concentrated *in vacuo*. Purification by flash column chromatography (15:1→4:1 CH_2Cl_2:ethyl acetate) gave the product **14** as colorless oil (240 mg, 0.42 mmol, 83%) along with the diastereomer corresponding to opposite facial selectivity in the SAD reaction **13** (35 mg, 0.06 mmol, 12%) indicating approximately 7:1 diastereoselectivity in this case (see above for independent synthesis of the minor diastereoisomer, ((2*S*,2'*S*,4*S*,4'*S*,5*S*,5'*S*)-4,4'-bis(benzyloxy)octahydro-2,2'-bifuran-5,5'-diyl)bis(methylene) dimethanesulfonate).

Data for **14**: R$_f$ 0.18 (1:1 petrol:ethyl acetate); ν_{max}/ cm^{-1} (thin film); δ_H (500 MHz $CDCl_3$) 7.38-7.34 (4H, m, Ar*H*), 7.33-7.29 (6H, m, Ar*H*), [4.61 (2H, d, *J* = 11.7 Hz, C*H*H'Ar), 4.44 (2H, d, *J* = 11.7 Hz, (CH*H'*Ph), 4.42 (2H, dd, *J* = 10.7, 4.4 Hz, C*H*H'OMs), 4.38 (2H, dd, *J* = 10.7, 6.2 Hz, CH*H'*OMs), 4.28-4.23 (4H, m, C*H*OBnC*H*), 4.22-4.17 (2H, m, C*H*OR), 2.98 (6H, s, SO_2*Me*), 2.16 (2H, ddd, *J* = 13.2, 6.3, 1.6 Hz, C*H*H'CHOBn), 2.07-2.00 (2H, m, CH*H'*CHOBn); δ_C (125 MHz $CDCl_3$) 137.6 (Ar), 128.5 (Ar), 127.9 (Ar), 127.6 (Ar), 79.7 (*C*HOBn), 79.6 (*C*HOR), 79.1 (*C*HCH_2OMs), 71.5 (*C*H_2Ph), 69.0 (*C*H_2OMs), 37.4 (SO_2*Me*), 33.7 (*C*H_2CHOBn), *m/z* (ES^+) 593 (M+Na^+, 100); Accurate mass (ES^+): found 593.1473, $C_{26}H_{34}O_{10}S_2Na^+$ (M+Na^+), requires 593.1486; $[\alpha]_D^{20}$ +6.0 (c = 1.25, $CDCl_3$).

(2R,2'R,4S,4'S,5S,5'S)-4,4'-Bis(benzyloxy)-5,5'-dimethyloctahydro-2,2'-bifuran

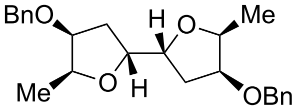

((2*R*,2'*R*,4*S*,4'*S*,5*S*,5'*S*)-4,4'-Bis(benzyloxy)octahydro-2,2'-bifuran-5,5'-iyl)bis(methylene) dimethanesulfonate **14** (200 mg, 0.35 mmol) was dissolved in THF (2 mL), cooled to 0 °C and SuperHydride™ (3.5 mL of a 1.0 M solution in diethyl ether, 3.5 mmol) was added dropwise. The reaction was warmed to room temperature and stirred for 40 h before being quenched with sat. aq. NH_4Cl (5 mL). The mixture was diluted with water until the precipitated salts dissolved (<1 mL). The phases were separated and the aqueous extracted with ethyl acetate (2 × 10 mL), the combined organic phases were dried ($MgSO_4$), filtered and concentrated *in vacuo*. Purification of the residue by flash column chromatography (1:2 ethyl acetate:petrol) gave the product as a colorless oil (85 mg, 0.22 mmol, 64%) along with some material in which only one mesylate had been displaced (34 mg, 71 mmol, 20%). This was resubmitted to the reaction conditions and purified as above (22 mg, 58 mmol, 81%). Overall yield 107 mg, 28 mmol, 80%. R$_f$ 0.58 (1:1 petrol:ethyl acetate); ν_{max}/ cm^{-1} (thin film) 3062, 2979, 2933, 2865 (C-H), 1497, 1451; δ_H (500 MHz $CDCl_3$) 7.35-7.27 (10 H, m Ar*H*), 4.63 (2H, d, *J* = 12.2 Hz, C*H*H'Ar), 4.48 (2H, d, *J* = 12.2 Hz, CH*H'*Ar), 4.14-4.09 (4H, m, C*H*OR, MeC*H*), 3.98 (2H, ddd, *J* = 5.4, 3.8, 1.9 Hz, C*H*OBn), 2.13 (2H, ddd, *J* = 13.2, 4.9, 1.7 Hz C*H*H'CHOBn), 1.97-1.91 (2H, m, CH*H'*CHOBn), 1.30 (6H, d, *J* = 6.4 Hz, *Me*).; δ_C (125 MHz $CDCl_3$) 138.6 (Ar), 128.3 (Ar), 127.4 (Ar), 127.3 (Ar), 80.4 (*C*HOBn), 79.4 (*C*HOR), 78.2 (Me*C*H), 71.1 (*C*H_2Ph), 34.1

(CH_2CHOBn), 14.9 (*Me*); *m/z* (ES^+) 405.2 ($M+Na^+$, 100); Accurate mass (ES^+): found 405.2037, $C_{24}H_{30}O_4Na^+$ ($M+Na^+$), requires; $[\alpha]_D^{20}$ +56.3 (c = 0.65, MeOH).

***(2*R*,2'*R*,4*S*,4'*S*,5*S*,5'*S*)-5,5'-Dimethyloctahydro-2,2'-bifuran-4,4'-diol* 16**

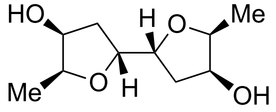

16

(2*R*,2'*R*,4*S*,4'*S*,5*S*,5'*S*)-4,4'-Bis(benzyloxy)-5,5'-dimethyloctahydro-2,2'-bifuran (30 mg, 79 mmol) was dissolved in ethanol (5 mL) and 10% palladium on charcoal (4 mg, 4 mmol Pd) was added. The vessel was evacuated and back-filled with nitrogen three times, then hydrogen three times and the reaction stirred at room temperature under hydrogen atmosphere for 16 h. The atmosphere was exchanged back to nitrogen and the reaction mixture filtered through Celite®, flushing with ethyl acetate. The solvent was removed *in vacuo* to give the product **16** as a colorless oil (15 mg, 74 mmol, 94%). R_f 0.03 (1:1 petrol:ethyl acetate); ν_{max}/ cm^{-1} 3414 (br, O-H), 2973, 2930, 2869(C-H); δ_H (500 MHz $CDCl_3$) 4.20-4.16 (2H, m, C*H*OH), 4.14-4.08 (2H, m, C*H*OR), 4.01 (2H, qd, *J* = 6.4, 2.8 Hz, MeC*H*), 2.01-1.93 (4H, m, CH_2), 1.80 (2H, br s, O*H*), 1.24 (6H, d, *J* = 6.4 Hz, *Me*); δ_C (125 MHz $CDCl_3$) 79.4 (*C*HOR), 78.4 (Me*C*H), 74.1 (*C*HOH), 37.7 (*C*H_2), 14.2 (*Me*); *m/z* (ES^+) 427 ($M+Na^+$, 100); Accurate mass (ES^+): found 225.1095, $C_{10}H_{18}O_4Na^+$ ($M+Na^+$), requires 225.1097; $[\alpha]_D^{20}$ +28.6 (c = 0.75 in MeOH).

(1*S*,3*R*,4*S*,6*S*)-1,6-Bis(benzyloxy)-1,6-di((*R*)-oxiran-2-yl)hexane-3,4-diol 17

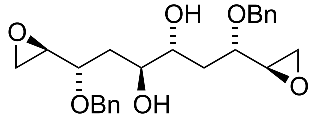

17

(1*S*,6*S*,*Z*)-1,6-Bis(benzyloxy)-1,6-di((*R*)-oxiran-2-yl)hex-3-ene **8** (75 mg, 0.20 mmol) and 4-methylmorpholine-*N*-oxide (46 mg, 0.39 mmol) were dissolved in a mixture of acetone and water (1:1 v/v, 2 mL) and cooled to 0 °C with vigorous stirring. Potassium osmate dihydrate (7 mg, 20 mmol) was added and the reaction stirred for 16 h, warming to room temperature. Sodium sulfite (50 mg) was added and the reaction stirred at room temperature for 30 min. The mixture was partitioned between brine (2 mL) and ethyl acetate (2 mL) and extracted with ethyl acetate (3 × 10 mL). The combined organic phases were dried ($MgSO_4$), filtered and concentrated *in vacuo*. The crude residue was filtered

through a plug of silica, flushing with neat ethyl acetate to give the impure product (80 mg, 0.19 mmol, ~97%). It is possible to crystallise some pure material for characterization purposes from ethyl acetate/hexane; however, generally the crude material was used directly in the next reaction. Data for pure material – white crystalline solid, mp 107-108 °C (ethyl acetate/hexane); R_f 0.25 (ethyl acetate); ν_{max}/ cm^{-1} (thin film) ; δ_H (400 MHz $CDCl_3$) 7.38-7.28 (10H, m, Ar*H*), 4.76 (1H, d, *J* = 11.3 Hz, C*H*H'Ar), 4.72 (1H, d, *J* = 11.4 Hz, C*H*H'Ar), 4.53 (1H, d, *J* = 11.4 Hz, CH*H'*Ar), 4.53 (1H, d, *J* = 11.4 Hz, CH*H'*Ar), [3.83 (1H, tt, *J* = 8.5, 4.4 Hz), 3.79-3.74 (1H, m CHOH), 3.75 (1H, ddd, *J* = 8.0, 4.8, 4.0 Hz), 3.53 (1H, ddd, *J* = 9.4, 5.7, 3.7 Hz)] (C*H*OBn, C*H*OH), 3.58 (1H, d, *J* = 1.4 Hz, O*H*), [3.00 (1H, ddd, *J* = 4.9, 3.8, 2.6 Hz), 2.97 (1H, ddd, *J* = 5.7, 3.8, 2.6 Hz)] (2 × C*H*OCH$_2$), [2.84 (1H, dd, *J* = 5.1, 3.8 Hz), 2.81 (1H, dd, *J* = 5.3, 3.8 Hz)] (2 × CHOC*H*H'), [2.77 (1H, dd, *J* = 5.1, 2.6 Hz), 2.76 (1H, dd, *J* = 5.3, 2.6 Hz)] (2 × CHOCH*H'*), 2.72 (1H, d, *J* = 4.4 Hz, O*H*), 1.95 (1H, ddd, *J* = 14.7, 3.6, 2.1 Hz, *CH*H), 1.84-1.72 (3H, m, *CH*H' + 2 × CH*H'*); δ_C (62.5 MHz $CDCl_3$) 138.1 (Ar), 137.3 (Ar), 128.6 (Ar), 128.4 (Ar), 128.1 (Ar), 127.9 (Ar), 127.9 (Ar), 127.8 (Ar), [78.7, 75.3, 73.8, 72.9] (*C*HOBn, *C*HOH), [72.5, 70.8] (*C*H$_2$Ar), [53.4, 53.0] (*C*HOCH$_2$), [45.9, 45.6] (CHO*C*H$_2$), [34.3, 34.0] (*C*H$_2$); *m/z* (ES$^+$) 437 (M+Na$^+$, 100); Accurate mass (ES$^+$): found 437.1935, $C_{24}H_{30}O_6Na^+$ (M+Na$^+$), requires 437.1935; $[\alpha]_D^{20}$ -42.0 (c = 0.61 in CH_2Cl_2).

*((2*R*,2'*S*,4*S*,4'*S*,5*S*,5'*S*)-4,4'-Bis(benzyloxy)octahydro-2,2'-bifuran-5,5'-diyl)dimethanol*

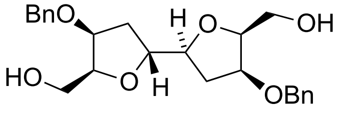

The product of the previous reaction (70 mg, 0.17 mmol) was dissolved in $CDCl_3$ (4 mL) and a small amount of Amberlyst-15® resin added. The reaction was stirred at room temperature for 25 min before being filtered, concentrated *in vacuo* and purified by flash column chromatography (0-5% methanol in ethyl acetate) to give the cyclised diol as a colorless oil (51 mg, 0.12 mmol, 72%). R_f 0.19 (ethyl acetate), ν_{max}/ cm^{-1} (thin film) 3443 (br, O-H), 3060, 3031, 2928, 2864 (C-H), 1497, 1453; δ_H (400 MHz $CDCl_3$) 7.44-7.33 (10H, m, Ar*H*), [4.69 (1H, d, *J* = 11.9 Hz), 4.68 (1H, d, *J* = 11.8 Hz)] (C*H*H'Ar), [4.48 (1H, d, *J* = 11.9 Hz), 4.47 (1H, d, *J* = 11.8 Hz)] (CH*H'*Ar), 4.35-4.25 (3H, m), 4.12 (1H, dd, *J* = 4.5, 9.5 Hz), 3.99-3.94 (2H, m), 3.90-3.81 (4H, m, 2 × C*H$_2$*OH), 2.73-2.34 (2H, br, O*H*), [2.29 (1H, dt, *J* = 13.4, 6.7 Hz,), 2.26 (1H, ddd, *J* = 13.4, 6.4, 2.6 Hz), 2.06 (1H, ddd, *J* = 13.4, 8.2, 5.6 Hz,), 1.96 (1H, ddd, *J* = 13.4, 7.9, 5.0 Hz,)] (C*H$_2$*CHOBn); δ_C (100 MHz $CDCl_3$) 137.6 (Ar), 137.5

(Ar), 128.5 (Ar), 127.9 (Ar), 127.9 (Ar), 127.6 (Ar), 127.5 (Ar), [81.3, 80.9, 80.4, 80.0, 79.7, 79.6] (*C*HORCH$_2$*C*HOBn*C*H), [71.5, 71.5] (*C*H$_2$Ar), [62.1, 62.1] (*C*H$_2$OH), [34.6, 33.6] (*C*H$_2$CHOBn); *m/z* (ES$^+$) 437 (M+Na$^+$, 100); Accurate mass (ES$^+$): found 437.1935, $C_{24}H_{30}O_6Na^+$ (M+Na$^+$), requires 437.1935; $[\alpha]_D^{20}$ +66.6 (*c* = 0.3 in CH_2Cl_2).

*((2*R*,2'*S*,4*S*,4'*S*,5*S*,5'*S*)-4,4'-Bis(benzyloxy)octahydro-2,2'-bifuran-5,5'-diyl)bis(methylene) dimethanesulfonate*

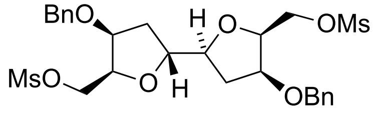

((2*R*,2'*S*,4*S*,4'*S*,5*S*,5'*S*)-4,4'-Bis(benzyloxy)octahydro-2,2'-bifuran-5,5'-diyl)dimethanol (44 mg, 0.11 mmol) was dissolved in CH_2Cl_2 (3 mL), cooled to 0 °C and triethylamine (161 mg, 221 mL, 1.59 mmol) added, followed by dropwise addition of methanesulfonyl chloride (122 mg, 82 mL, 1.06 mmol). The stirred reaction mixture was allowed to warm to room temperature over 16 h before being quenched with aq. HCl (2 M, 5 mL) and extracted with CH_2Cl_2 (2 × 10 mL). The combined organics were dried ($MgSO_4$), filtered and concentrated *in vacuo*. Purification by flash column chromatography (15:1→4:1 CH_2Cl_2:ethyl acetate) gave the product as colorless oil (57 mg, 0.10 mmol, 81%). R_f 0.20 (15:1 CH_2Cl_2:ethyl acetate), v_{max}/ cm^{-1} (thin film) 2923, 2853 (C-H), 1457 (Ar ring), 1352, 1174 (O-SO_2); δ_H (500 MHz CDCl$_3$) 7.44-7.32 (10H, m, Ar*H*), [4.66 (1H, d, *J* = 11.9 Hz), 4.64 (1H, d, *J* = 11.7 Hz)] (C*H*H'Ar), 4.46-4.36 (6H, m), 4.32-4.26 (2H, m), 4.24 (1H, td, *J* = 4.9, 2.2 Hz), 4.20 (1H, ddd, *J* = 6.0, 5.0, 3.5 Hz), 4.13 (1H, ddd, *J* = 7.0, 4.9, 4.0 Hz), 4.00 (1H, dt, *J* = 7.5, 6.3 Hz), 3.00 (3H, s, OSO$_2$*Me*), 2.99 (3H, s, OSO$_2$*Me*), [2.26 (1H, ddd, *J* = 13.4, 6.2, 2.1 Hz), 2.23 (1H, ddd, *J* = 13.7, 7.8, 6.0 Hz)] (C*H*H'CHOBn), [2.00 (1H, ddd, *J* = 13.4, 6.4, 3.4 Hz), 1.96 (1H, ddd, *J* = 13.7, 8.6, 4.9 Hz)] (CH*H'*CHOBn); δ_C (125 MHz CDCl$_3$) 137.6 (Ar), 137.4 (Ar), 128.5 (Ar), 128.5 (Ar), 128.0 (Ar), 127.9 (Ar), 127.7 (Ar), 127.6 (Ar), [80.4, 80.0, 79.5, 79.4, 78.9, 78.4] (*C*H*C*HOBnCH$_2$*C*HOR), [71.4, 71.3] (*C*H$_2$Ar), [69.1, 69.1](*C*H$_2$OMs), 37.4 (2 × SO$_2$*Me*), [34.0, 33.7](*C*H$_2$CHOBn); *m/z* (ES$^+$) 593 (M+Na$^+$, 100); Accurate mass (ES$^+$): found 593.1472, $C_{26}H_{34}O_{10}S_2Na^+$ (M+Na$^+$), requires 593.1486; $[\alpha]_D^{20}$ +21.7 (*c* = 0.4 in CH_2Cl_2)

(2*R*,2'*S*,4*S*,4'*S*,5*S*,5'*S*)-4,4'-Bis(benzyloxy)-5,5'-dimethyloctahydro-2,2'-bifuran

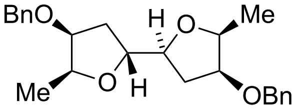

((2*R*,S,4*S*,4'*S*,5*S*,5'*S*)-4,4'-Bis(benzyloxy)octahydro-2,2'-bifuran-5,5'-iyl)bis(methylene) dimethanesulfonate (47 mg, 82 mmol) was dissolved in THF (1 mL), cooled to 0 °C and SuperHydride™ (0.8 mL of a 1.0M solution in diethyl ether, 0.8 mmol) was added dropwise. The reaction was warmed to room temperature and stirred for 16 h before being quenched with sat. aq. NH_4Cl (10 mL). The mixture was diluted with water until the precipitated salts dissolved (<1 mL). The phases were separated and the aqueous extracted with ethyl acetate (3 × 10 mL), the combined organic phases were dried ($MgSO_4$), filtered and concentrated *in vacuo*. Purification of the residue by flash column chromatography (1:3→1:1 ethyl acetate:petrol) gave the product as a colorless oil (16 mg, 42 mmol, 51%) along with some material in which only one mesylate had been displaced (12 mg, 25 mmol, 31%). This was resubmitted to the reaction conditions and purified as above (8 mg, 21 mmol, 84%). Overall yield 24 mg, 63 mmol, 77%. R_f 0.43 (1:1 petrol:ethyl acetate), ν_{max}/ cm^{-1} 3031, 2973, 2929, 2857 (C-H), 1497, 1450 (thin film) ; δ_H (500 MHz $CDCl_3$) 7.36-7.27 (10H, m, Ar*H*), [4.63 (1H, d, *J* = 12.1 Hz), 4.59 (1H, d, *J* = 12.2 Hz)] (C*H*H'Ar), [4.46 (1H, d, *J* = 12.2 Hz), 4.41 (1H, d, *J* = 12.2 Hz)] (CH*H*'Ar), [4.23-4.13 (1H, m), 4.14 (1H, qd, *J* = 6.4, 3.9 Hz), 3.97 (1H, ddd, *J* = 5.2, 4.0. 2.4 Hz), 3.94-3.87 (3H, m)](MeC*H*C*H*OBnCH_2C*H*OR), 2.26-2.21 (2H, m, C*H*H'), [2.07 (1H, ddd, *J* = 13.3, 8.1, 5.2 Hz), 1.89 (1H, ddd, *J* = 13.6, 7.0, 3.0 Hz)(CH*H*'), 1.31 (3H, d, *J* = 6.0 Hz, *Me*), 1.33 (3H, d, *J* = 6.4 Hz, *Me*); δ_C (125 MHz $CDCl_3$) 138.6 (Ar), 138.5 (Ar), 128.3 (Ar), 128.3 (Ar), 127.5 (Ar), 127.4 (Ar), 127.4 (Ar), 127.4 (Ar), [80.2, 79.7, 79.5, 79.5, 78.4, 77.9] (Me*C*H*C*HOBnCH_2*C*HOR), [71.1, 70.9] ($\mathit{C}H_2$Ar), [35.2, 33.5] ($\mathit{C}H_2$), [14.8, 14.7] (*Me*); *m/z* (ES^+) 405 ($M+Na^+$, 100); Accurate mass (ES^+): found 405.2036, $C_{24}H_{30}O_4Na^+$ ($M+Na^+$), requires 405.2036; $[\alpha]_D^{20}$ +53.0 (*c* = 0.2 in CH_2Cl_2).

***(2*R*,2'*S*,4*S*,4'*S*,5*S*,5'*S*)-5,5'-Dimethyloctahydro-2,2'-bifuran-4,4'-diol* 18**

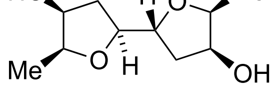

18

(2*R*,2'*S*,4*S*,4'*S*,5*S*,5'*S*)-4,4'-Bis(benzyloxy)-5,5'-dimethyloctahydro-2,2'-bifuran (16 mg, 42 mmol) was dissolved in ethanol (3 mL) and 10% palladium on charcoal (2 mg, 2 mmol Pd) was added. The vessel was evacuated and back-filled with nitrogen three times, then hydrogen three times and the reaction stirred at room temperature under hydrogen atmosphere for 16 h. The atmosphere was exchanged back to nitrogen and the reaction mixture filtered through Celite®, flushing with ethyl acetate. The solvent was removed *in vacuo* to give the product **18** as a colorless oil (8 mg, 40 mmol, 94%). R_f 0.13 (ethyl acetate), ν_{max}/ cm^{-1} (thin film) 3363 (O-H), 3054, 2981, 2930, 2856 (C-H); δ_H (500 MHz $CDCl_3$) 4.48 (1H, ddd, *J* = 11.1, 6.1, 1.8 Hz, C*H*OR), 4.25 (1H, t, *J* = 3.7 Hz, C*H*OH), 4.13-4.09 (2H, m, C*H*OR, MeC*H*), 3.99-3.93 (2H, br m), 3.81 (1H, qd, *J* = 6.2, 2.3 Hz, C*H*Me), [2.25 (1H, ddd, *J* = 14.1, 9.9, 4.9 Hz), 2.07 (1H, ddd, *J* = 13.4, 6.1, 1.1 Hz)] (C*H*H'), [1.77 (1H, dd, *J* = 14.0, 3.0 Hz), 1.71 (1H, ddd, *J* = 13.4, 11.1, 4.4 Hz)] (CH*H'*), 1.29 (3H, d, *J* = 6.2 Hz, *Me*), 1.28 (3H, d, *J* = 6.4 Hz, *Me*); δ_C (125 MHz $CDCl_3$) [79.9, 79.7, 79.3, 78.8] (*C*HOR, Me*C*H), [73.5, 72.0] (*C*HOH), [38.1, 34.9] (*C*H_2) [14.4, 14.1] (*Me*); *m/z* (ES^+) 225 ($M+Na^+$, 100); Accurate mass (ES^+): found 225.1099, $C_{10}H_{18}O_4Na^+$ ($M+Na^+$), requires 225.1097; $[\alpha]_D^{20}$ +31.3 (*c* = 0.15 in CH_2Cl_2).

***(2S,2'R,4S,4'S,5S,5'R)-5-allyl-5'-ethyloctahydro-[2,2'-bifuran]-4,4'-diol* 21**

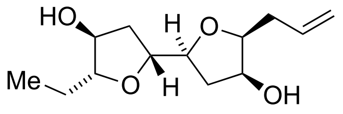

21

To a stirring solution of (2*S*,2'*R*,4*S*,4'*S*,5*S*,5'*R*)-5-allyl-4-(4-bromophenoxy)-5'-ethyl-4'-((4-methoxybenzyl)oxy)octahydro-2,2'-bifuran **20**[6] (8 mg, 18 µmol) in CH_2Cl_2 (1 mL) was added BCl_3 (110 µL, 1M in CH_2Cl_2, 110 µmol) and stirred for 30 min. The reaction was diluted with CH_2Cl_2 (9 mL) and quenched with sat. aq. $NaHCO_3$ (5 mL). The aqueous layer was then separated and extracted with CH_2Cl_2 (3 × 10 mL). The combined organic layers were dried (Na_2SO_4), filtered and concentrated *in vacuo*. Purification *via* flash column chromatography (1:1 petrol:ethyl acetate) gave the title compound **21** as a colorless oil (4 mg, 16 µmol, 94%). R_f 0.36 (1:1 petrol:ethyl acetate), ν_{max}/ cm^{-1} (thin film) 3410 (br O-H), 2932, 1737, 1054; δ_H (400 MHz $CDCl_3$) 5.87 (1H, ddt, *J* = 17.0, 10.0, 7.0 Hz, C*H*=CH_2), 5.17 (1H, d, *J* = 17.0 Hz, CH=C*H*H'), 5.07 (1H, d, *J* = 10.0 Hz, CH=CH*H'*), 4.42 (1H, ddd, *J* = 10.3, 6.0, 0.7 Hz, C*H*ORCHOR), 4.19 (1H, ddd, *J* = 10.3, 5.0, 2.4 Hz, CHORC*H*OR), 4.08 (1H, quin, *J* = 2.6 Hz, CHC*H*OH), 4.01 (1H, dd, *J* = 10.0, 2.6 Hz, C*H*OHCHAllyl), 3.78 (1H, td, *J*=

6.6, 2.6 Hz, EtC*H*), 3.66 (1H, td, *J* = 7.0, 2.6 Hz, CHOHC*H*Allyl), 2.46 (2H, q, *J* = 7.0 Hz, C*H*$_2$CH=CH$_2$), 2.22 (1H, ddd, *J* = 14.1, 10.0, 5.0 Hz, CH*H'*CHOH), 1.82-1.90 (2H, m, CH*H'*CHOH, CHOHC*H*H'), 1.72-1.55 (4H, m, CHOHCH*H'*, CH_3C*H*$_2$), 1.00 (3H, t, *J* = 7.5 Hz, C*H*$_3$), δ_C (125 MHz $CDCl_3$) 134.9 (*C*H=CH$_2$), 116.9 (CH=*C*H$_2$), 88.6 (Et*C*H), 83.6 (CHOH*C*HAllyl), 78.8 (*C*HORCHOR), 78.4 (CHOR*C*HOR), 75.3 (CH*C*HOH), 70.9 (*C*HOHCHAllyl), 37.5 (CHOH*C*H$_2$), 34.2 (*C*H$_2$CHOH), 33.5 (*C*H$_2$CH=CH$_2$), 26.7 (CH_3*C*H$_2$), 10.3 (*C*H$_3$), *m/z* (ES$^+$) 265 (M+Na$^+$, 100), Accurate mass (ES$^+$): found 265.1414, $C_{13}H_{22}O_4Na^+$ (M+Na$^+$), requires 265.1410, $[\alpha]_D^{20}$ +15.9 (*c* = 0.8 in MeOH).

(2S,2'R,4R,4'R,5S,5'R)-5-Allyl-5'-ethyloctahydro-[2,2'-bifuran]-4,4'-diol 22

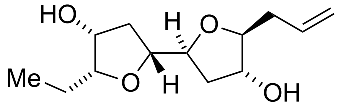

22

To a stirred solution of (2*S*,2'*R*,4*S*,4'*S*,5*S*,5'*R*)-5-allyl-5'-ethyloctahydro-[2,2'-bifuran]-4,4'-diol **22** (4 mg, 17 µmol) in THF (0.2 mL) at 0 °C was added PPh_3 (45 mg, 0.17 mmol), and DIAD (34.3 mg, 33.5 µL, 0.17 mmol). After 30 min 4-nitrobenzoic acid (28.4 mg, 0.17 mmol) was added and the reaction stirred for 2 h at RT. The reaction was then rapidly concentrated *in vacuo* and plugged through silica eluting with CH_2Cl_2. The solvent was then removed *in vacuo***.** The crude was then dissolved in MeOH (1 mL), and K_2CO_3 (7 mg 52 µmol) was added. After stirring for an hour the reaction was diluted with H_2O (5 mL) and EtOAc (10 mL). The aqueous layer was separated and extracted with EtOAc (3 × 10 mL) and the combined organic layers were dried ($MgSO_4$), filtered and concentrated *in vacuo*. Purification *via* flash column chromatography (1:1 petrol:ethyl acetate) gave the title compound as a colorless oil (3 mg, 12 µmol, 75%). R_f 0.38 (1:1 petrol:ethyl acetate); ν_{max}/ cm^{-1} (thin film) 3390 (br O-H), 2930, 1437, 1069; δ_H (500 MHz $CDCl_3$) 5.84 (1H, ddt, *J* = 14.0, 10.0, 7.0 Hz, C*H*=CH$_2$), 5.17 (1H, dq, *J* = 17.0, 1.6 Hz, CH=C*H*H'), 5.14 (1H, *J* = 10.0, 1.6 Hz, CH=CH*H'*), 4.41 (1H, ddd, *J* = 10.2, 6.2, 1.7 Hz, CHORC*H*OR), 4.18 (1H, dt, *J* = 10.2, 2.3 Hz, C*H*ORCHOR), 4.13 (1H, dt, *J* = 6.6, 3.8 Hz, C*H*OHCH$_2$), 4.01 (1H, br m, CH_2C*H*OH), 3.91 (1H, td, *J* = 6.6, 3.8 Hz, C*H*Allyl), 3.54 (2H, td, *J* = 7.0, 2.3 Hz, EtC*H*OR, CHO*H*), 2.45-2.29 (2H, m, C*H*$_2$CH=CH$_2$), 2.33 (1H, ddd, *J* = 14.0, 10.2, 5.3 Hz, CHOHC*H*H'), 1.88 (1H, ddd, *J* = 13.5, 6.2, 3.2 Hz, C*H*H'CHOH), 1.84 (1H, dd, *J* = 14.0, 2.3 Hz, CHOHCH*H'*), 1.76-1.65 (3H, m, CH_3C*H*$_2$, CH*H'*CHOH), 0.98 (3H, t, *J*= 7.5 Hz, C*H*$_3$); δ_C (125 MHz $CDCl_3$) 133.4 (*C*H=CH$_2$), 118.2 (CH=*C*H$_2$), 85.9 (CHOH*C*HAllyl), 85.7 (Et*C*HOR), 78.9

(CHOR*C*HOR), 78.2 (*C*HORCHOR), 75.0 (*C*HOHCH$_2$), 70.7 (CH$_2$*C*HOH), 37.9 (*C*H$_2$CH=CH$_2$), 37.2 (*C*H$_2$CHOH), 34.4 (*C*H$_2$CHOH), 21.8 (CH$_3$*C*H$_2$), 10.5 (*C*H$_3$); *m/z* (ES$^+$) 265 (M+Na$^+$, 100); Accurate mass (ES$^+$): found 265.1411, $C_{13}H_{22}O_4Na^+$ (M+Na$^+$), requires 265.1410; $[\alpha]_D^{20}$ -7.0 (*c* = 0.2 in MeOH).

***(2R,2'S,4R,4'R,5R,5'S)-5-Ethyl-5'-((E)-pent-2-en-4-yn-1-yl)octahydro-[2,2'-bifuran]-4,4'-diol*, Laurefurenyne B 5b**

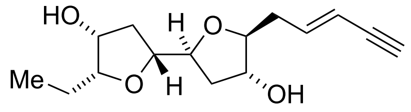

5b; laurefurenyne B

Following the procedure of Kim,[7] to a stirred solution of (2*S*,2'*R*,4*R*,4'*R*,5*S*,5'*R*)-5-allyl-5'-ethyloctahydro-[2,2'-bifuran]-4,4'-diol **22** (3 mg, 12 µmol) in degassed CH_2Cl_2 was added crotonaldehyde (8.4 mg, 10 µL, 0.12 mmol) followed by Grubb's 2nd generation catalyst **19** (1 mg, 1.2 µmol). The reaction was stirred at 40 °C for 1 h before being quenched by the addition of DMSO (0.1 mL) and left to stir at RT for 16 h. The reaction was then concentrated *in vacuo* and purified *via* rapid flash column chromatography (ethyl acetate). The aldehyde was then immediately added as a solution in THF (0.5 mL + 0.5 mL wash) to a stirring solution of TMSCHN$_2$Li at -78 °C (prepared by adding BuLi (75 µL of 1.6 M solution in hexane, 0.12 mmol) to a stirred solution of TMSCH$_2$N$_2$ (0.12 mL 1 M solution in Et$_2$O, 0.12 mmol) in THF (0.5 mL) at -78 °C and stirring for 15 min). The reaction was stirred for 1 h at -78 °C then warmed to 0 °C and stirred for 1 h. The reaction was quenched with 2 M aq. HCl (5 mL) and extracted with EtOAc (3 × 10 mL). The combined organic layers were washed with sat. aq. NaHCO$_3$ (5 mL), and the aqueous layer extracted with EtOAc (10 mL). The combined organic layers were then dried (MgSO$_4$), filtered and concentrated. Purification *via* flash column chromatography (1:1 petrol:ethyl acetate) gave the title compound **5b** as a colorless oil (1.5 mg, 5.5 µmol, 45%), R$_f$ 0.42 (ethyl acetate), ν_{max}/ cm^{-1} (thin film) 3420 (br O-H), 3310, 2920, 1669, 1059; δ_H (500 MHz CDCl$_3$) 6.22 (1H, dt, *J* = 15.5, 7.6 Hz, *C*H=CH), 5.58 (1H, ddd, *J* = 15.5, 2.2, 2.2 Hz, CH=*C*H), 4.41 (1H, ddd, *J* = 10.2, 6.3, 2.5 Hz, CHOR*C*HOR), 4.16 (1H, dt, *J* = 10.2, 2.5 Hz, C*H*ORCHOR), 4.13-4.09 (1H, m, CH$_2$C*H*OH), 4.03 (1H, ddd, *J*= 11.1, 5.2, 2.4 Hz, C*H*OHCH$_2$), 3.90 (1H, td, *J* = 6.5, 3.7 Hz, CHOHAllyl), 3.54 (1H, td, *J* = 7.0, 2.4 Hz, Et*C*HOR), 3.34 (1H, d, *J* = 11.1 Hz, O*H*), 2.84 (1H, d, *J* = 2.2 Hz, CC*H*), 2.34-2.48 (2H, m, C*H*$_2$CH=CH$_2$), 2.23 (1H, ddd, *J* = 14.5, 10.2, 5.2 Hz, CHOHC*H*H'), 1.90 (1H, ddd, *J* = 13.4, 6.3, 2.7 Hz, C*H*H'CHOH), 1.82 (1H, dd, *J* = 14.5,

3.0 Hz, CHOHCH*H*'), 1.75-1.68 (3H, m, CH*H*'CHOH, CH_3CH_2), 0.98 (3H, t, J = 7.5 Hz); δ_C (125 MHz $CDCl_3$) 140.6 (*C*H=CH_2), 112.0 (CH=*C*H_2), 85.7 (Et*C*HOR), 85.5 (CHOH*C*HAllyl), 81.7 (*C*CH), 79.1 (CHOR*C*HOR), 78.1 (*C*HORCHOR), 76.9 (C*C*H), 74.9 (*C*HOHCH$_2$), 70.8 (CH$_2$*C*HOH), 37.3 (*C*H$_2$CHOH), 37.0 (*C*H$_2$CH=CH$_2$), 34.6 (CHOH*C*H$_2$), 21.8 (CH$_3$*C*H$_2$), 10.5 (*C*H$_3$); *m/z* (ES^+) 298 ($M+Na^+$, 100); Accurate mass (ES^+): 289.1411 found, $C_{15}H_{22}O_4Na^+$ ($M+Na^+$), requires 289.1410; $[\alpha]_D^{20}$ -20.0 (c= 0.1 in MeOH). Lit. value[8] $[\alpha]_D^{20}$ -13.0 (c = 0.1 in MeOH).

1.3 Figures and Tables

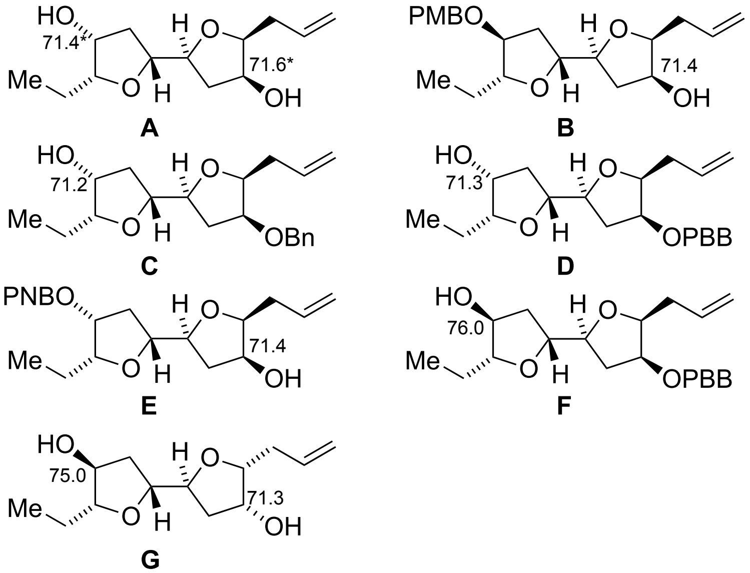

Figure 1. ^{13}C NMR chemical shifts for hydroxy-bearing carbon atoms for seven hydroxy-substituted 2,2'-bifuranyls (**A-G**). Data for compounds **A-F** is in C_6D_6. Data for **G** is in $CDCl_3$. Compounds **A**, **D** and **F** have previously been reported[6]; the C-OH resonance of compound **F** was originally misassigned.[6] Full data for compounds **B**, **C**, **E** and **G** will be reported in due course. *Assignments may be reversed. PMB = 4-methoxybenzyl, Bn = benzyl, PBB = 4-bromobenyzl, PNB = 4-nitrobenzoyl.

Table 1 – Comparison of the ^{13}C NMR data ($CDCl_3$) for laurefurenyne B (5b) with that of the natural product.[8]

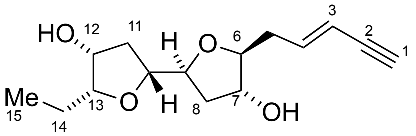

30b

Carbon	δ_C Synthetic laurefurenyne B 30b (125 MHz, $CDCl_3$) (ppm)	δ_C Natural laurefurenyne B 30b (100 MHz, $CDCl_3$) (ppm)	Difference (ppm)
1	76.9	76.7	0.2
2	81.7	81.7	0.0
3	112.0	112.0	0.0
4	140.6	140.7	0.1
5	37.0	37.1	0.1
6	85.5	85.4	0.1
7	74.9	74.9	0.0
8	37.3	37.3	0.0
9	79.1	79.1	0.0
10	78.1	78.1	0.0
11	34.6	34.5	0.1
12	70.8	70.8	0.0
13	85.7	85.7	0.0
14	21.8	21.8	0.0
15	10.5	10.5	0.0

Table 2 – Comparison of the ^{13}C NMR data ($CDCl_3$) for laurefurenyne B (5b) with that of the natural product.[8]

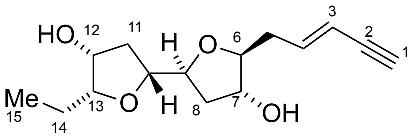

30b

Proton	δ_H Synthetic laurefurenyne B 30b (500 MHz, $CDCl_3$) (ppm)	δ_H Natural laurefurenyne B 30b (400 MHz, $CDCl_3$) (ppm)[a]
1	2.82 (d, 2.2)	2.83 (d, 2.1)
3	5.58 (ddd, 15.5, 2.2, 2.2)	5.58 (dd, 15.5, 2.1)
4	6.22 (dt, 15.5, 7.6)	6.22 (dt, 15.5, 7.6)
5<br>5’	2.43-2.48 (m)<br>2.34-2.42 (m)	2.44 (m)<br>2.37 (m)
6	3.90 (td, 6.5, 3.7)	3.90 (ddd, 10.2, 6.6, 3.7)
7	4.09-4.13 (m)	4.10 (m)
8<br>8’	1.90 (ddd, 13.4, 6.3, 2.7)<br>1.68-1.75 (m)	1.85 (m)<br>1.68 (m)
9	4.41 (ddd, 10.2, 6.3, 2.5)	4.40 (m)
10	4.16 (dt, 10.2, 2.5)	4.16 (m)
11<br>11’	2.23 (ddd, 14.5, 10.2, 5.2)<br>1.82 (dd, 14.5, 3.0)	2.20 (m)<br>1.79 (dd, 13.9, 3.0)
12	4.03 (ddd, 11.1, 5.2, 2.4)	4.01 (dd, 5.5, 2.3)
13	3.54 (td, 7.0, 2.4)	3.52 (ddd, 9.4, 6.9, 2.4)
14 (2H)	1.68-1.75 (m)	1.69 (m)
15 (3H)	0.98 (t, 7.5)	0.97 (t, 7.5)

[a]Data for laurefurenyne quotes chemical shift values for the start point of a multiplet.

1.4 Synthetic References

[1] T. S. Li, J. T. Li, H. Z. Li, *J. Chromatogr. A* **1995**, *715*, 372-375.

[2] The epoxide **6** was prepared by benzylation of the corresponding epoxy alcohol which inturn was prepared in >95% ee by Sharpless asymmetric kinetic resolution of 1,5-hexadien-3-ol according to a modification of that reported by Crimmins, M. T. Crimmins, M. T. Powell, *J. Am. Chem. Soc.* **2003**, *125*, 7592-7595. The benzyl ether **6** and its enantiomer have been reported by a number of other groups see: J. Mulzer, A. Angermann, W. Münch, *Liebigs Ann. Chem.* **1986**, *1986*, 825-838; A. Sharma, S. Gamre, S. Chattopadhyay, *Tetrahedron Lett.* **2007**, *48*, 3705-3707; G. Bhaskar, A. Ravii Kumar, E. Ramu, B. Venkateswara Rao, *Synthesis* **2008**, 3061-3064.

[3] S. H. Hong, D. P. Sanders, C. W. Lee, R. H. Grubbs, *J. Am. Chem. Soc.* **2005**, *127*, 17160-17161.

[4] D. W. Knight, I. R. Morgan, A. J. Proctor, *Tetrahedron Lett.* **2010**, *51*, 638-640.

[5] K. B. Sharpless, W. Amberg, Y. L. Bennani, G. A. Crispino, J. Hartung, K. S. Jeong, H. L. Kwong, K. Morikawa, Z. M. Wang, *J. Org. Chem.* **1992**, *57*, 2768-2771.

[6] B. S. Dyson, J. W. Burton, T. I. Sohn, B. Kim, H. Bae, D. Kim, *J. Am. Chem. Soc.* **2012**, *134*, 11781-11790.

[7] B. Kim, M. Lee, M. J. Kim, H. Lee, S. Kim, D. Kim, M. Koh, S. B. Park, K. J. Shin, *J. Am. Chem. Soc.* **2008**, *130*, 16807-16811.

[8] W. M. Abdel-Mageed, R. Ebel, F. A. Valeriote, M. Jaspars, *Tetrahedron* **2010**, *66*, 2855-2862.

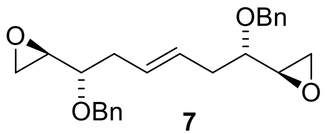

7

^{1}H NMR 400 MHz, $CDCl_3$

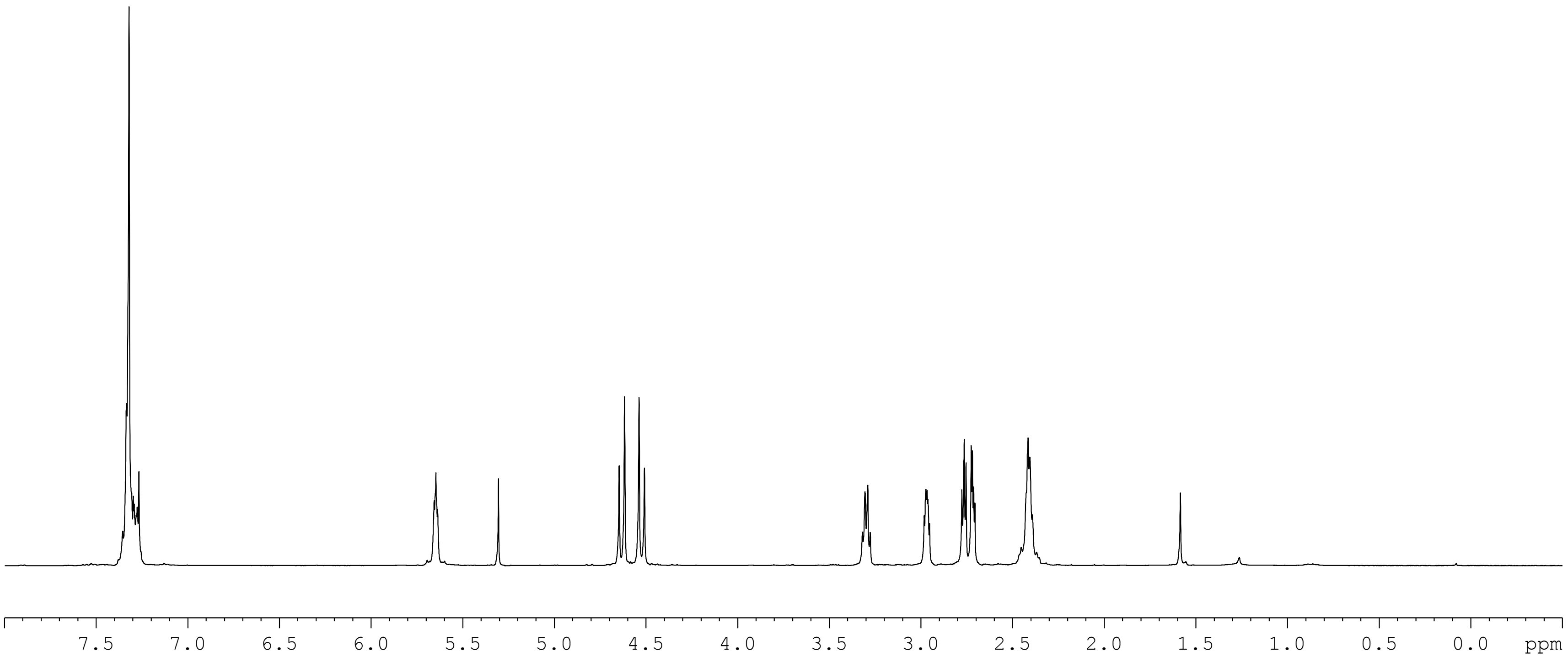

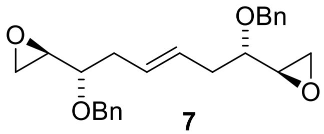

7

^{13}C NMR, 125 MHz, $CDCl_3$

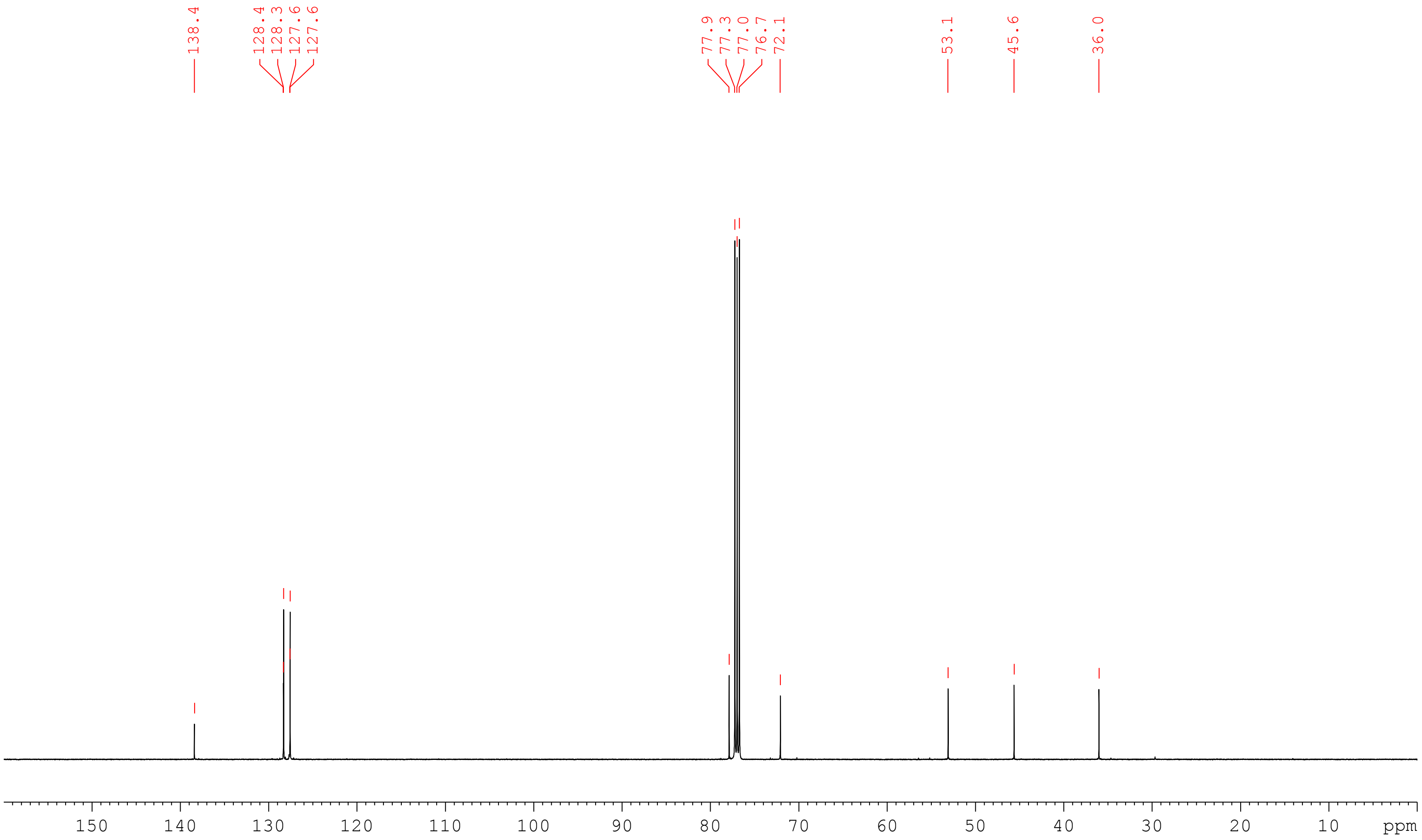

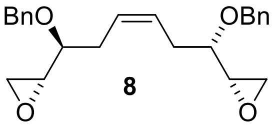

8

^{1}H NMR 400 MHz, $CDCl_3$

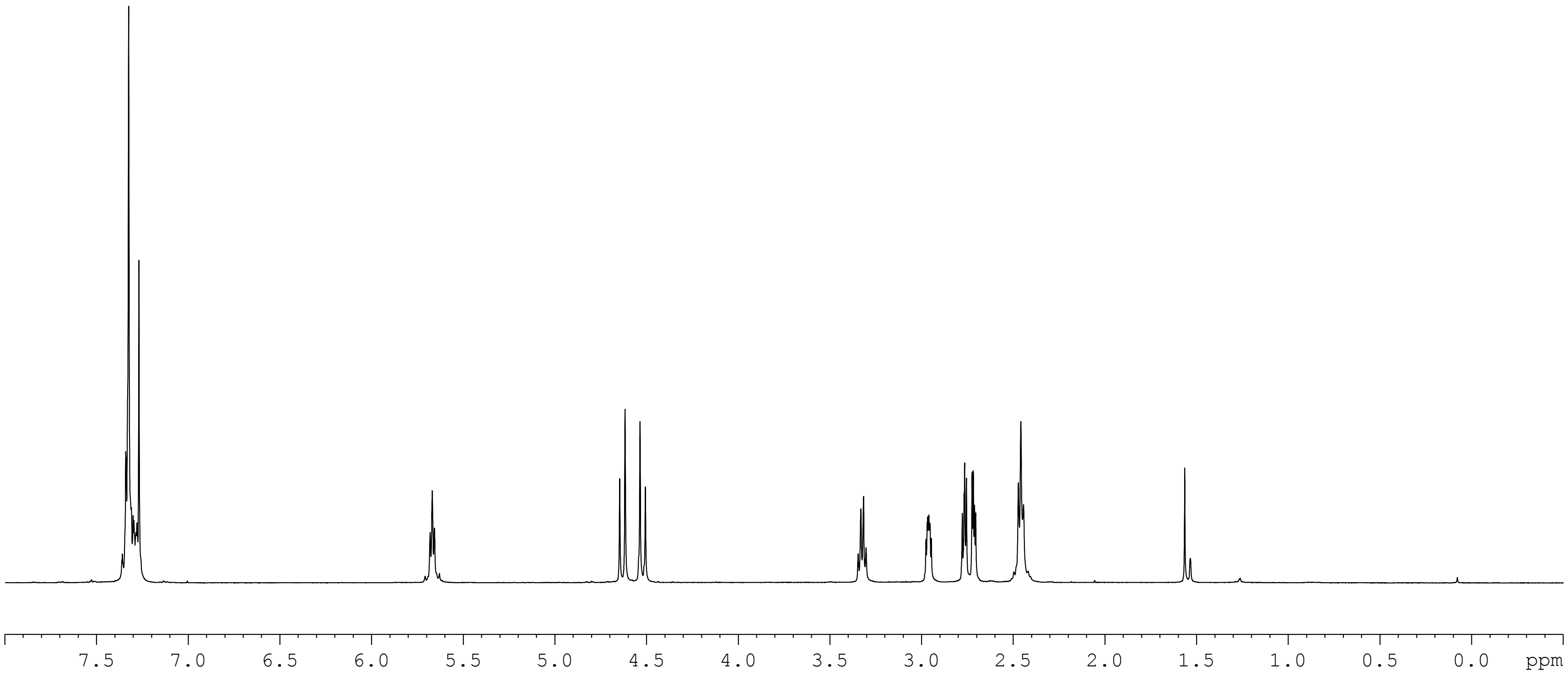

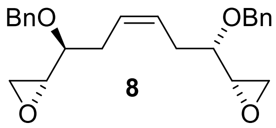

^{13}C NMR, 62.5 MHz, $CDCl_3$

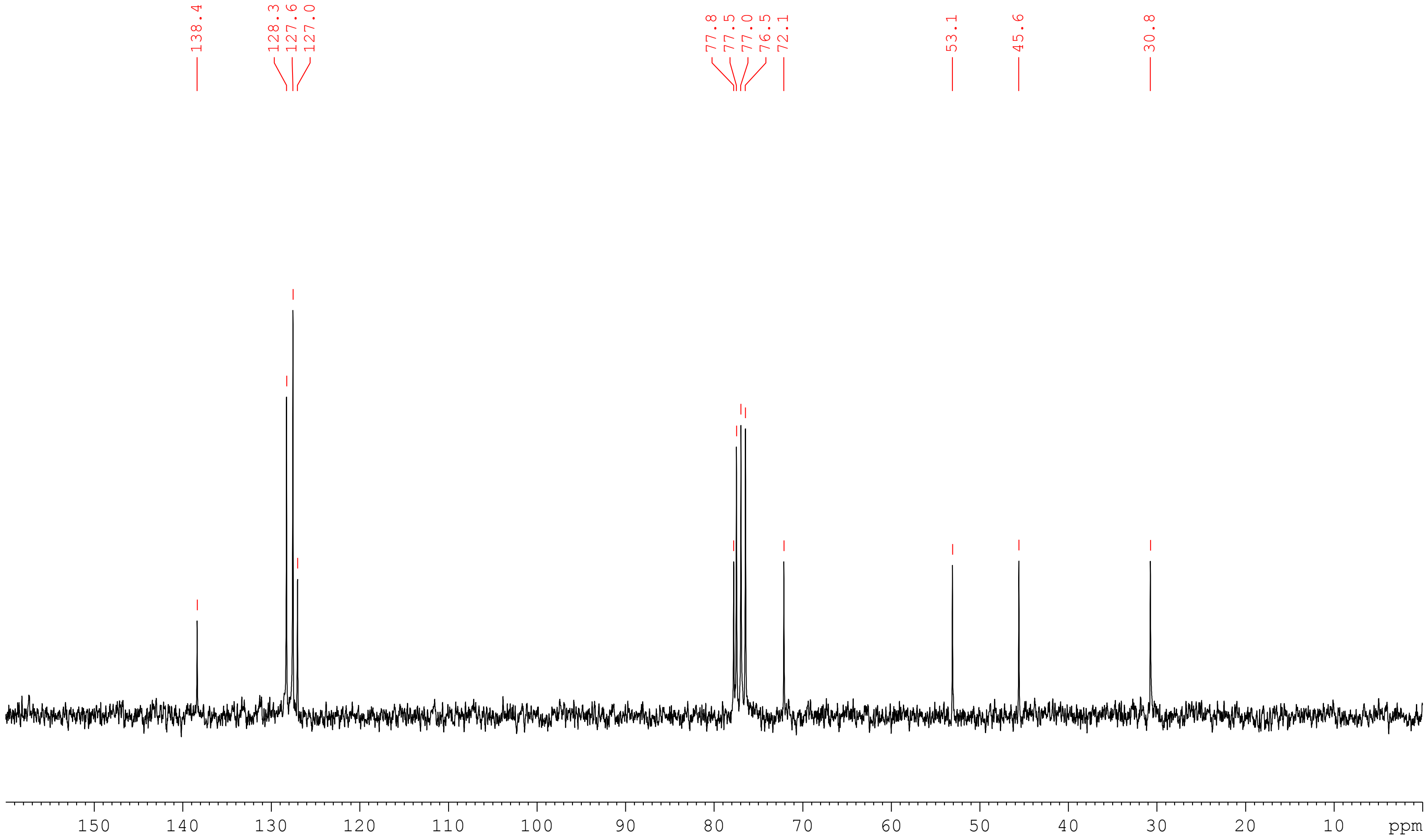

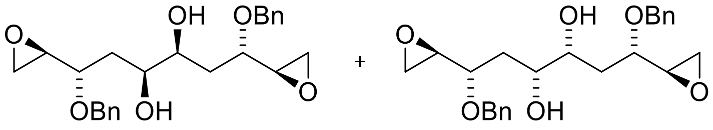

9 **10**

^{1}H NMR 500 MHz, $CDCl_3$ crude product mixture after from reaction of alkene **7** with AD-mix – α; ca. 3.5:1 mixture of **9** and **10**

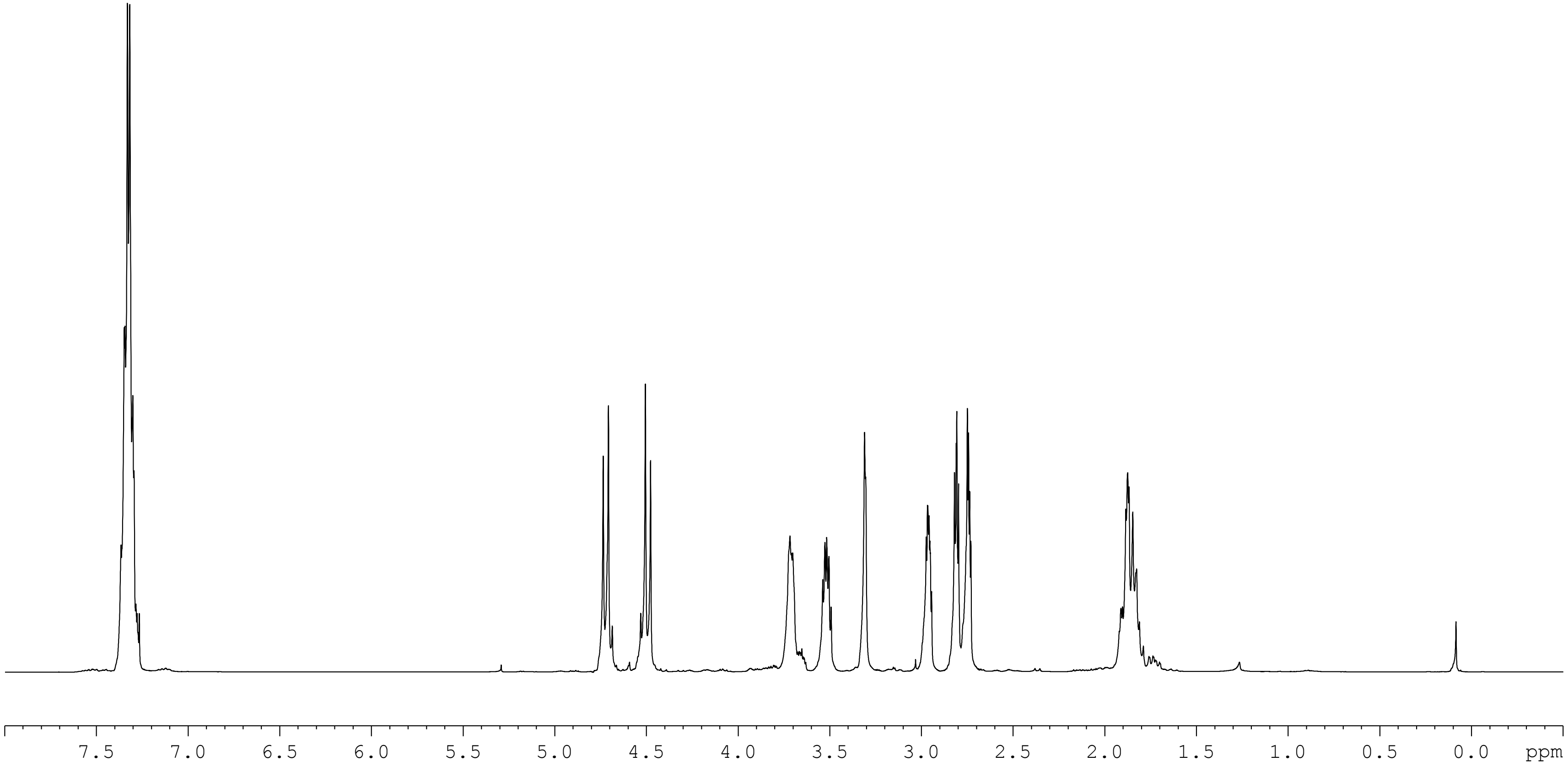

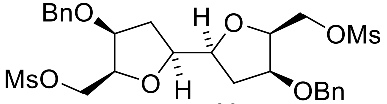

13

^{1}H NMR 400 MHz, $CDCl_3$

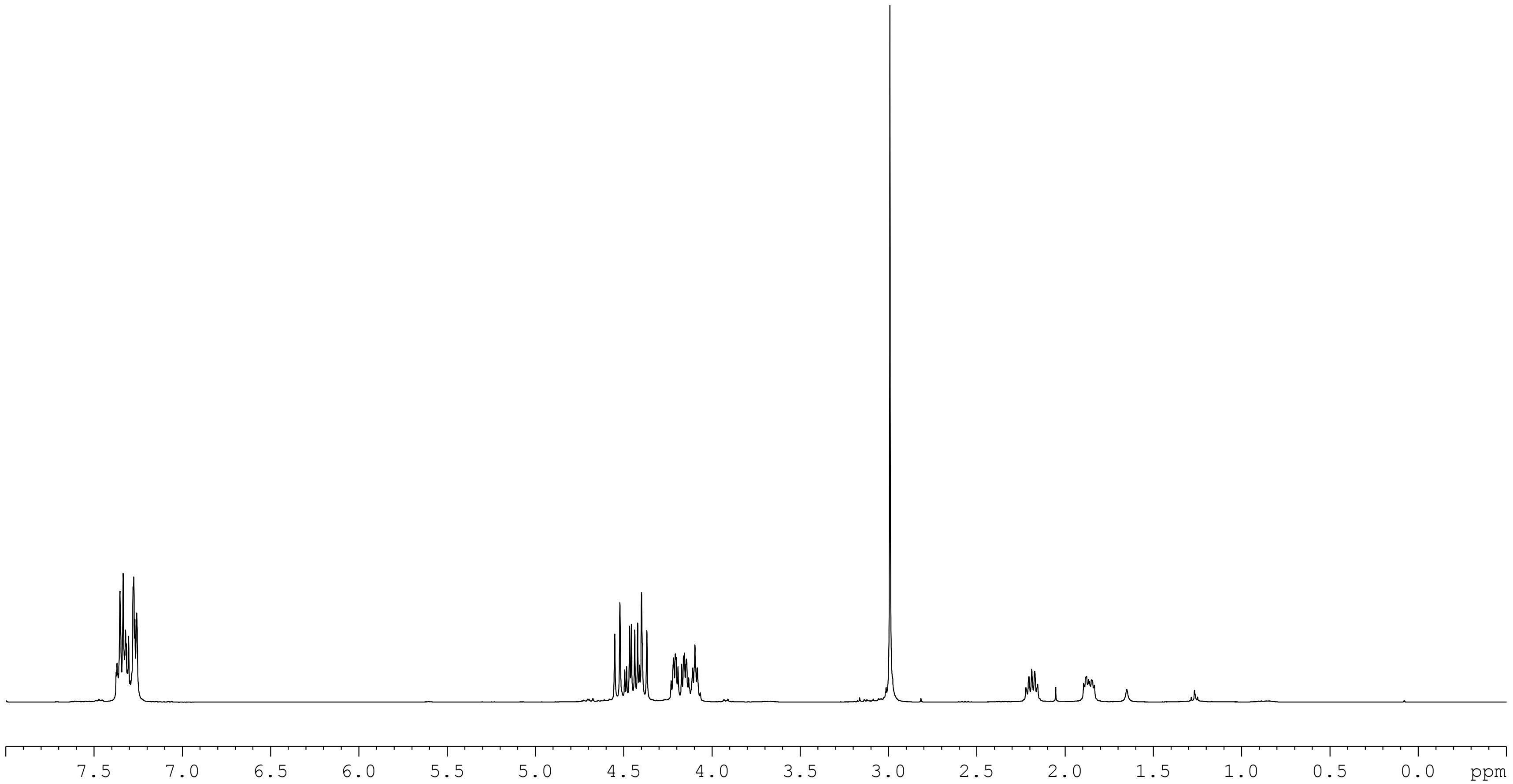

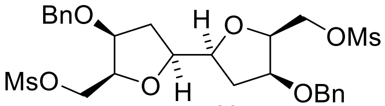

13

^{13}C NMR, 62.5 MHz, $CDCl_3$

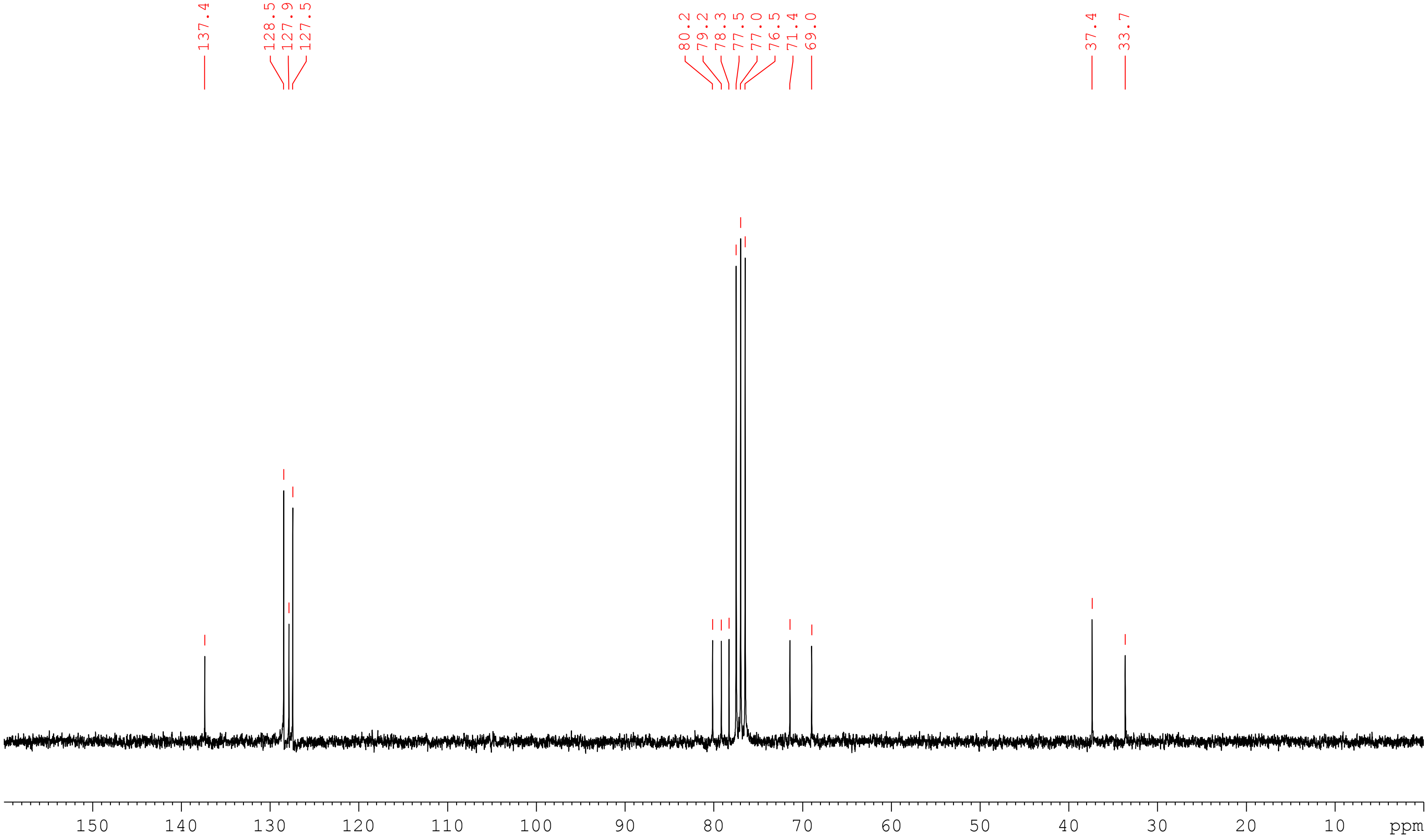

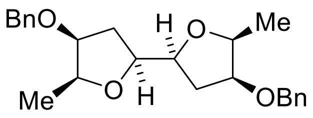

^{1}H NMR 500 MHz, $CDCl_3$

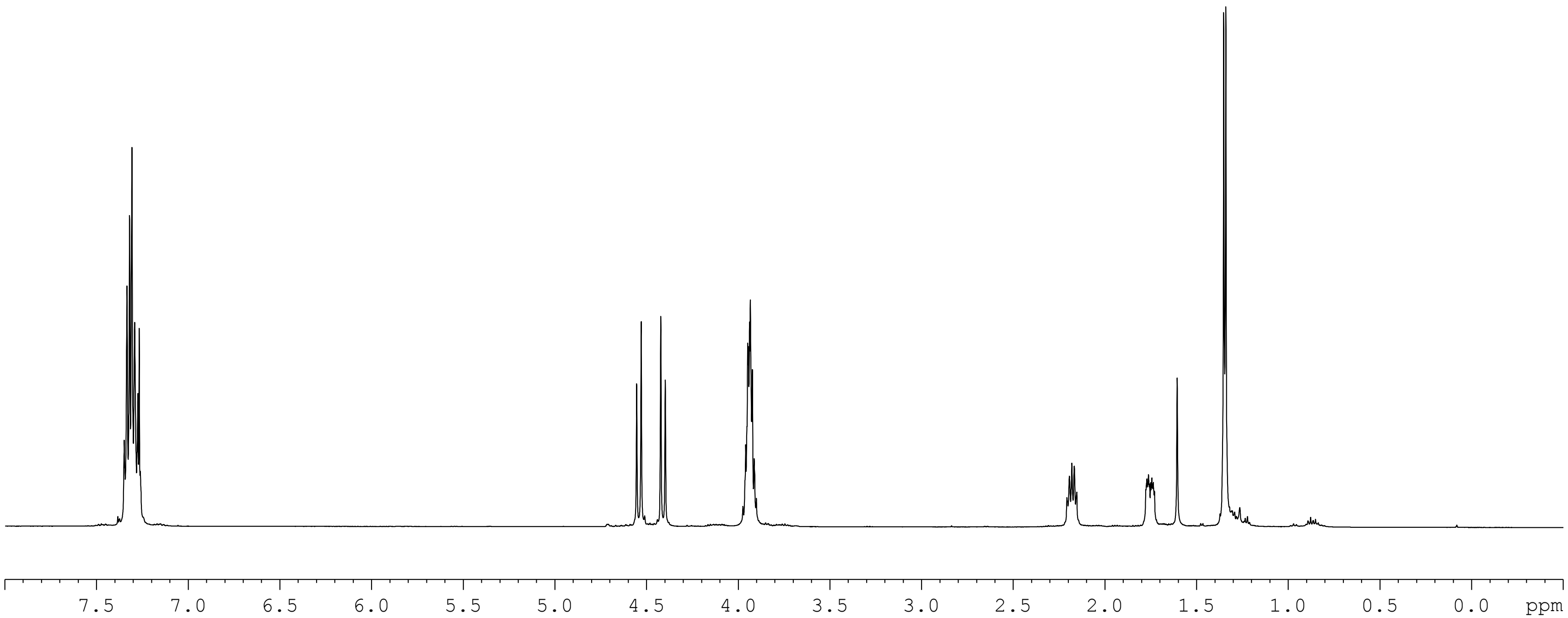

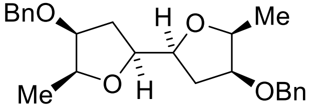

^{13}C NMR (DEPTQ), 125 MHz, $CDCl_3$

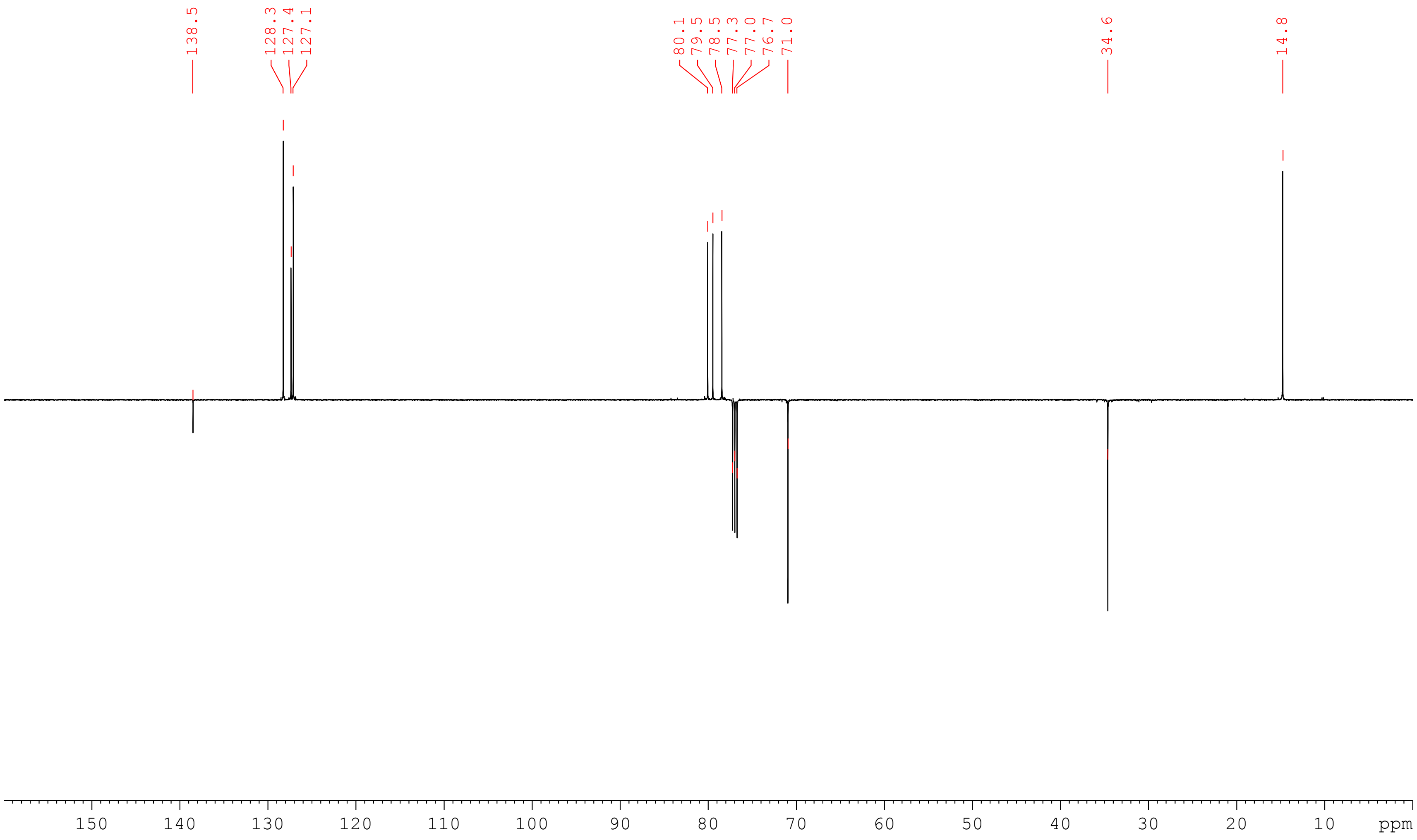

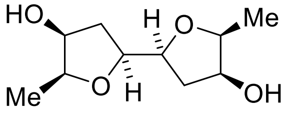

15

^{1}H NMR 500 MHz, $CDCl_3$

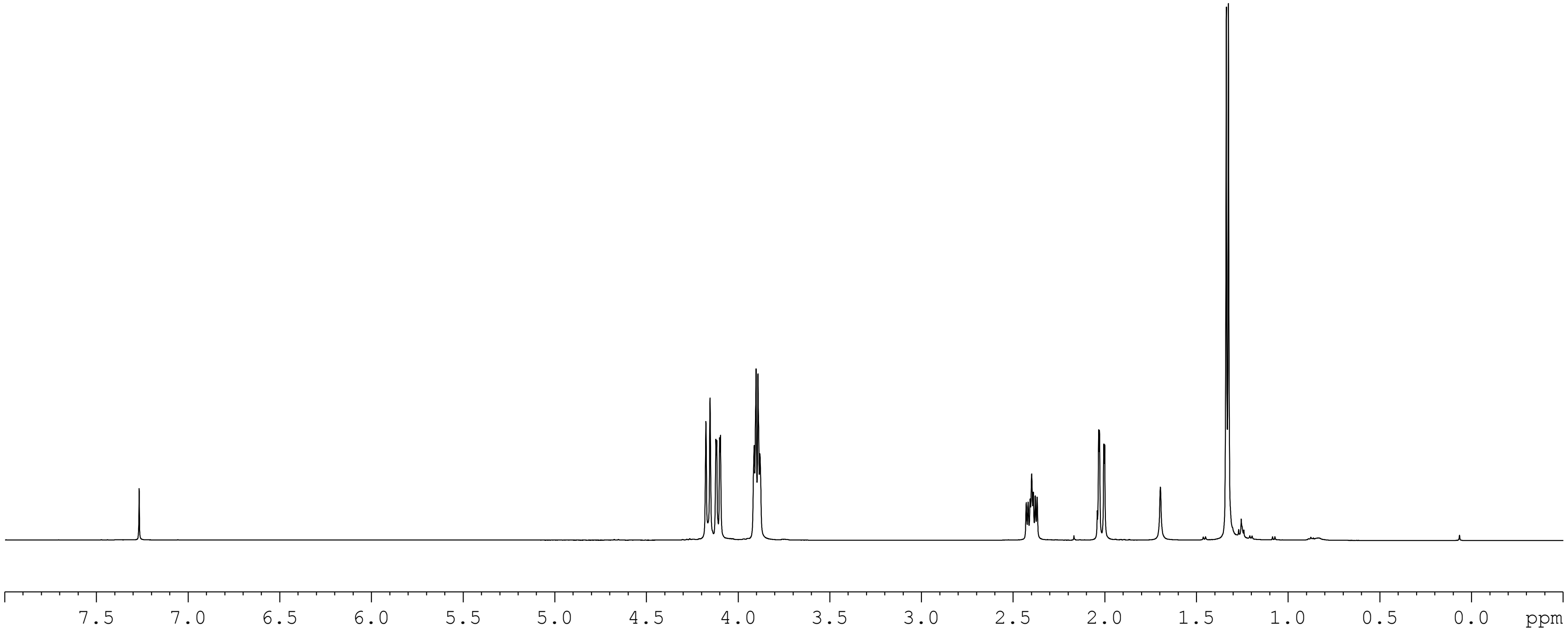

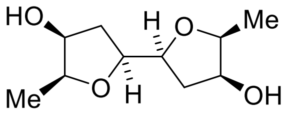

15

^{13}C NMR (DEPT Q), 125 MHz, $CDCl_3$

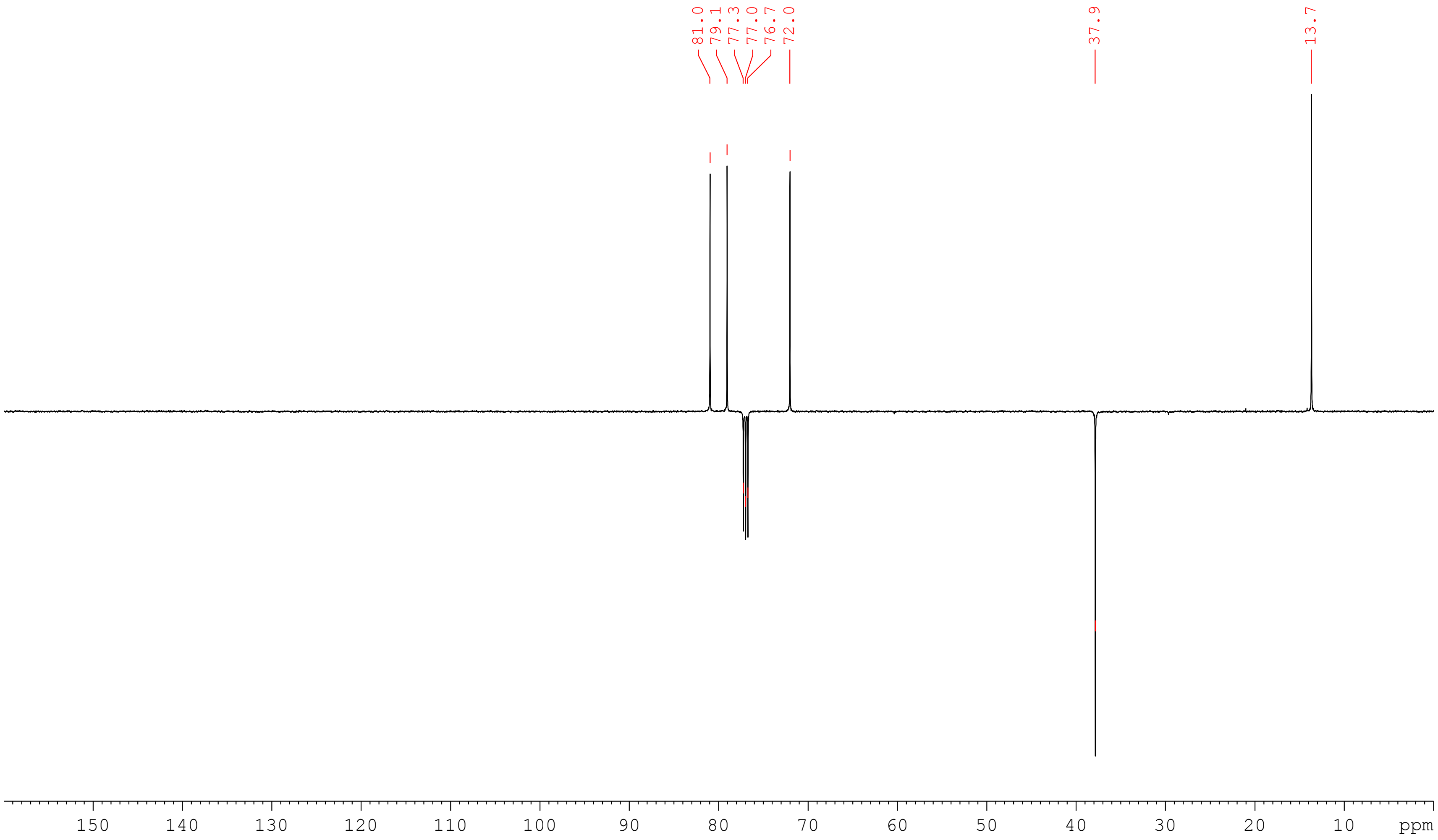

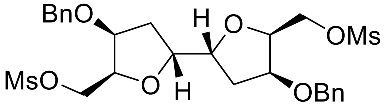

14

^{1}H NMR 500 MHz, $CDCl_3$

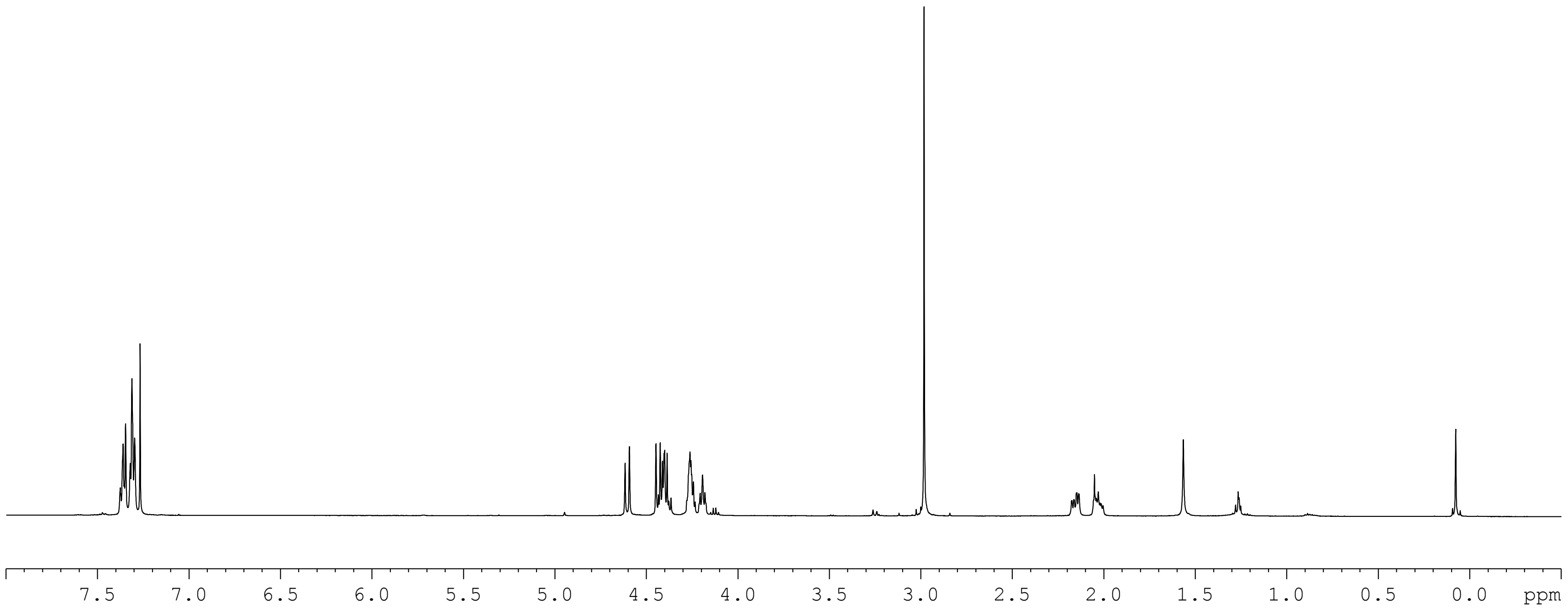

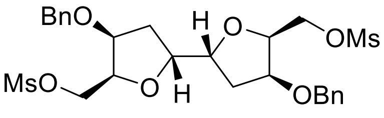

14

^{13}C NMR (DEPT Q), 125 MHz, $CDCl_3$

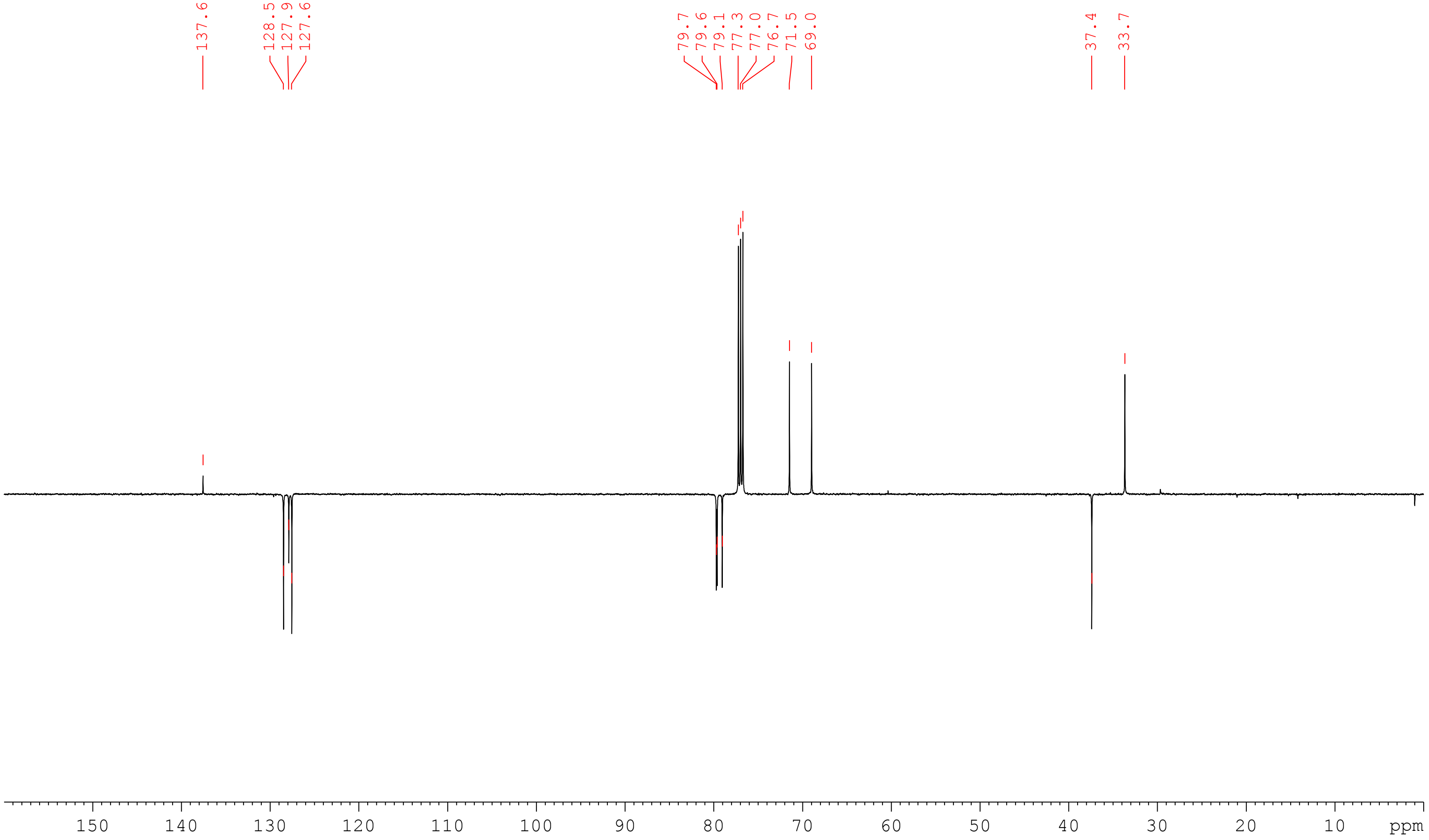

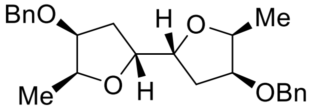

^{1}H NMR 500 MHz, $CDCl_3$

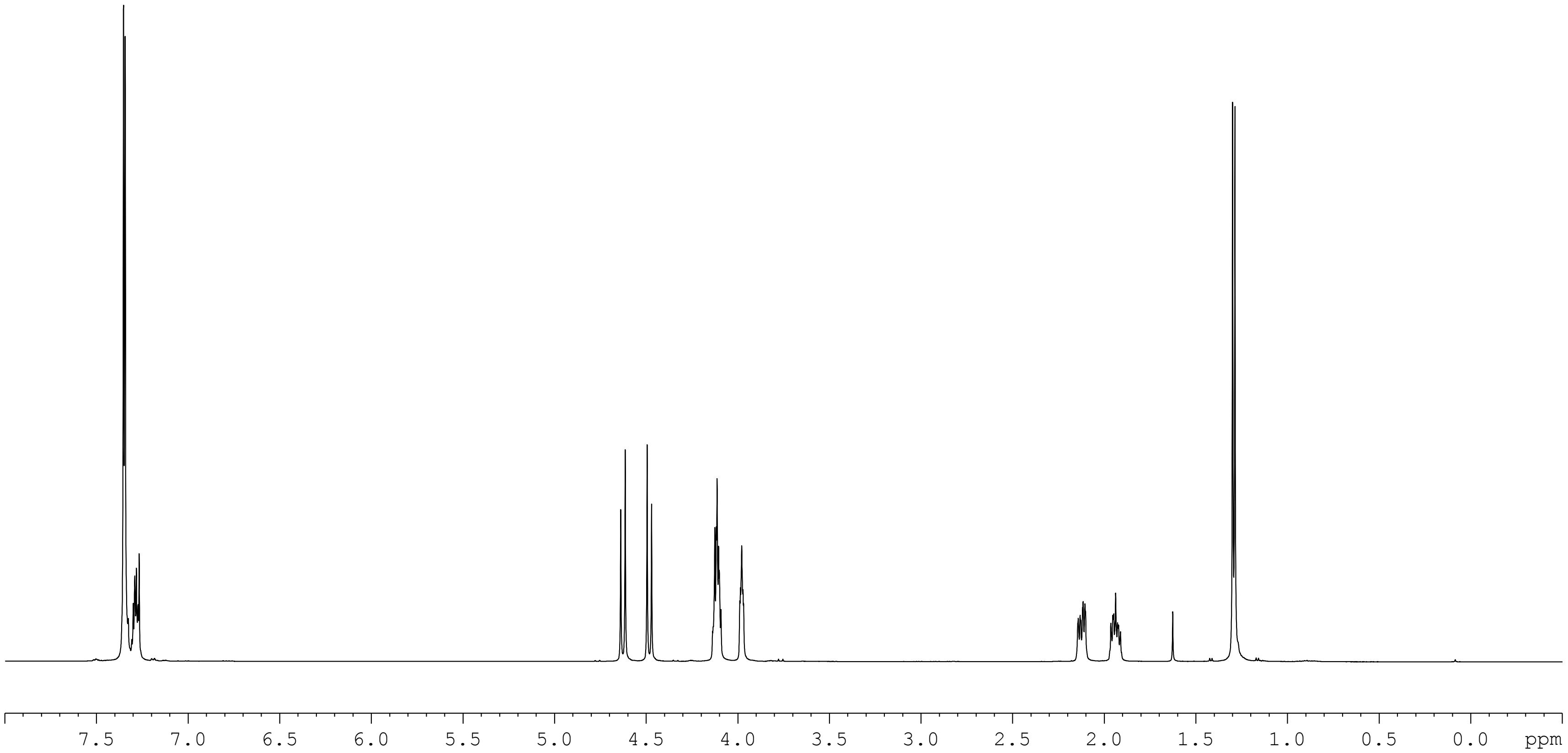

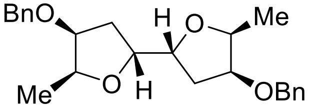

^{13}C NMR (DEPT Q), 125 MHz, $CDCl_3$

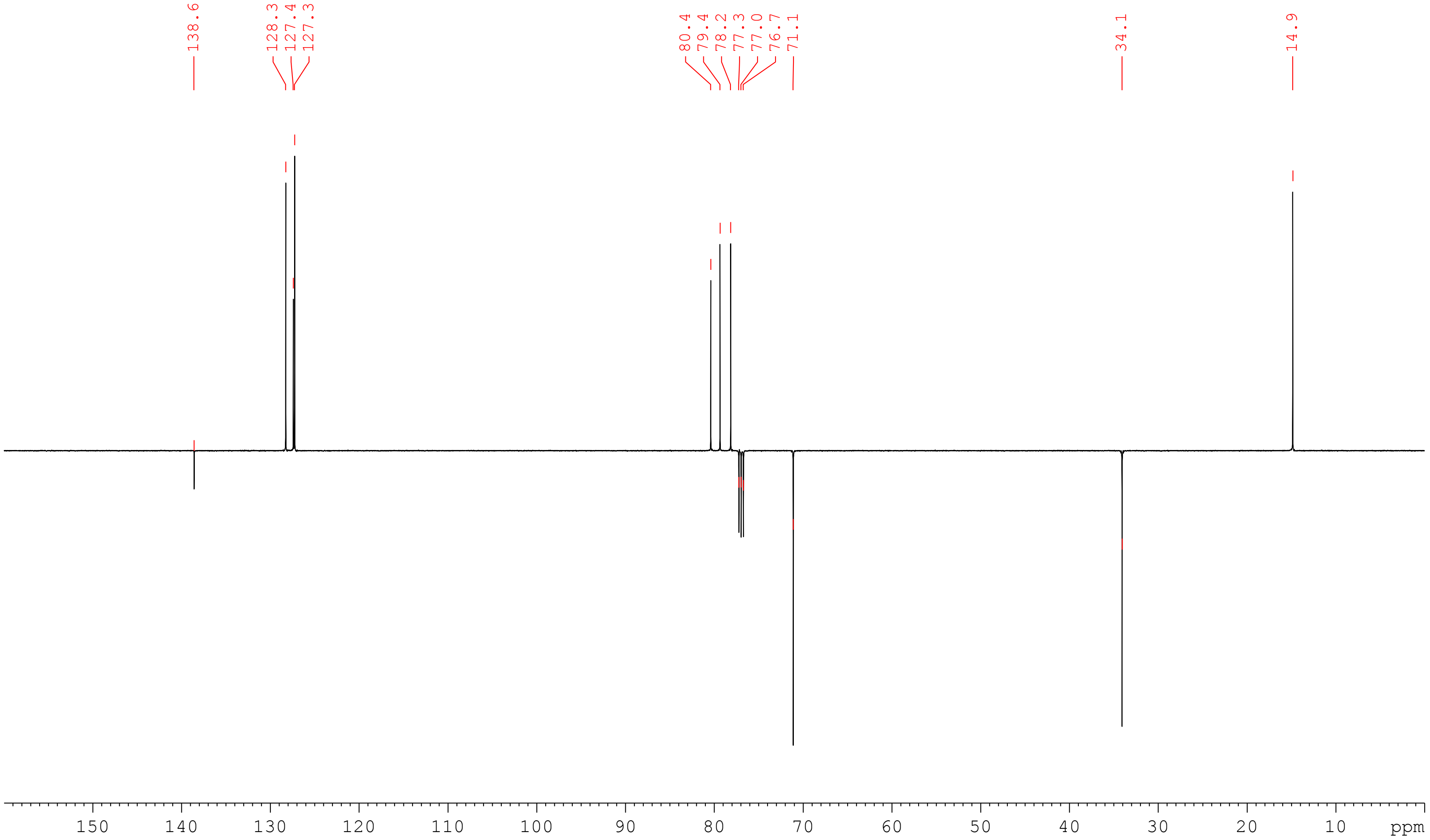

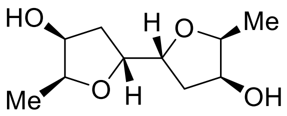

16

^{1}H NMR 500 MHz, $CDCl_3$

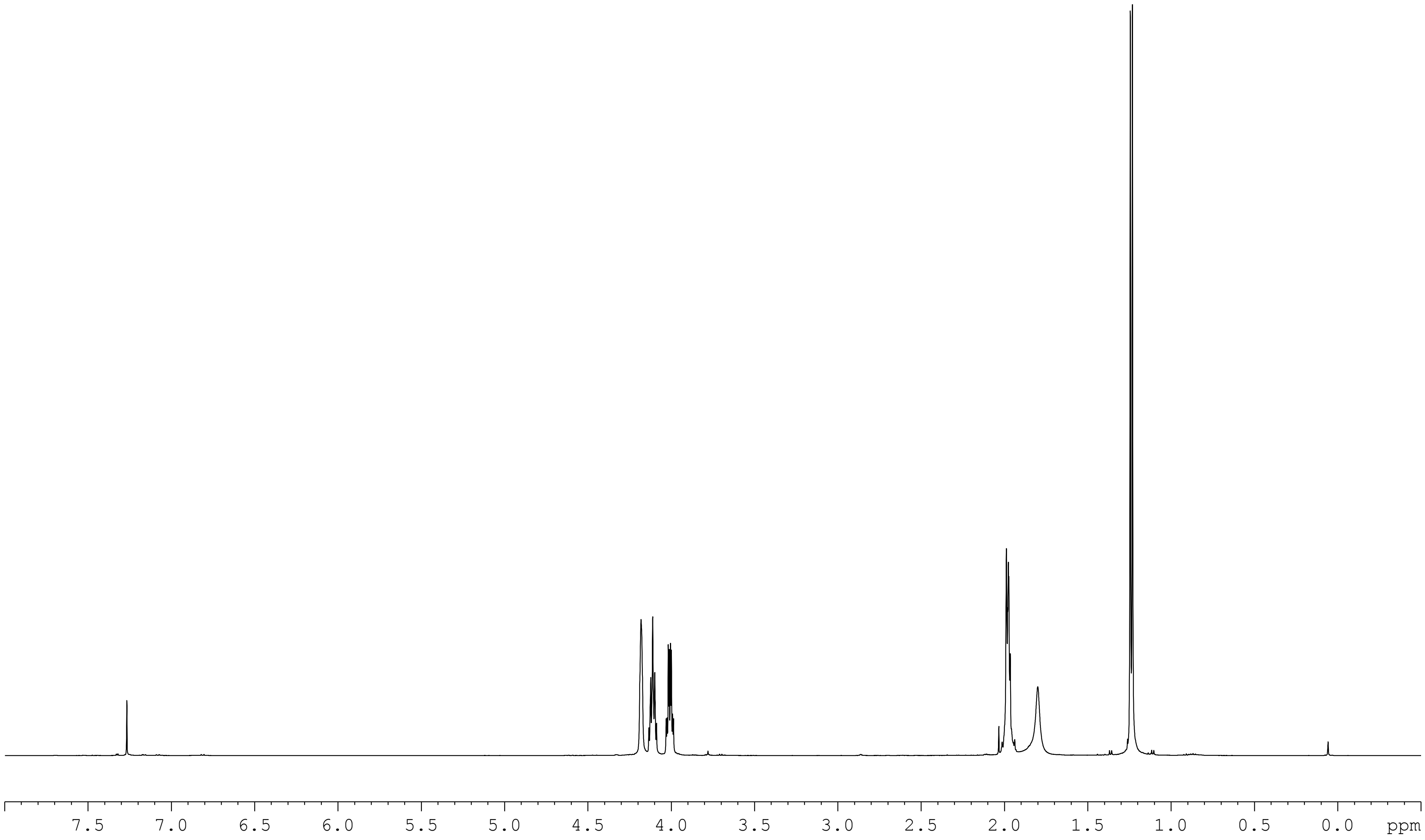

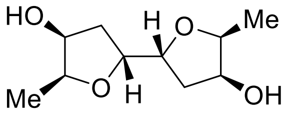

16

^{13}C NMR (DEPT Q), 125 MHz, $CDCl_3$

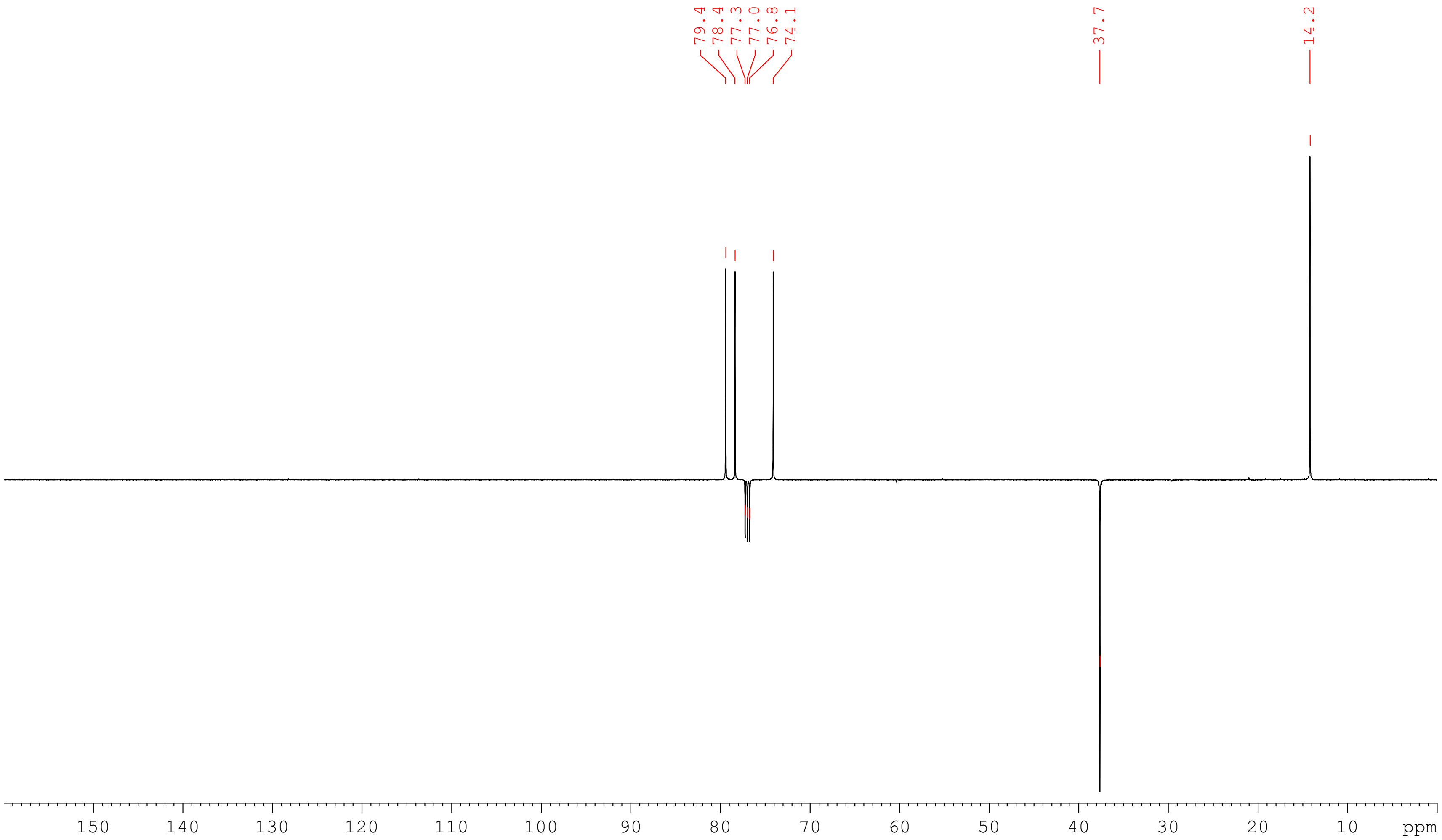

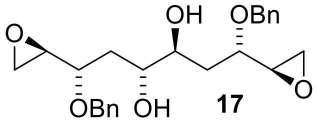

17

^{1}H NMR 400 MHz, $CDCl_3$

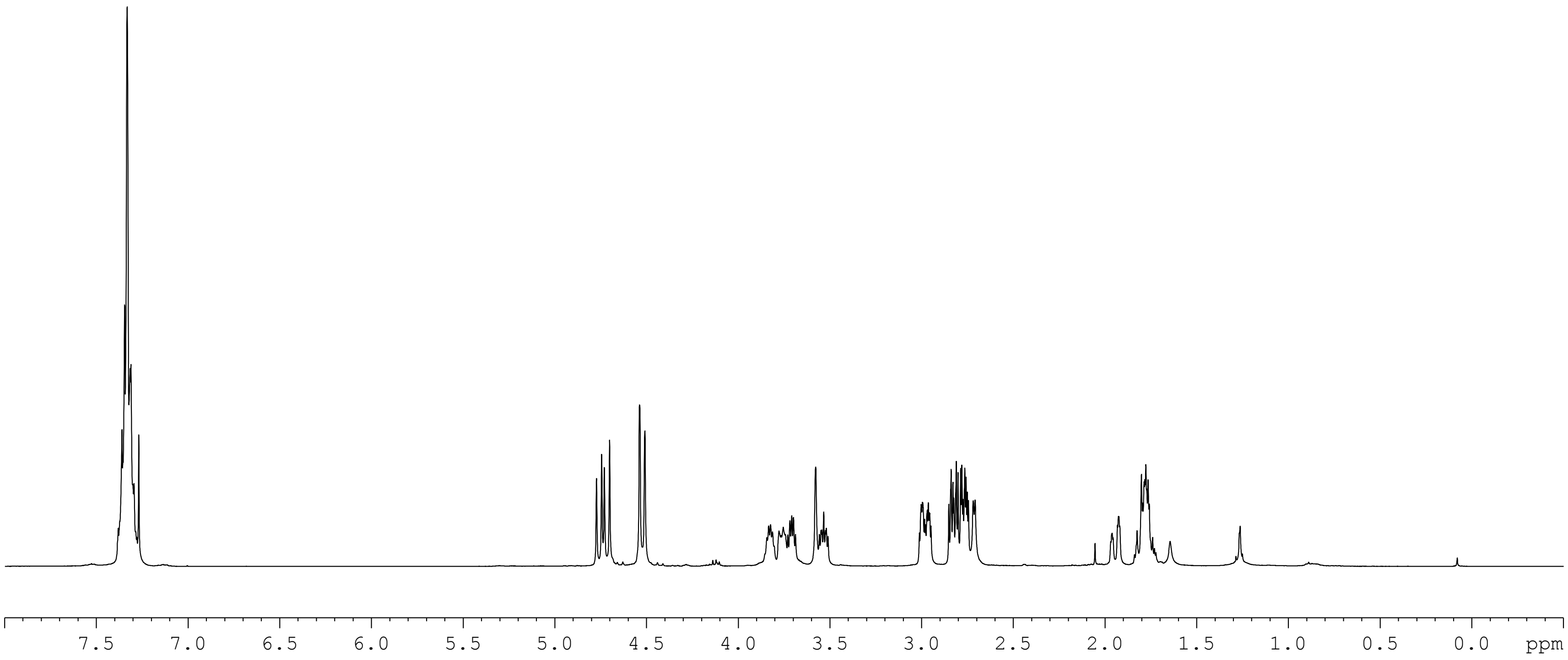

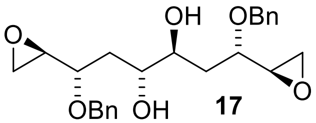

17

^{13}C NMR, 62.5 MHz, $CDCl_3$

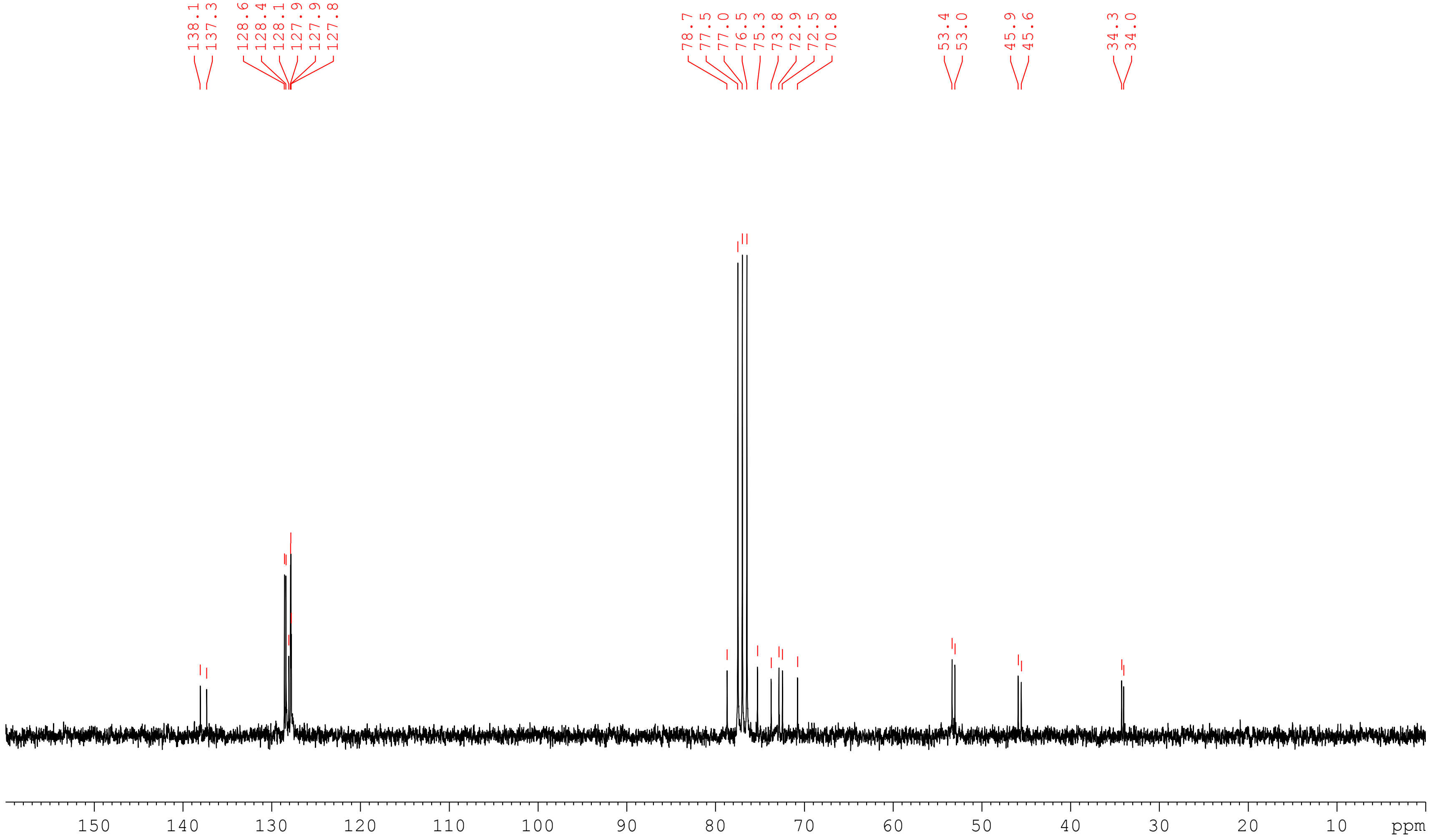

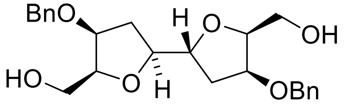

^1H NMR 400 MHz, $CDCl_3$

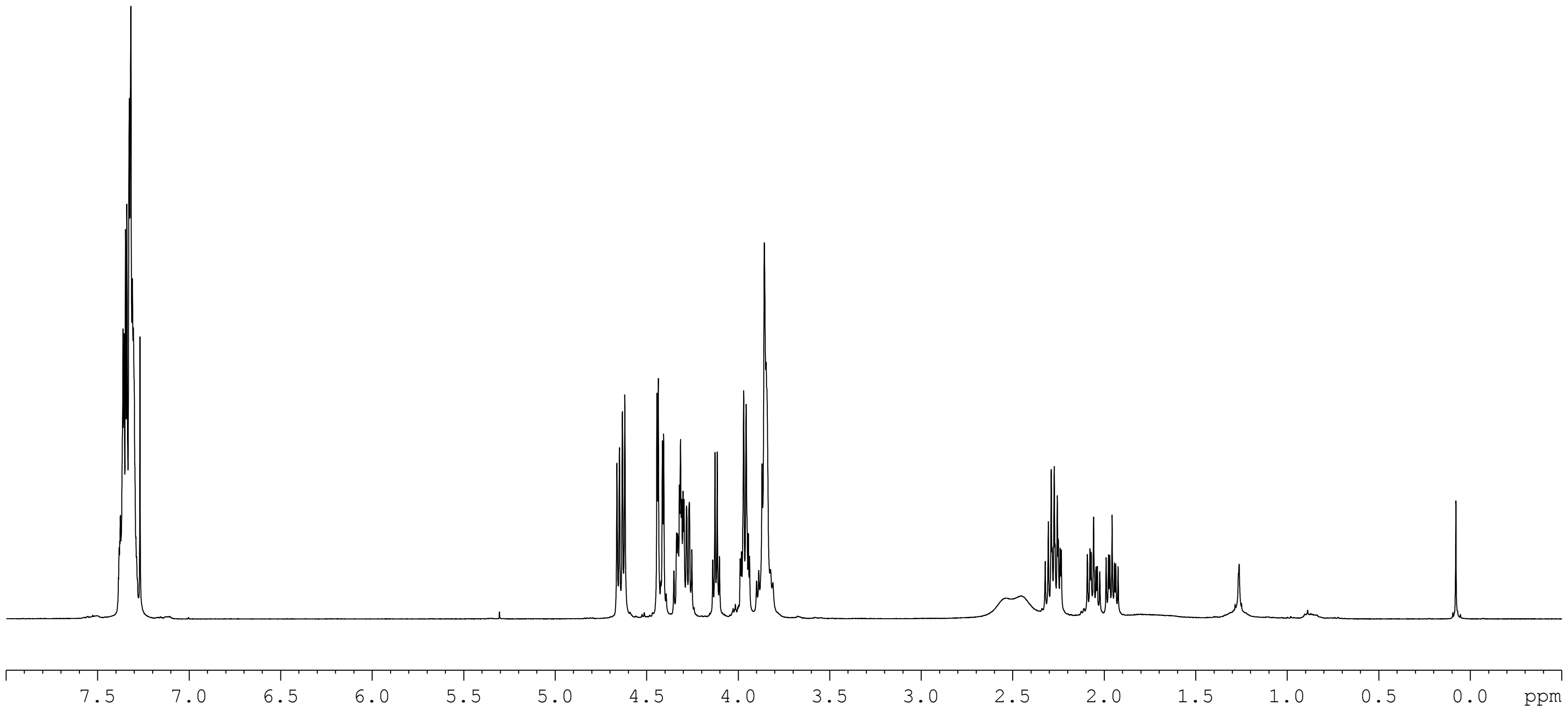

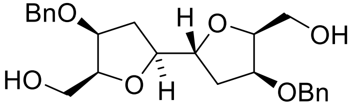

^{13}C NMR, 100 MHz, $CDCl_3$

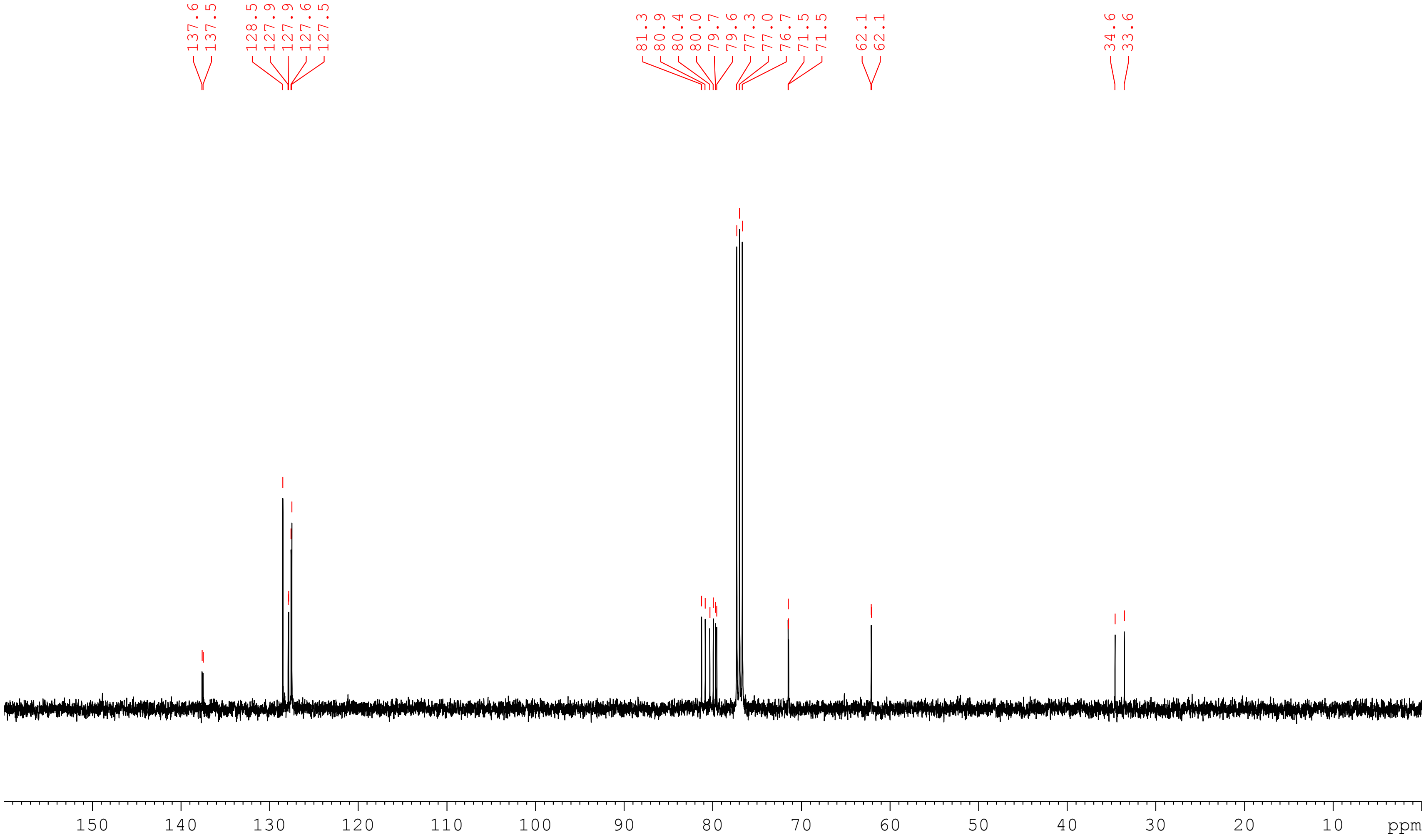

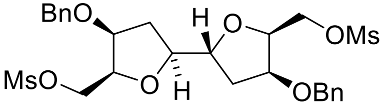

^{1}H NMR 500 MHz, $CDCl_3$

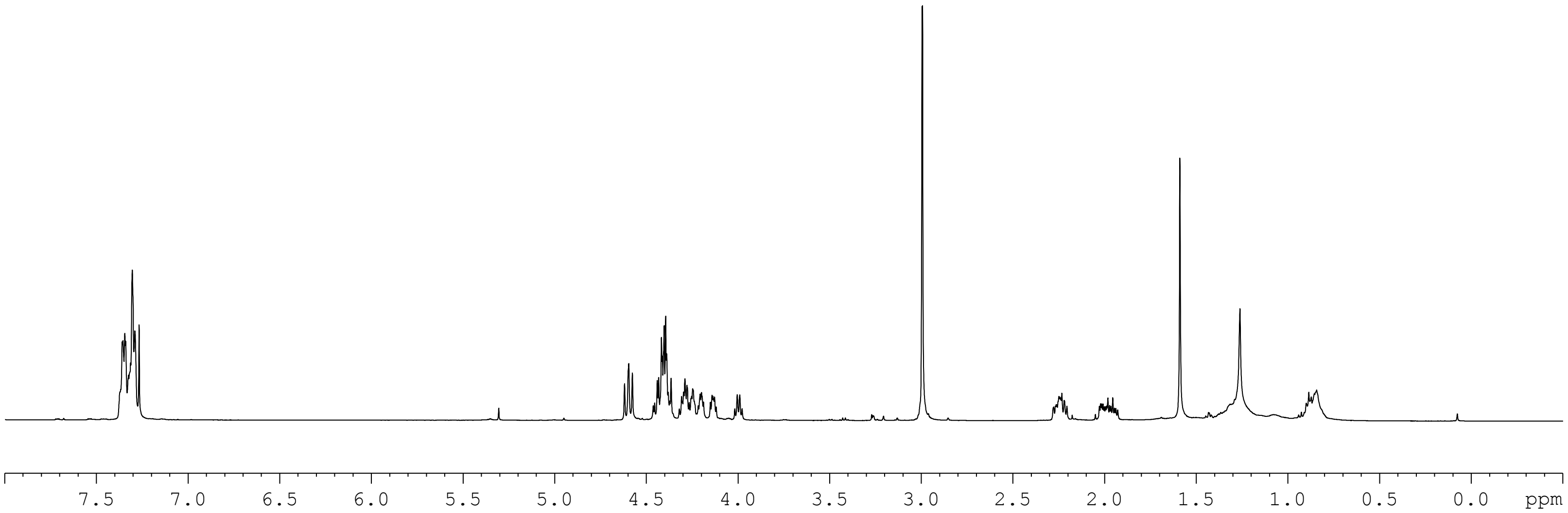

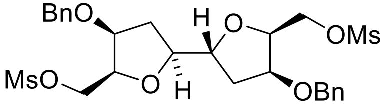

^{13}C NMR (DEPTQ), 125 MHz, $CDCl_3$

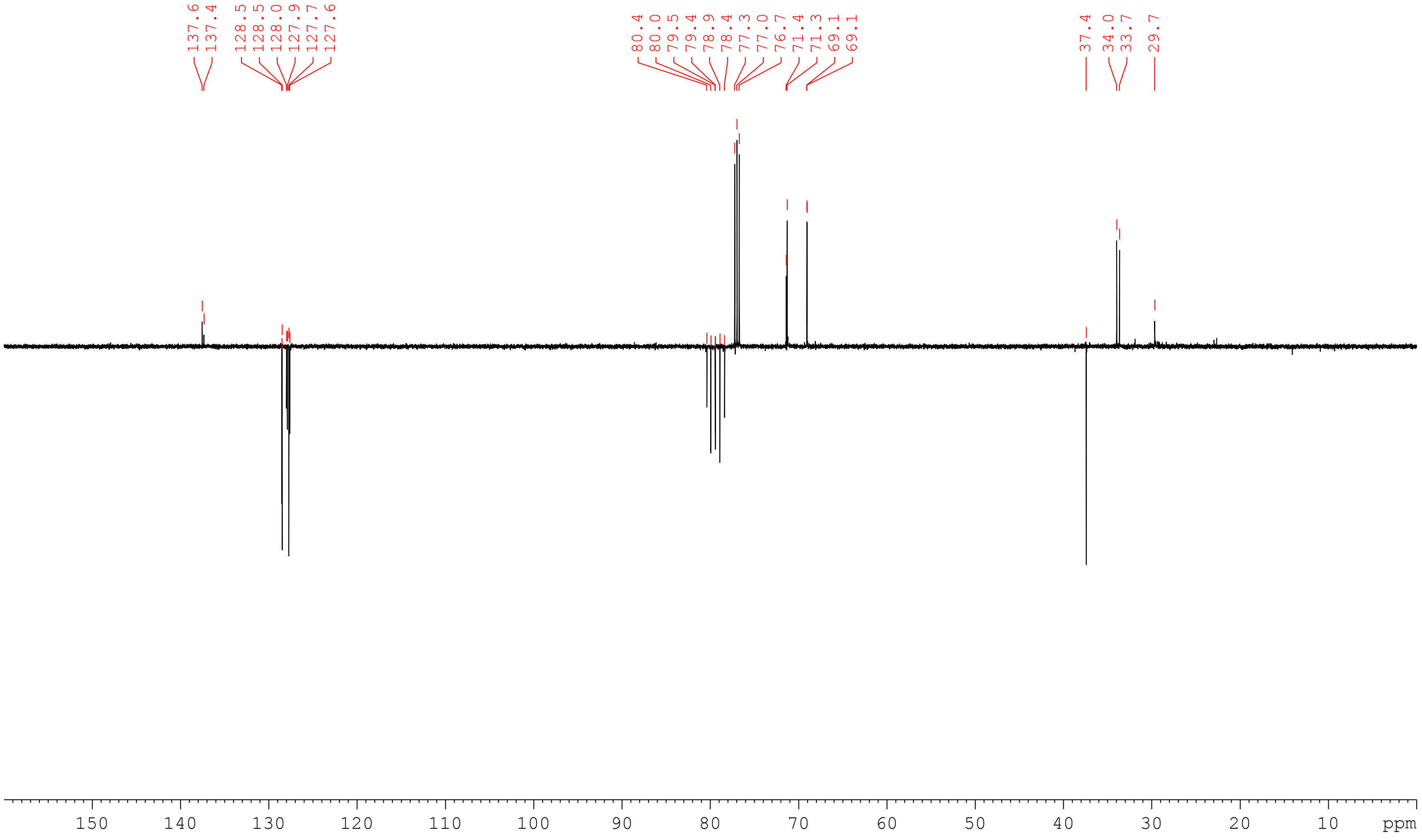

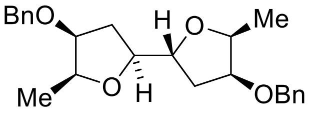

^{1}H NMR 500 MHz, $CDCl_3$

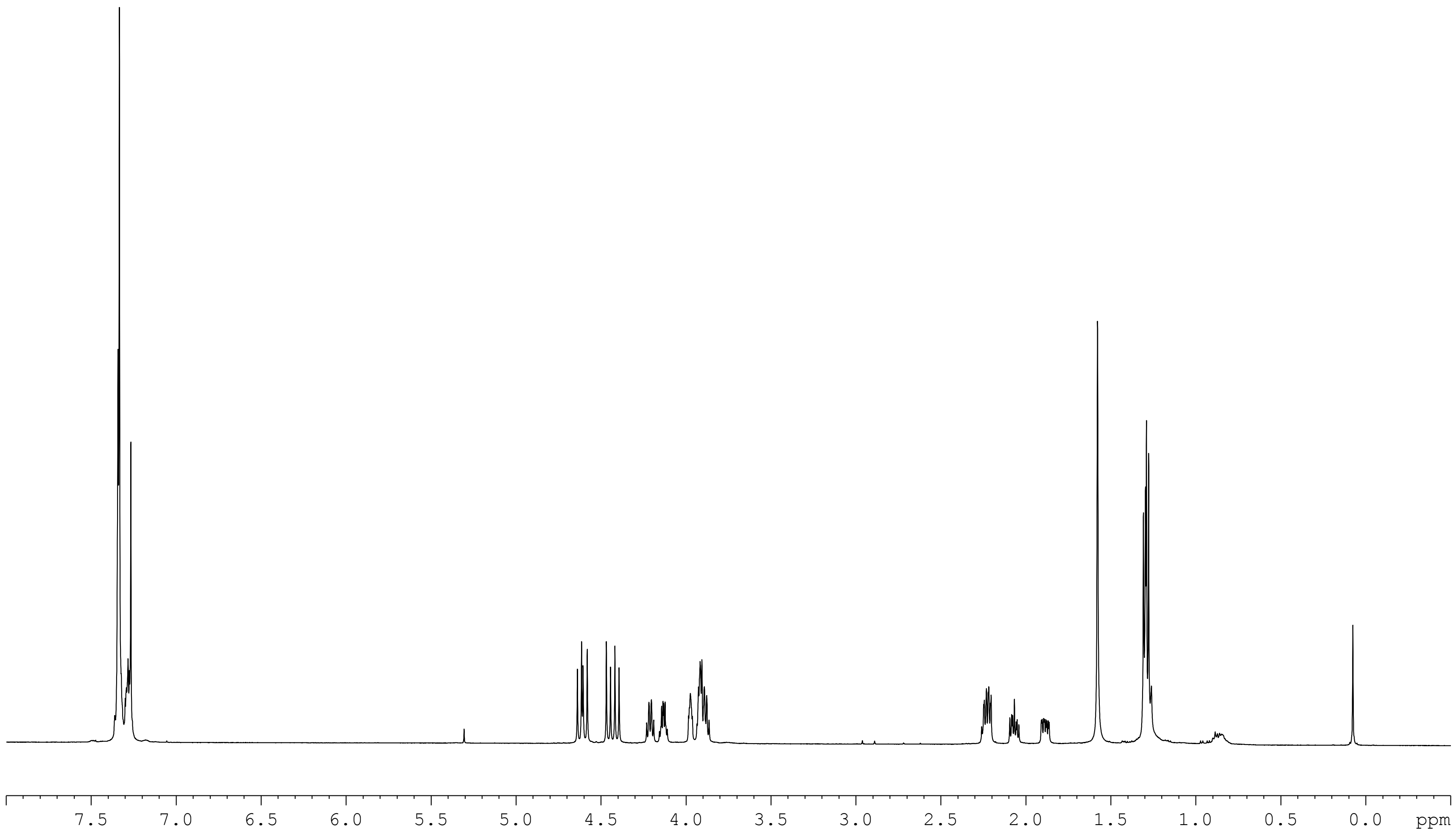

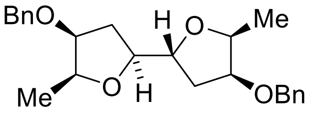

^{13}C NMR, 125 MHz, $CDCl_3$

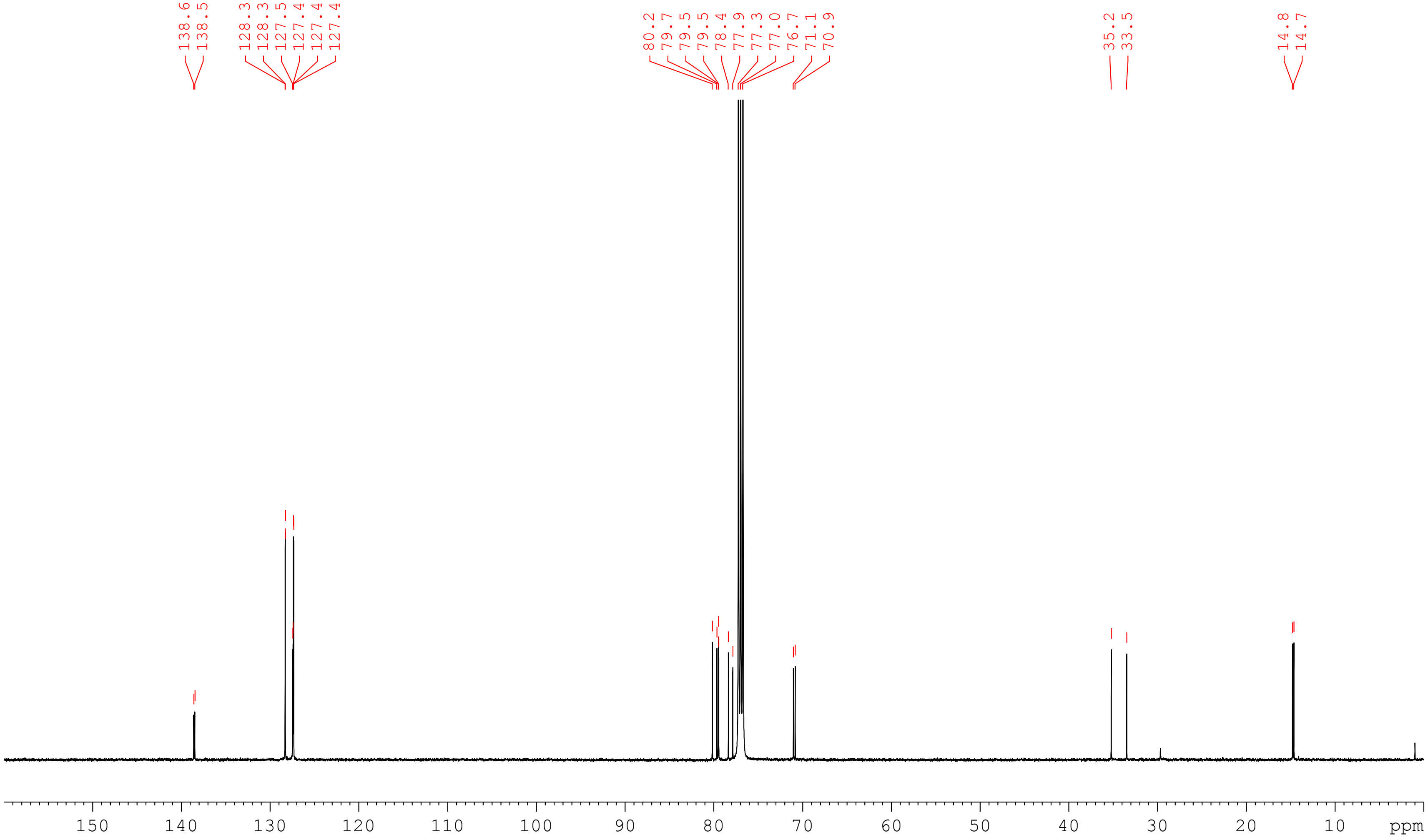

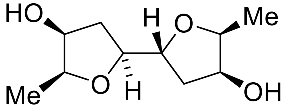

18

^{1}H NMR 500 MHz, $CDCl_3$

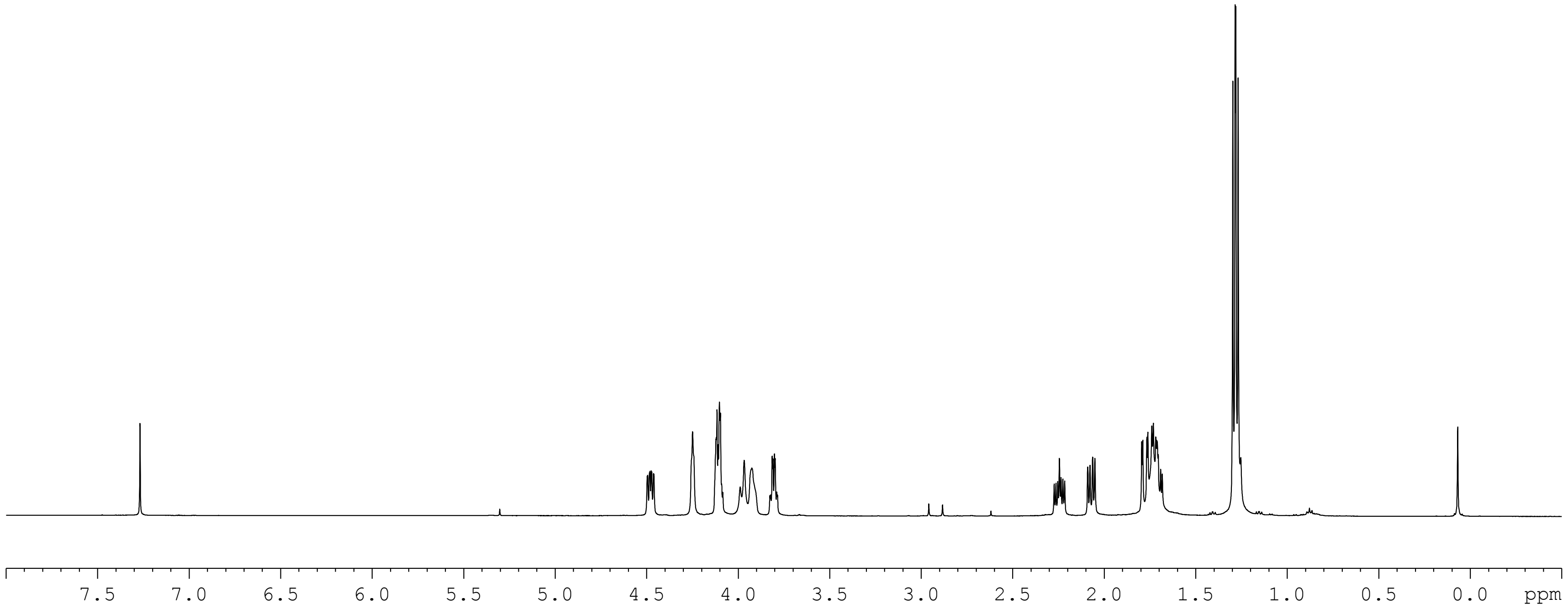

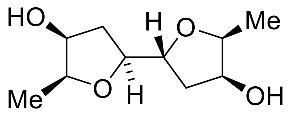

18

^{13}C NMR, 125 MHz, $CDCl_3$

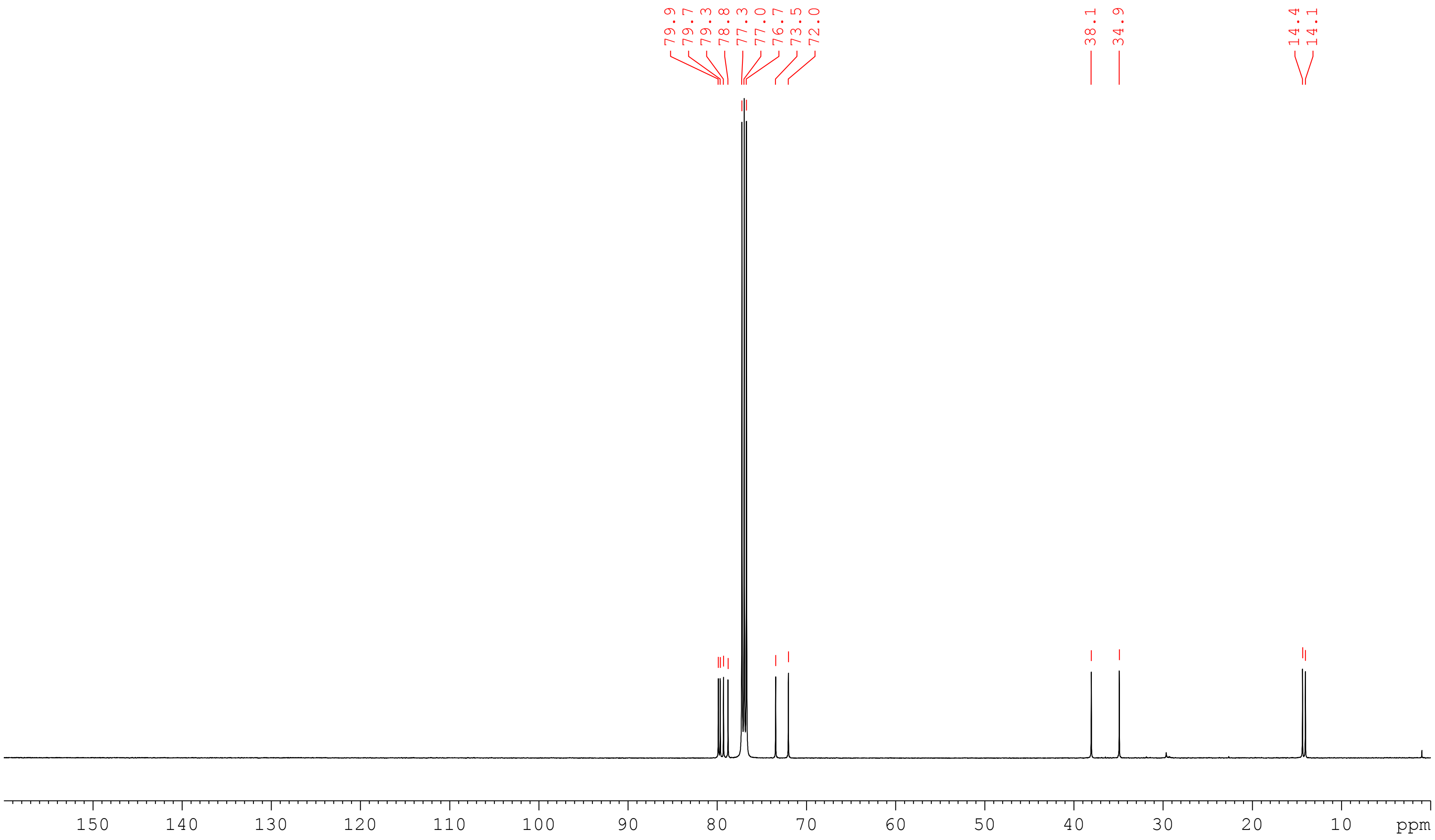

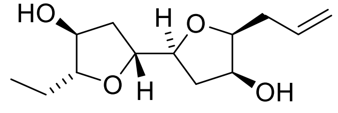

21

^{1}H NMR 400 MHz, $CDCl_3$

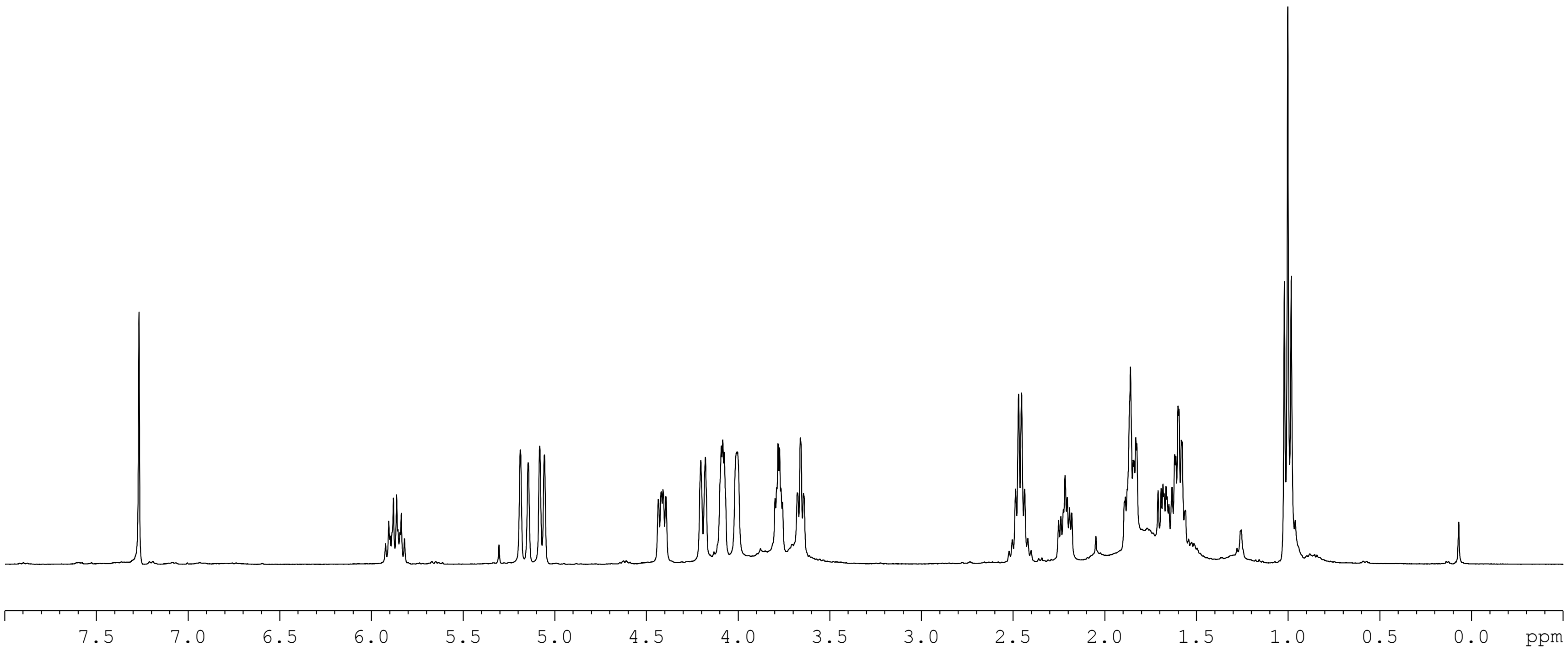

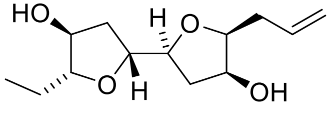

21

^{13}C NMR, 125 MHz, $CDCl_3$

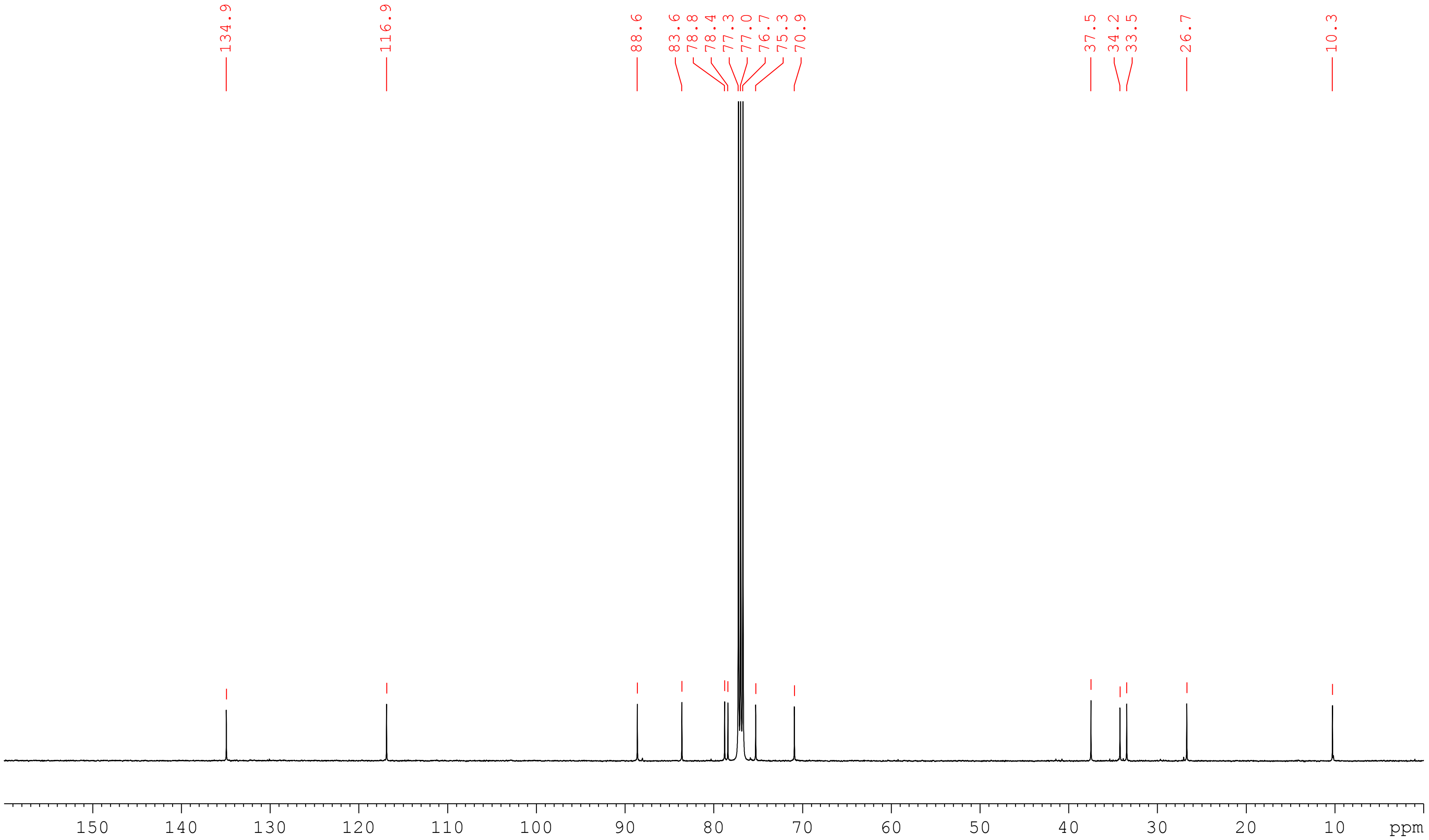

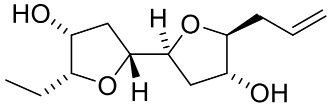

22

^{1}H NMR 500 MHz, $CDCl_3$

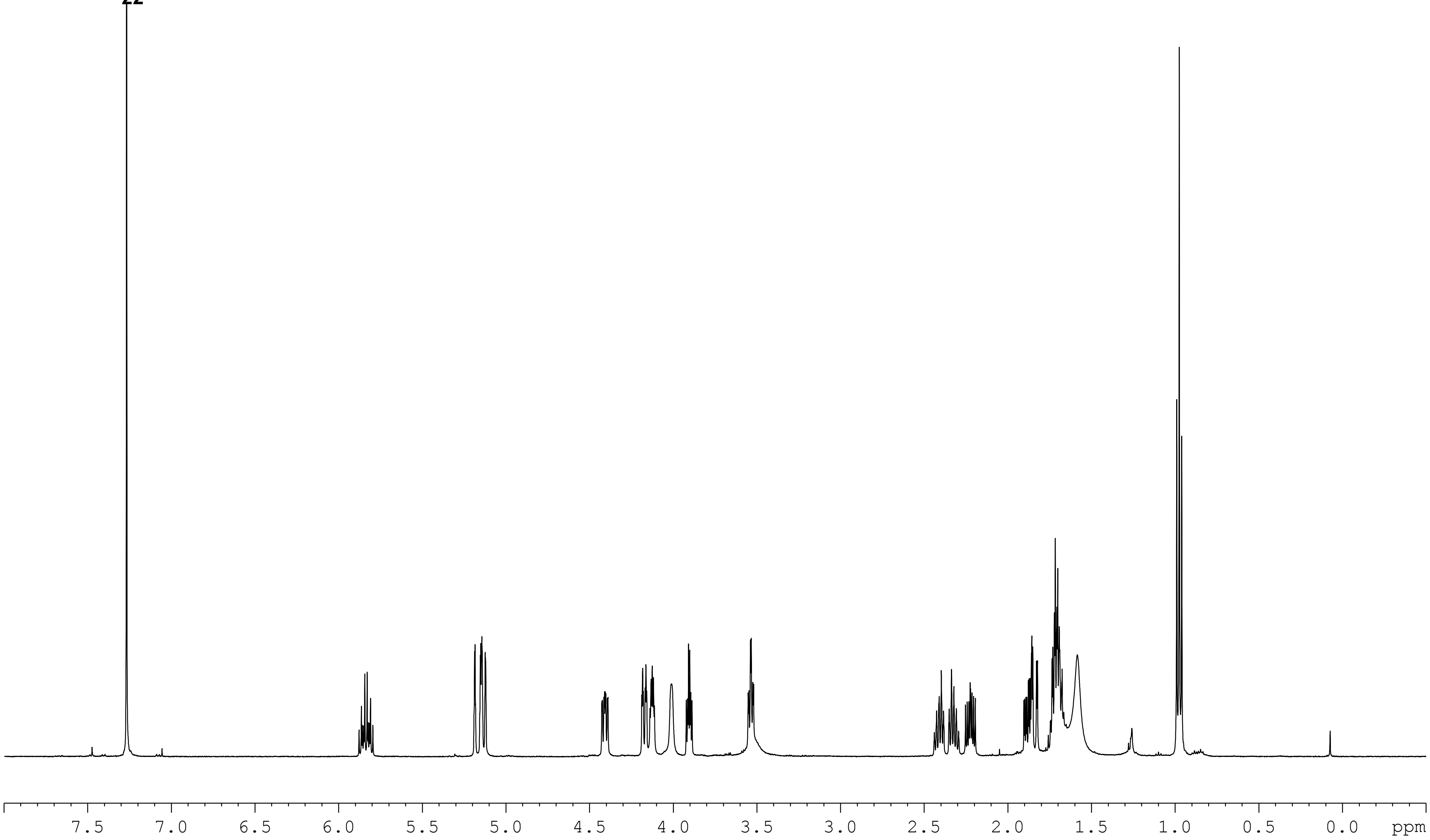

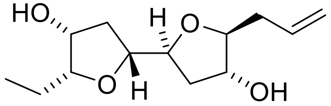

22

^{13}C NMR, 125 MHz, $CDCl_3$

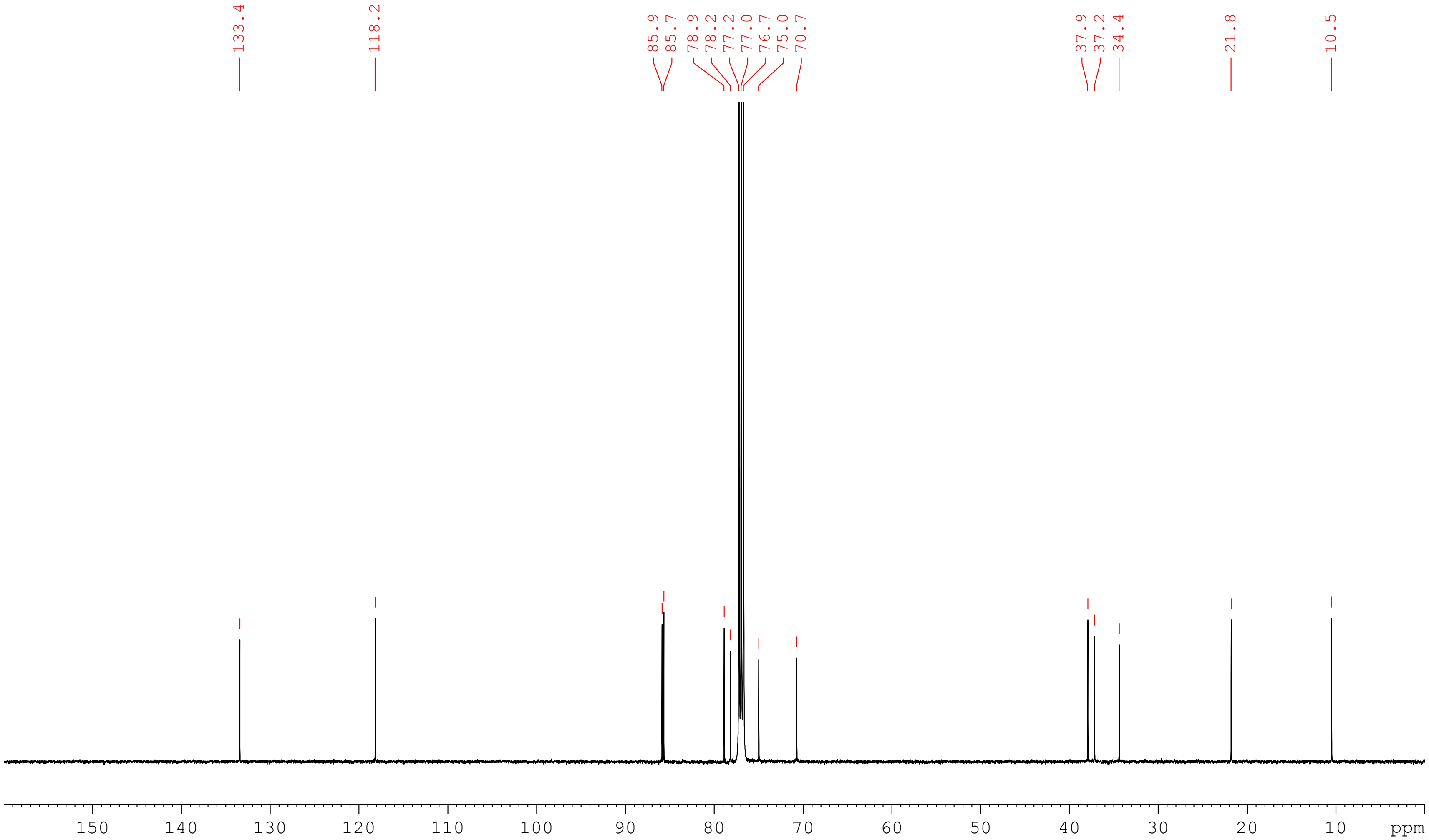

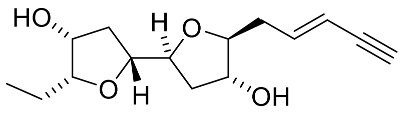

5b

^{1}H NMR, 500 MHz, $CDCl_3$ of synthetic laurefurenyne B (**5b**)
containing a small amount of ethanol

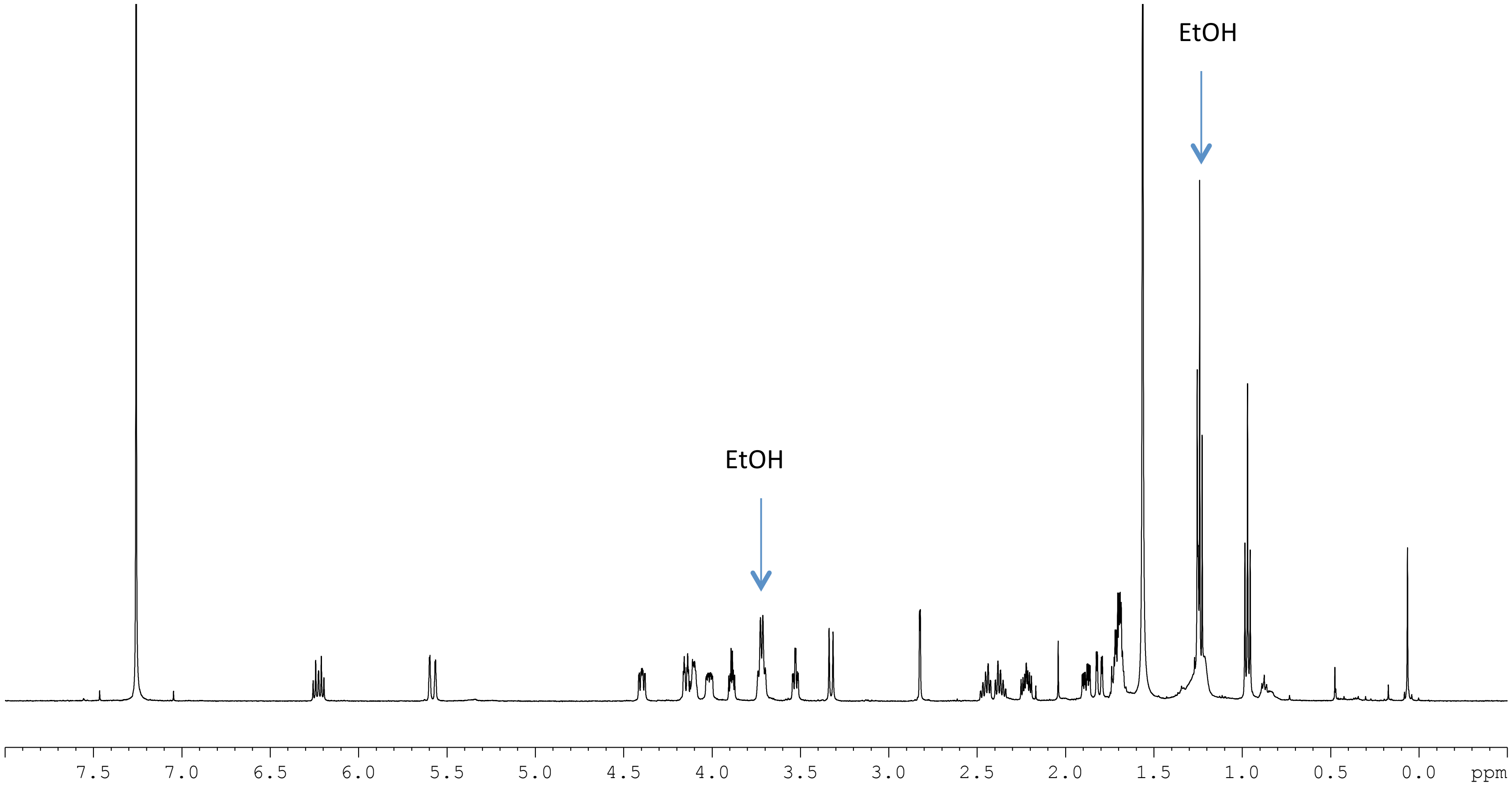

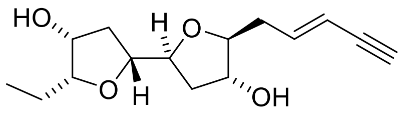

5b

^{1}H NMR, 500 MHz, $CDCl_3$ of synthetic laurefurenyne B (**5b**)

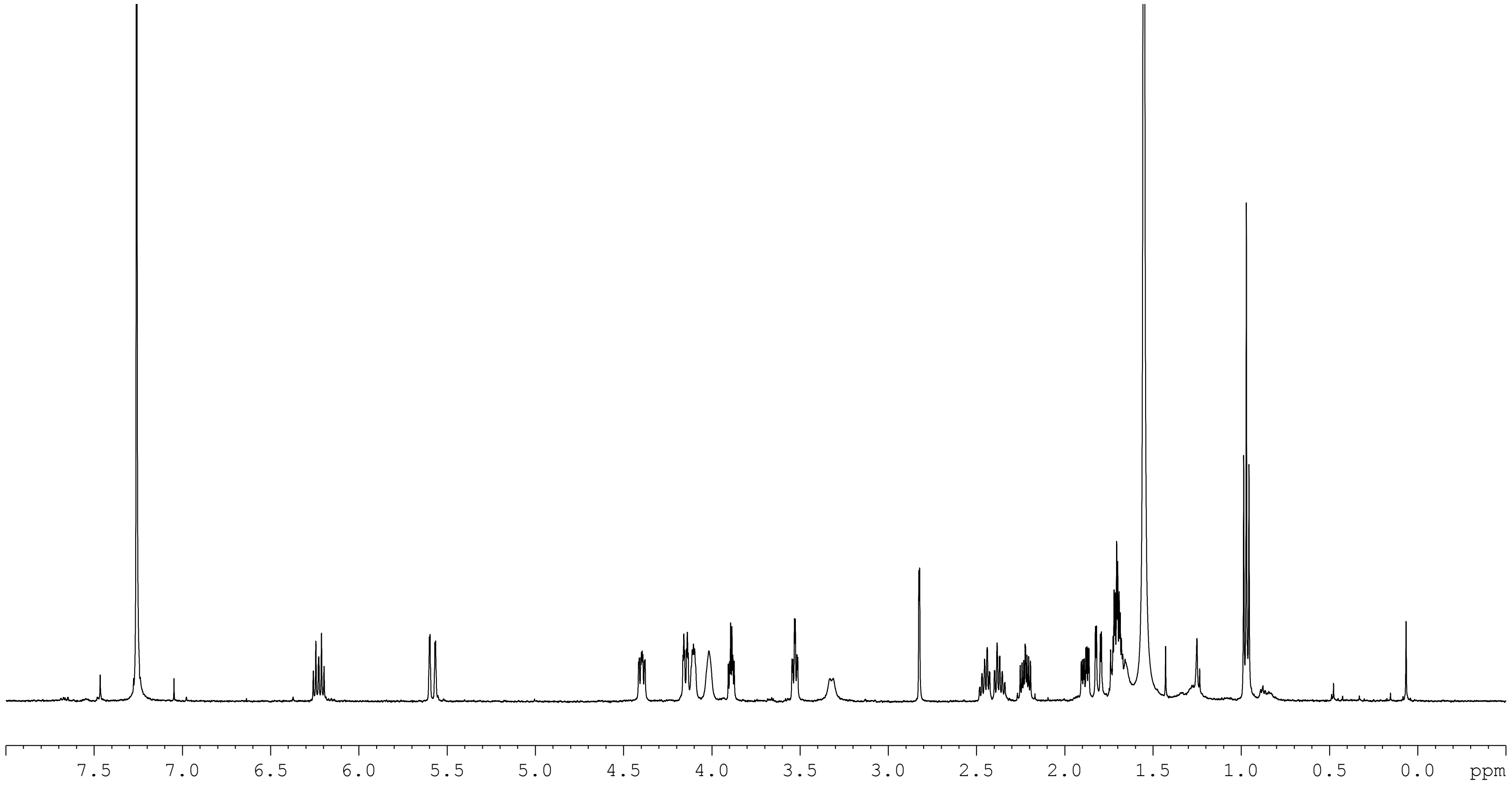

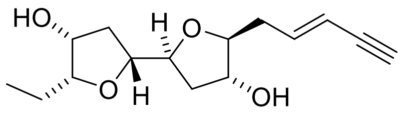

5b

^{13}C NMR, 125 MHz, $CDCl_3$ of synthetic laurefurenyne B (**5b**) - inset is expansion of 76-78 ppm region showing resonance at 76.9 ppm

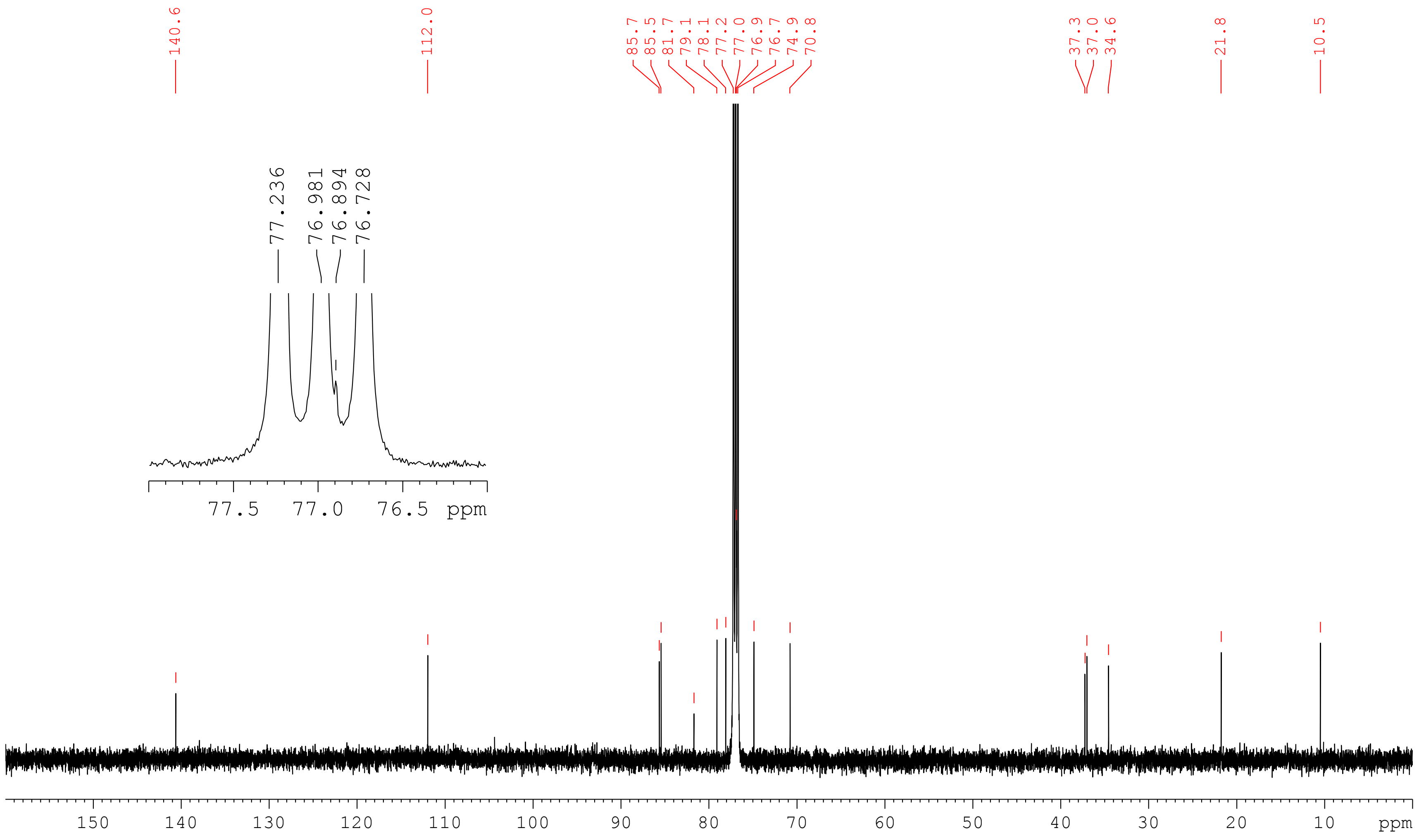

2.1 Benchmarking the GIAO calculations

To assess the performance of our chosen DFT methodology in accurately describing ^{13}C and ^{1}H chemical shifts a comparison was made against two experimental data sets. The first set is extremely large, and contains all molecules from the *NMRshiftDB* (nmrshiftdb.nmr.uni-koeln.de/) containing only C, H, N, O and F with zero/one rotatable bonds and fewer than 25 heavy atoms; the second set comprises fully characterized synthetic intermediates recently prepared in the Burton laboratory[1] with similar patterns of connectivity to Laurefurenynes A and B.[2]

Small molecule data set: For 113 small molecules taken from the *NMRshiftDB* the ^{13}C chemical shifts were computed at the GIAO-mPW1PW91/6-311G(d,p)//wB97XD/6-31G(d) level of theory[3] with a CPCM model[4] of $CHCl_3$ present for both optimization and shielding tensor calculations. Tantillo and co-workers have recently demonstrated that GIAO calculations of chemical shifts performed at the mPW1PW91 level of theory perform well in the structural reassignments of Aquatolide and Nobilisitine A and in the assignment of the N-methylwelwitindolinones.[5,6] The DFT performance is demonstrably good (without any form of linear scaling applied), showing an excellent linear fit (gradient = 1.02, intercept = 2.37 ppm, R^2 = 0.9985) with respect to the experimental shifts obtained in chloroform. Following linear scaling[7] these computational data exhibit a mean unsigned error (MUE) of 1.6 ppm with standard deviation (SD) of 1.5 ppm with respect to the experimental shifts. The distribution of errors following scaling, shown below (RHS) is similar to that obtained by Smith and Goodman[8] at the B3LYP/6-31G(d) level and may be described by a Gaussian or t-distribution, thus making the the DP4 metric applicable to our current work. All of our computed DP4 values were obtained using Goodman's orginal t-distribution parameters: 11.4 (^{13}C) and 14.2 (^{1}H) degrees of freedom with a standard deviation of 2.31 ppm (^{13}C) and 0.18 ppm (^{1}H); using the SD value of 1.5 ppm obtained over our large data set increases the certainty of our structural assignments according to ^{13}C DP4 probabilities, strenghtening the confidence we have in the structural prediction from ^{13}C chemical shifts.

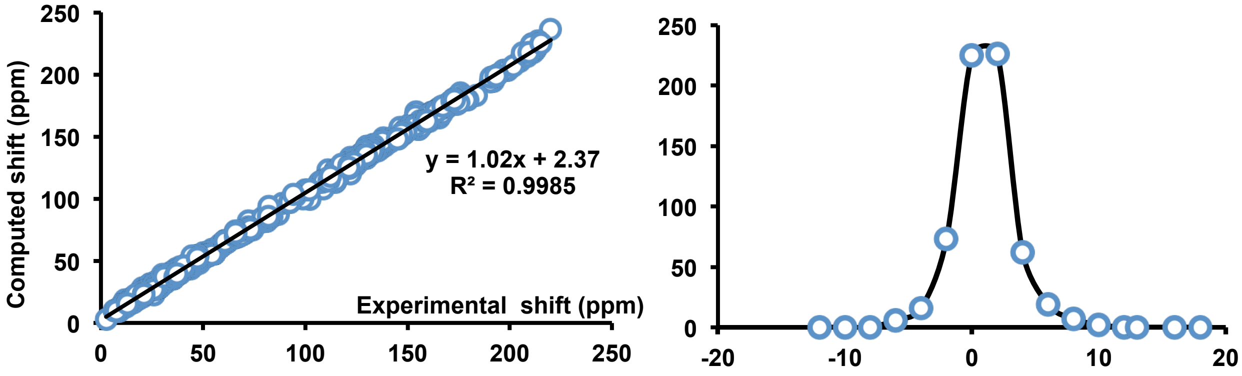

Comparison of Experimental ^{13}C shifts against mPW1PW91/6-311G(d,p)//wB97XD/6-31G(d) GIAO shifts computed in chloroform, without scaling (values in ppm). Values were converted from shielding tensors using a value of 188.6 ppm for TMS, obtained with the same computational protocol.

Small molecule test set: compound IDs containing only C, H, N, O, F:

10005679	10008644	10008825	10008984	10009298	10005696	10008646	10008830	10008988	10017309
10005697	10008655	10008837	10008989	10019745	10005734	10008656	10008838	10008990	10019915
10005737	10008657	10008845	10008997	10020694	10005738	10008661	10008852	10009023	10022547
10005784	10008662	10008854	10009027	10027286	10005922	10008667	10008856	10009033	10887
10005923	10008681	10008858	10009036	20024914	10005925	10008707	10008862	10009045	20036222
10005926	10008743	10008869	10009067	20051734	10005958	10008745	10008870	10009068	20069678
10006102	10008773	10008890	10009069	20069679	10006114	10008778	10008899	10009074	20069680
10006289	10008779	10008900	10009121	20078470	10006291	10008781	10008912	10009152	2470
10006296	10008782	10008921	10009156	2820	10006328	10008791	10008922	10009216	3159
10007821	10008794	10008933	10009280	7901	10008578	10008796	10008955	10009283	7943
10008607	10008798	10008961	10009287	8409	10008622	10008820	10008966	10009289	10008643
10008821	10008970	10009297							

Synthetic intermediate data set: For the four molecules shown,[1] the ^{13}C and ^{1}H chemical shifts were computed at the GIAO-mPW1PW91 level of theory, with both 6-31G(d) and 6-311G(d,p) basis sets with a CPCM model of $CHCl_3$ present for both optimization (at the wB97XD/6-31G(d) level) and shielding tensor calculations. The first three structures are rigid and the (linearly scaled) chemical shifts provide corrected mean absolute errors (CMUEs) below 1.5 ppm for ^{13}C and 0.2 ppm for the ^{1}H shifts, with correlation coefficients above 0.995. For the fourth molecule, which has flexible side chains (conformational averaging of shielding tensors was used) and the enyne group present in the Laurefurenynes, the computational performance is slightly worse, although we would still reasonably expect to obtain a CMUE below 2 ppm / 0.2 ppm for ^{13}C / ^{1}H for the correct structure. With respect to basis set size, surprisingly, there is no systematic improvement on going from double- to a

triple-ζ valence basis set for the shielding tensor calculation. We have used the larger 6-311G(d,p) basis set in the structural assignment of Laurefurenynes A and B to get as close to converged values as is computationally tractable for a large number of structures, although due to favorable error cancellations it may be possible to use a smaller basis set.

	**mPW1PW91/6-31G(d)**	**mPW1PW91/6-311G(d,p)**
	mPW1PW91/6-31G(d)	*mPW1PW91/6-311+G(d,p)*
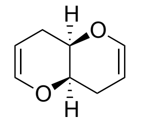	^{13}C: $\delta_{exp} = -1.053\sigma_{calc} + 202.359$ r^2:0.9996<br>*CRMSE* = 0.80 ppm<br>*CMUE* = 0.92 pm<br>^{1}H: $\delta_{exp} = -0.958\sigma_{calc} + 31.107$ r^2:0.9975<br>*CRMSE* = 0.08 ppm<br>*CMUE* = 0.17 pm	^{13}C: $\delta_{exp} = -0.966\sigma_{calc} + 179.886$ r^2:0.9990<br>*CRMSE* = 1.33 ppm<br>*CMUE* = 1.21 pm<br>^{1}H: $\delta_{exp} = -0.943\sigma_{calc} + 30.121$ r^2:0.9944<br>*CRMSE* = 0.12 ppm<br>*CMUE* = 0.19 pm
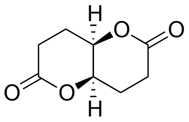	^{13}C: $\delta_{exp} = -1.060\sigma_{calc} + 203.987$ r^2:0.9998<br>*CRMSE* = 0.86 ppm<br>*CMUE* = 0.83 pm<br>^{1}H: $\delta_{exp} = -1.010\sigma_{calc} + 32.664$ r^2:0.9940<br>*CRMSE* = 0.07 ppm<br>*CMUE* = 0.15 pm	^{13}C: $\delta_{exp} = -0.972\sigma_{calc} + 181.278$ r^2:0.9998<br>*CRMSE* = 0.90 ppm<br>*CMUE* = 0.94 pm<br>^{1}H: $\delta_{exp} = -1.008\sigma_{calc} + 32.161$ r^2:0.9981<br>*CRMSE* = 0.04 ppm<br>*CMUE* = 0.14 pm
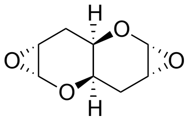	^{13}C: $\delta_{exp} = -1.061\sigma_{calc} + 204.160$ r^2:0.9973<br>*CRMSE* = 0.92 ppm<br>*CMUE* = 0.89 pm<br>^{1}H: $\delta_{exp} = -1.035\sigma_{calc} + 33.370$ r^2:0.9982<br>*CRMSE* = 0.04 ppm<br>*CMUE* = 0.13 pm	^{13}C: $\delta_{exp} = -1.037\sigma_{calc} + 190.290$ r^2:0.9995<br>*CRMSE* = 0.40 ppm<br>*CMUE* = 0.46 pm<br>^{1}H: $\delta_{exp} = -1.057\sigma_{calc} + 33.603$ r^2:0.9986<br>*CRMSE* = 0.04 ppm<br>*CMUE* = 0.13 pm
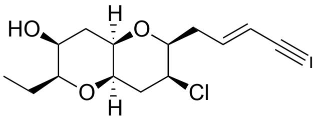	^{13}C: $\delta_{exp} = -1.038\sigma_{calc} + 200.138$ r^2:0.9972<br>*CRMSE* = 1.75 ppm<br>*CMUE* = 1.44 pm<br>^{1}H: $\delta_{exp} = -0.973\sigma_{calc} + 31.387 r^2$:0.9864<br>*CRMSE* = 0.16 ppm<br>*CMUE* = 0.19 pm	^{13}C: $\delta_{exp} = -0.947\sigma_{calc} + 177.785$ r^2:0.9949<br>*CRMSE* = 2.36 ppm<br>*CMUE* = 2.00 pm<br>^{1}H: $\delta_{exp} = -0.934\sigma_{calc} + 29.906$ r^2:0.9833<br>*CRMSE* = 0.18 ppm<br>*CMUE* = 0.20 pm

2.2 Computational Protocol for Predicting the Structure of Laurefurenynes A and B

Maintaining the connectivity of the original structural assignment, we considered all 32 possible diastereomers of Laurefurenyne B. The following numbering system was used:

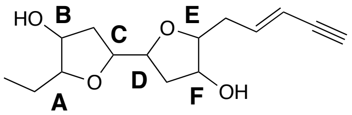

Diastereomer	A:B:C:D:E:F	Diastereomer	A:B:C:D:E:F	Diastereomer	A:B:C:D:E:F	Diastereomer	A:B:C:D:E:F
**1**	*RRRRRR*[a]	**9**	*RRSRRR*	**17**	*RSRRRR*	**25**	*RSSRRR*
**2**	*RRRRRS*	**10**	*RRSRRS*	**18**	*RSRRRS*	**26**	*RSSRRS*
**3**	*RRRRSR*	**11**	*RRSRSR*	**19**	*RSRRSR*	**27**	*RSSRSR*
**4**	*RRRRSS*	**12**	*RRSRSS*	**20**	*RSRRSS*	**28**	*RSSRSS*
**5**	*RRRSRR*	**13**	*RRSSRR*	**21**	*RSRSRR*	**29**	*RSSSRR*
**6**	*RRRSRS*	**14**	*RRSSRS*	**22**	*RSRSRS*	**30**	*RSSSRS*
**S7**	*RRRSSR*	**15**	*RRSSSR*	**23**	*RSRSSR*	**31**	*RSSSSR*
**8**	*RRRSSS*	**16**	*RRSSSS*	**24**	*RSRSSS*	**32**	*RSSSSS*

[a]This is the previously reported stereochemistry of Laurefurenyne A and B.

Outline of the protocol for computing chemical shifts

- For each distereomer a 10,000 step Monte Carlo Multiple Minimum (MCMM) conformational search[9] is performed using the MMFF force field[10] implemented in *Macromodel.*[11] Through *Python* scripts we have also automated a MCMM conformational search interfaced with *AMBER,*[12] *Mopac*[13] *and Gaussian* so that e.g. semi-empirical or UFF levels of theory may also be used optimize and rank conformers. This code may be downloaded for free from *github.com/bobbypaton/compchem* - here we chose to use MMFF since this has previously been shown to describe non-bonding interactions more accurately[14] than e.g. MM2/MM3 or AMBER force fields, and many of conformers possess intramolecular hydrogen bonding interactions.

- Resulting molecular mechanics geometries within 10 kJ/mol of the global energy minimum (a larger window of 20 kJ/mol was used in testing and did not result in any improvement in the results) are automatically parsed (script *mnod2g09.py* is provided below) and reoptimized with *Gaussian09*[15] at the wB97XD/6-31G(d) level with CPCM chloroform. Following these DFT optimizations duplicate conformers are removed (script *CheckDup.py* provided below) to give 2733 conformations in total across all diastereomers as shown immediately below:

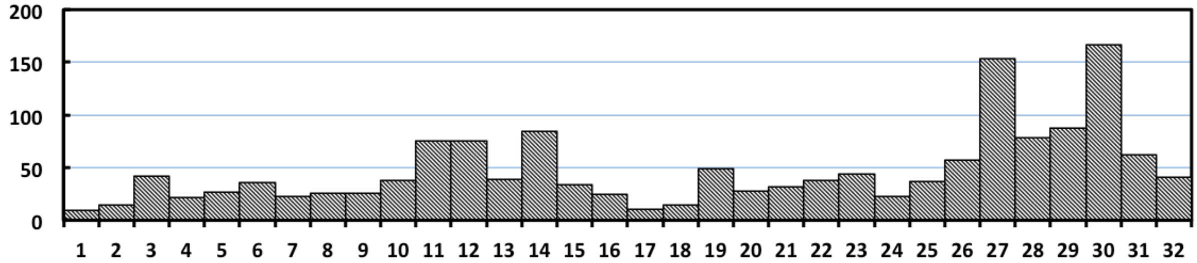

number of optimized conformers for each diastereomer

- For each DFT optimized conformer, shielding tensors are computed at the GIAO-mPW1PW91/6-311G(d,p) level of theory with CPCM chloroform solvation. The conformational ensemble average ^{13}C and ^{1}H shielding tensor is computed from Boltzmann factors at 298K using the electronic energies (script *getnmr.py* below). Using only the energy term implicitly assumes the partition function of each conformer is the same, a necessary approximation since computing the vibrational frequencies is impractical for such a large number of conformers.

- The computed shielding tensors are compared against experimental values to obtain a linear regression, which is then used to convert these tensors into the corresponding chemical shifts. For each diastereomer the root mean squared error (RMSE), mean absolute error (MAE) and correlation coefficient (R^2) are obtained. Goodman's DP4

metric is also computed from the errors assuming an underlying *t*-distribution, using 11.4 (^{13}C) and 14.2 (^{1}H) degrees of freedom with a standard deviation of 2.31 ppm (^{13}C) and 0.18 ppm (^{1}H). The DP4 values penalize single, but prominent, errors much more severely than is the case when taking an arithmetic (or even geometric) mean and thus tends to eliminate many structures from consideration. Qualitatively similar results were also obtained assuming an underlying Gaussian distribution of errors with the same standard deviation. All statistical analysis is fully automated (*compare_NMR_results.py* provided below).

2.3 Results from chemical shift comparison with Laurefurenyne B

Mindful from benchmarking studies that the flexible side chains could contribute to the overall computational error, without contributing diagnostic information about stereochemistry we investigated using the full molecule or a truncated 8-carbon substructure (shown in red) in comparison against experiment.

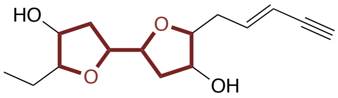

We automated our analysis so we could evaluate the performance for any number of shifts: the objective is not to minimize computational errors across the board, but to increase the separation between best and worst structure. We show results obtained for the whole molecule and for the C_8 substructure below: while both approaches favor the same diasteromer, #6, as the most likely structure, it is notable that the smaller system with fewer atoms gives greater range from best to worst MUE for both ^{13}C and ^{1}H results. For the ^{13}C data, the side chains are a source of essentially systematic error for each diastereomer: the overall errors are reduced after their removal, however, the DP4 probabilities are essentially unchanged which suggests that the side chain carbons contain very little stereochemical information. This is reasonable, particularly for the atoms far removed from any stereogenic centre. The ^{1}H data again favor the same structure #6 in terms of MUE, although the DP4 probability of this most likely structure is increased in the truncated analysis.

Charts show CMAE (ppm) as grey bars, DP4 index as a red line and R^2 as a blue line. NB the axes for DP4 and R^2 are different.

Truncated system, GIAO-mPW1PW91/6-311G(d,p)/wB97XD/6-31G(d) ^{13}C shifts:

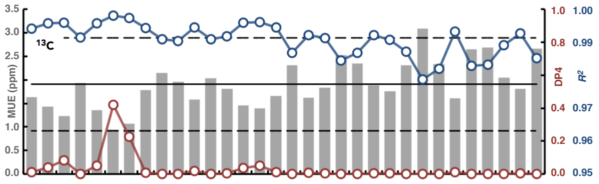

Full system, GIAO-mPW1PW91/6-311G(d,p)/wB97XD/6-31G(d) ^{13}C shifts:

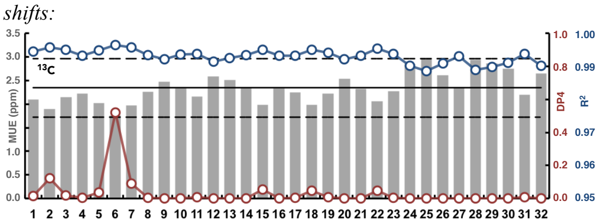

Truncated system, GIAO-mPW1PW91/6-311G(d,p)/wB97XD/6-31G(d) ^{1}H shifts

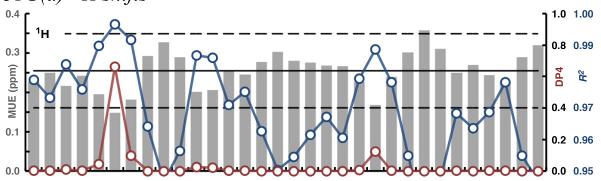

Full system, GIAO-mPW1PW91/6-311G(d,p)/wB97XD/6-31G(d) ^{1}H shifts

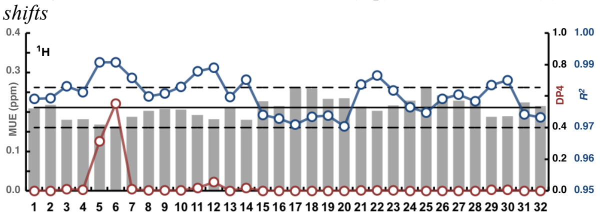

Following DFT optimizations, comparing the Boltzmann weighted chemical shifts against experiment yields for the truncated C_8 system, the smallest mean absolute error of 0.9 ppm and 0.15 ppm for ^{13}C and ^{1}H, which is consistent with the levels of accuracy obtained in our earlier DFT benchmarking studies for rigid pyranopyran systems (**section S1**). For the full system, these values increase to 1.8 ppm and 0.16 ppm for ^{13}C and ^{1}H, which are consistent with the results obtained for the benchmark pyranopyran system with flexble side chains. The correlation coefficient makes it extremely difficult to distinguish between any structures since it is very close to 1 for all structures. However, the DP4 metric strongly favors a single diastereomer, #6, from the ^{13}C data and ^{1}H data. The horizontal lines plotted indicate the mean absolute error across all diastereomers (bold) and ±2 standard deviations (dashed). Only diastereomer 6 was predicted to lie at or below the lower limit for both sets of chemical shifts, and has the highest DP4 metric of 52% / 54% of any diastereomer for both nuclei - we have now successfully determined this is the correct structure for Laurefurenyne B. The previously reported structure is diastereomer 1, however, on the basis of calculation alone we would not have ranked this as a likely candidate structure since it is not amongst the lowest CMAEs and the DP4 metric is less than 0.5%. The truncated model system produces larger variation between the structures both in terms of the average errors and the R^2 values, and so assignment using these metrics can be made more confidently; the DP4 metric performs equally for both systems, demonstrating its utility in distinguishing between correct and incorrect structures.

2.4 Are DFT Optimizations Necessary?

Charts show CMAE (ppm) as grey bars, DP4 index as a red line and R^2 as a blue line.

GIAO-mPW1PW91/6-311G(d,p)/MMFF ^{13}C shifts (top) and ^{1}H shifts (bottom):

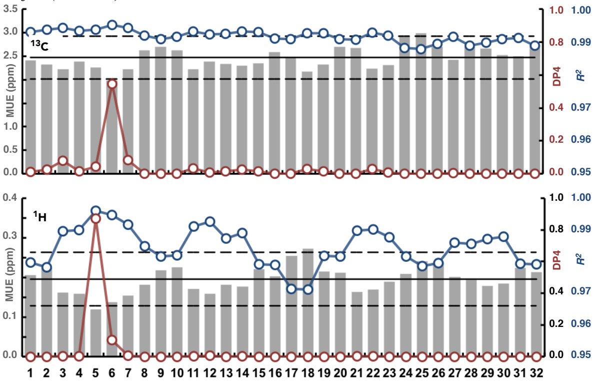

Given the large number of conformations, a computationally less expensive solution is to compute shielding tensors using the MMFF geometries rather than optimizing the structures with DFT. Any systematic errors in geometries (e.g. bond lengths) introduced by such an approach is to some extent removed by linear scaling of the computed chemical shifts, and the DFT single point energy in calculation of the shielding tensor may be used to compute the Boltzmann factor. In our case the computational saving is less than an order of magnitude (around 5-fold acceleration): over the ca. 2800 conformations studied the total CPU time taken for full DFT optimization and NMR calculations is 18,500 CPU hours compared with 3,800 hours taken omitting the DFT optimization step. A comparison of the results using the MMFF geometries (left) against the full DFT results (above) suggests that for the ^{13}C shifts they are qualitatively similar. A closer inspection shows that the best MAE is larger for the MMFF geometries (1.99 ppm vs. 1.75 ppm for wB97XD geometries) such that the range between best and worst structure is compressed for the molecular mechanics results. This may make assignment more difficult, although the DP4 metric still favors the correct diastereomer #6. The ^{1}H results now, however, favor diastereomer #5 over #6 indicating that due to the small differences between correct and incorrect structures, DFT optimization, if it is feasible computationally, is to be preferred.

2.5 Comparison of the previous and reassigned structures of Laurefurenyne B

Charts show errors Δδ (ppm) as grey bars, defined as predicted – experimental chemical shift, for each atom.

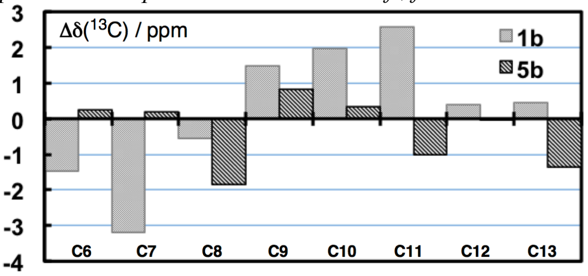

GIAO-mPW1PW91/6-311G(d,p)/MMFF ^{13}C shifts

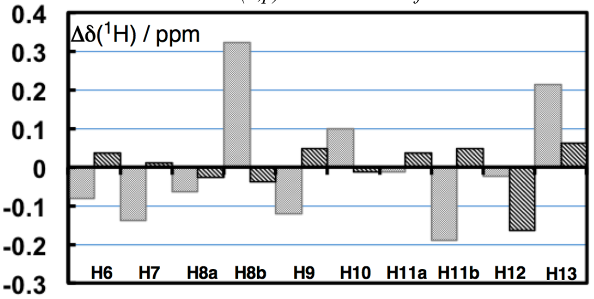

GIAO-mPW1PW91/6-311G(d,p)/MMFF ^{1}H shifts

The errors in computed chemical shifts are shown as a function of position for the previously assigned structure (**1b, main text**) for Laurefurenyne B, and for the structure with the lowest computational error, diastereomer #6, which we subsequently established is correct following total synthesis. From the ^{13}C errors, in particular the previously assigned structure shows largest errors at C6, C7 and C11. C7 is underestimated by more than 3 ppm, which fits with our experimental observation that a *cis*-relationship between the OH and R side chains leads to chemical shifts at C7 around 4-5 ppm lower than for a *trans*-relationship. Thus, the calculated shifts correctly highlight the relative stereochemistry at C6 and C7 is problematic in the original stereochemical assignment. The errors obtained from the correct assignment are reduced at, and in the vicinity of the stereogenic carbons. A similar analysis of the ^{1}H data shows large errors for the previous assignment in the same region of the molecule: at H6 and H7 the shifts are underestimated by 0.3-0.4 ppm further indicating that the relative C6/C7 stereochemistry needed to be revised. Additionally, the shifts at H8a, H8b and H9 have far larger errors than for the correct structure, by over 0.3 ppm, again indicating that the assignment of the right hand furanyl ring is in error. In the same region of the molecule, the correct assignment gives errors smaller than 0.05 ppm.

2.6. Ensemble Averaged Shielding Tensors and Chemical Shifts

Following DFT optimization , Boltzmann factors are computed at 298K from electronic energies in solvent. GAIO-mPW1PW91/6-311G(d,p)/wB97XD/6-31G(d) shielding tensors are shown below (ppm)

Diastereomer:	*DS 01*	*DS 02*	*DS 03*	*DS 04*	*DS 05*	*DS 06*	*DS 07*	*DS 08*	*DS 09*	*DS 10*
**C1**	104.0	103.8	103.7	103.4	103.3	103.6	103.8	104.5	104.6	104.3
**C2**	71.1	71.9	71.4	70.8	71.0	70.7	71.8	72.7	72.8	72.8
**C3**	33.8	33.8	33.9	34.8	34.7	34.3	33.8	31.8	31.9	32.7
**C4**	150.3	146.4	146.0	151.1	150.4	146.0	147.1	149.7	149.6	146.6
**C5**	176.1	176.2	176.2	176.2	176.0	176.0	176.0	175.9	176.3	176.3
**C6**	114.0	113.9	114.1	113.8	114.2	113.9	113.5	112.7	112.2	112.2
**C7**	104.7	104.0	104.2	105.2	105.1	104.7	105.7	104.5	104.5	104.4
**C8**	99.2	98.9	99.8	99.8	100.2	100.3	100.2	100.4	100.3	100.2
**C9**	105.2	104.9	108.0	105.5	105.2	106.2	105.5	105.6	105.7	105.8
**C10**	148.2	149.8	150.2	148.6	147.9	149.4	151.0	151.7	151.5	153.6
**C11**	147.9	147.5	147.4	146.8	151.1	151.3	150.3	149.9	147.8	147.8
**C12**	103.1	103.2	103.2	103.4	103.4	103.3	103.3	102.9	102.7	103.0
**C13**	162.3	162.1	162.1	161.6	162.1	162.2	162.7	163.3	162.8	162.8
**C14**	101.4	96.7	99.7	103.7	103.0	99.0	100.2	101.9	102.2	97.4
**C15**	113.5	111.2	109.4	111.7	112.0	109.6	110.5	112.7	113.7	111.5

Diastereomer:	*DS 11*	*DS 12*	*DS 13*	*DS 14*	*DS 15*	*DS 16*	*DS 17*	*DS 18*	*DS 19*	*DS 20*
**C1**	104.0	104.1	103.8	104.1	104.3	104.4	104.0	103.9	103.9	103.5
**C2**	72.3	72.6	71.4	72.6	72.6	72.5	71.0	71.8	71.6	71.2
**C3**	33.1	32.4	33.3	32.3	32.8	32.3	33.7	33.8	33.5	34.2
**C4**	145.2	149.9	150.6	145.9	145.9	149.5	150.1	146.3	146.5	150.7
**C5**	175.9	175.9	175.7	176.1	176.1	176.2	176.1	176.2	176.1	176.1
**C6**	111.4	111.5	111.1	111.8	111.7	111.8	110.9	110.9	110.5	110.4
**C7**	103.7	104.8	105.8	105.3	104.8	105.1	104.2	103.8	103.6	105.0
**C8**	101.8	101.6	101.5	101.6	100.8	100.8	94.0	94.0	95.0	94.7
**C9**	105.4	105.4	106.2	108.0	105.5	105.6	104.7	104.6	107.1	105.7
**C10**	147.9	146.7	147.9	149.1	149.3	147.2	147.7	149.7	150.1	148.8
**C11**	146.2	146.5	147.9	148.3	148.7	148.6	149.7	149.6	149.0	149.2
**C12**	103.1	103.1	103.1	103.0	103.0	102.9	103.0	103.2	103.1	103.3
**C13**	162.5	162.5	162.8	162.9	163.3	163.3	158.4	158.3	157.7	158.1
**C14**	99.6	103.7	103.8	100.5	97.1	101.8	101.0	96.6	99.0	103.4
**C15**	109.2	111.0	111.3	109.1	110.7	113.3	113.3	111.0	109.3	111.4

Diastereomer:	*DS 21*	*DS 22*	*DS 23*	*DS 24*	*DS 25*	*DS 26*	*DS 27*	*DS 28*	*DS 29*	*DS 30*
**C1**	103.4	103.6	104.0	104.7	104.7	104.3	104.1	104.2	103.9	104.2
**C2**	71.1	70.7	72.4	73.3	73.0	72.9	72.4	72.6	71.7	72.9
**C3**	34.5	34.2	33.2	31.2	31.7	32.7	32.8	32.3	33.0	32.1
**C4**	150.3	146.2	146.7	149.5	149.8	147.0	145.1	150.0	150.6	145.5
**C5**	176.1	176.1	176.3	176.6	176.3	176.4	176.0	176.1	175.2	175.3
**C6**	111.5	111.2	110.1	109.8	109.0	109.1	108.6	108.7	108.5	108.8

**C7**	105.2	104.8	105.0	104.5	105.9	105.8	102.9	104.0	107.7	106.9
**C8**	95.0	95.2	97.3	99.5	96.2	96.2	97.3	97.3	97.8	97.8
**C9**	105.3	106.5	106.4	107.0	105.0	105.1	103.6	104.0	105.9	107.2
**C10**	147.9	149.4	152.1	151.5	151.9	153.9	147.7	145.6	148.5	149.8
**C11**	153.3	153.6	150.6	148.9	149.2	149.1	147.2	146.8	149.0	149.5
**C12**	103.4	103.2	103.1	102.7	102.7	103.0	103.0	103.0	103.0	103.0
**C13**	158.8	159.2	159.1	159.3	157.2	157.3	156.8	156.7	158.0	157.4
**C14**	102.9	98.8	98.7	101.8	102.4	97.6	99.6	104.0	104.0	100.7
**C15**	111.8	109.5	110.8	113.2	113.6	111.3	108.9	110.8	111.3	109.2

**Diastereomer:**	*DS 31*	*DS 32*
**C1**	104.3	104.5
**C2**	72.6	72.7
**C3**	32.6	32.1
**C4**	145.1	149.8
**C5**	175.8	175.9
**C6**	109.0	109.4
**C7**	106.0	107.5
**C8**	96.4	96.9
**C9**	104.2	104.7
**C10**	148.9	147.7
**C11**	149.7	150.1
**C12**	103.0	102.8
**C13**	158.5	157.8
**C14**	97.3	102.0
**C15**	110.2	113.5

**Diastereomer:**	*DS 01*	*DS 02*	*DS 03*	*DS 04*	*DS 05*	*DS 06*	*DS 07*	*DS 08*	*DS 09*	*DS 10*
**H1**	28.89	28.87	28.88	28.86	28.85	28.87	28.87	28.91	28.91	28.90
**H3**	25.91	26.05	26.01	25.93	25.93	26.00	26.00	25.99	25.97	26.06
**H4**	25.07	25.10	25.01	25.12	25.11	24.92	25.02	24.98	24.96	25.06
**H5**	29.38	29.63	29.39	29.31	29.36	29.50	29.56	29.25	29.44	29.67
**H5**	29.26	29.70	29.41	29.36	29.28	29.64	29.50	29.49	29.30	29.76
**H6**	28.12	27.83	27.96	27.85	28.01	27.90	28.04	28.13	28.19	27.80
**H7**	28.00	27.98	27.65	27.68	27.62	27.72	27.88	27.95	28.04	28.01
**H8**	29.92	29.99	29.57	29.46	29.91	30.13	29.68	29.74	30.19	30.24
**H8**	29.46	29.43	30.08	29.91	30.20	30.31	30.01	29.47	29.68	29.66
**H9**	27.74	27.66	27.61	27.65	27.34	27.36	27.58	27.69	27.76	27.70
**H10**	27.76	27.78	27.85	27.89	27.79	27.68	27.68	27.64	27.36	27.36
**H11**	29.45	29.40	29.48	29.42	29.68	29.69	29.66	29.64	30.20	30.18
**H11**	29.93	29.93	29.98	30.01	30.24	30.11	29.98	30.05	29.90	29.89
**H12**	27.98	27.94	28.01	27.95	28.04	27.99	27.94	27.75	27.61	27.61
**H13**	28.19	28.13	28.24	28.17	28.26	28.27	28.23	28.18	28.11	28.10
**H14**	30.16	30.22	30.17	30.23	30.23	30.21	30.23	30.21	30.24	30.24
**H14**	30.16	30.22	30.17	30.23	30.23	30.21	30.23	30.21	30.24	30.24

**H15**	30.85	30.88	30.85	30.87	30.84	30.84	30.84	30.82	30.84	30.86
**H15**	30.85	30.88	30.85	30.87	30.84	30.84	30.84	30.82	30.84	30.86
**H15**	30.85	30.88	30.85	30.87	30.84	30.84	30.84	30.82	30.84	30.86

**Diastereomer:**	*DS 11*	*DS 12*	*DS 13*	*DS 14*	*DS 15*	*DS 16*	*DS 17*	*DS 18*	*DS 19*	*DS 20*
**H1**	28.89	28.89	28.88	28.89	28.89	28.91	28.90	28.88	28.89	28.87
**H3**	26.04	26.00	25.95	26.02	26.06	25.96	25.90	26.06	26.04	25.97
**H4**	25.06	25.00	25.07	24.97	25.07	24.98	25.08	25.10	24.95	25.11
**H5**	29.67	29.34	29.42	29.35	29.72	29.30	29.37	29.62	29.43	29.32
**H5**	29.54	29.51	29.39	29.41	29.67	29.43	29.36	29.69	29.37	29.40
**H6**	27.99	28.06	28.05	28.01	27.88	28.11	28.03	27.83	27.94	27.95
**H7**	27.73	27.75	27.74	27.62	27.98	27.96	27.94	27.95	27.59	27.63
**H8**	29.97	29.93	29.93	30.02	29.46	29.43	29.91	29.97	29.51	29.37
**H8**	29.90	29.81	29.43	29.55	30.04	29.97	29.40	29.42	30.01	29.91
**H9**	27.70	27.62	27.80	27.73	27.78	27.84	27.76	27.66	27.61	27.62
**H10**	27.78	27.68	27.85	27.79	27.66	27.66	27.71	27.71	27.78	27.83
**H11**	29.87	29.93	29.45	29.48	29.39	29.44	29.41	29.40	29.42	29.46
**H11**	29.78	29.80	29.94	29.99	29.92	29.91	30.02	30.00	30.06	30.09
**H12**	27.76	27.76	27.75	27.74	27.64	27.65	27.98	27.95	28.00	28.00
**H13**	28.19	28.16	28.14	28.05	28.06	27.95	27.93	27.95	27.96	28.03
**H14**	30.30	30.31	30.33	30.27	30.28	30.25	30.56	30.56	30.59	30.62
**H14**	30.30	30.31	30.33	30.27	30.28	30.25	30.56	30.56	30.59	30.62
**H15**	30.84	30.85	30.83	30.84	30.87	30.85	30.86	30.88	30.87	30.88
**H15**	30.84	30.85	30.83	30.84	30.87	30.85	30.86	30.88	30.87	30.88
**H15**	30.84	30.85	30.83	30.84	30.87	30.85	30.86	30.88	30.87	30.88

**Diastereomer:**	*DS 21*	*DS 22*	*DS 23*	*DS 24*	*DS 25*	*DS 26*	*DS 27*	*DS 28*	*DS 29*	*DS 30*
**H1**	28.86	28.87	28.88	28.92	28.91	28.89	28.89	28.90	28.88	28.90
**H3**	25.94	25.99	26.03	26.01	25.99	26.06	26.05	26.00	25.96	26.04
**H4**	25.11	24.91	25.03	24.92	24.95	25.04	25.05	25.01	25.04	24.95
**H5**	29.36	29.49	29.63	29.27	29.45	29.66	29.65	29.35	29.44	29.41
**H5**	29.28	29.59	29.59	29.54	29.25	29.76	29.57	29.51	29.36	29.50
**H6**	27.99	27.89	27.92	28.18	28.23	27.82	28.00	28.08	27.94	28.07
**H7**	27.62	27.71	27.95	28.02	28.03	27.99	27.72	27.74	27.76	27.70
**H8**	29.90	30.11	29.67	29.66	30.10	30.14	29.88	29.82	30.00	30.08
**H8**	30.18	30.30	29.92	29.79	29.72	29.69	29.87	29.75	29.46	29.58
**H9**	27.33	27.35	27.60	27.72	27.65	27.59	27.84	27.85	27.77	27.69
**H10**	27.76	27.65	27.62	27.60	27.32	27.34	27.78	27.73	27.74	27.71
**H11**	29.67	29.67	29.63	29.60	30.34	30.32	29.96	29.99	29.57	29.62
**H11**	30.31	30.16	30.18	30.36	30.15	30.13	29.87	29.85	30.04	30.13
**H12**	28.02	27.96	27.88	27.80	27.75	27.73	27.72	27.72	27.63	27.73
**H13**	27.97	27.96	28.11	28.25	27.96	27.96	28.09	28.10	28.17	28.21
**H14**	30.64	30.62	30.47	30.34	30.47	30.44	30.56	30.58	30.36	30.41
**H14**	30.64	30.62	30.47	30.34	30.47	30.44	30.56	30.58	30.36	30.41
**H15**	30.88	30.87	30.84	30.82	30.83	30.84	30.88	30.90	30.85	30.84
**H15**	30.88	30.87	30.84	30.82	30.83	30.84	30.88	30.90	30.85	30.84

**H15**	30.88	30.87	30.84	30.82	30.83	30.84	30.88	30.90	30.85	30.84

**Diastereomer:**	*DS 31*	*DS 32*
**H1**	28.90	28.91
**H3**	26.07	25.98
**H4**	25.06	24.96
**H5**	29.69	29.23
**H5**	29.66	29.44
**H6**	27.80	28.17
**H7**	28.01	28.02
**H8**	29.44	29.48
**H8**	30.04	29.97
**H9**	27.75	27.84
**H10**	27.65	27.62
**H11**	29.54	29.58
**H11**	30.05	30.08
**H12**	27.62	27.62
**H13**	28.04	28.07
**H14**	30.34	30.32
**H14**	30.34	30.32
**H15**	30.85	30.86
**H15**	30.85	30.86
**H15**	30.85	30.86

2.7 Python Scripts for the Automated Analysis of Multiple Conformations

Due to the large number of structures considered in the computational analysis (ca. 2800 separate conformations across the 32 diastereomers) it became necessary to develop a number of automated scripts for job prepration and statistical analysis of the results. These have been made freely available through the web, at *github.com/bobbypaton/compchem,* and are reproduced below in raw text format. In addition, a set of Monte Carlo Multiple Minimum conformational searching scripts, *FullMonte*, that interface with Mopac, AMBER and Gaussian09 have also been developed, so that we are now able to search conformational space at essentially any level of theory, with the obvious caveat that the calculations should be relatively fast. For example where some force field parameters are not available, UFF or even semi-empirical methods may be used instead.

mnod2g09.py*: Takes Macromodel output from a conformational search and generates G09 input files for the lowest energy conformers within a specified range from the global minimum*
Example usage: python mmod2g09.py MMoutput.mae -cuttoff X.X -route "nmr b3lyp/6-31G(d)"
Requires: Macrmodel formatted output file from a conformational search

```
##################################################################
# mmod2g09.py                                          #
# python script to take conformers from Macromodel          #
# conformational search within CUTOFF kJ/mol of global       #
# minimum and convert into Gaussian input format.            #
##################################################################

#Python Libraries
import sys, os
from decimal import Decimal

#Some useful Chemistry arrays
periodictable =
["","H","He","Li","Be","B","C","N","O","F","Ne","Na","Mg","Al","Si","P","S","Cl","Ar","K","Ca","Sc","Ti","V","Cr","Mn","Fe","Co","Ni","Cu","
Zn","Ga","Ge","As","Se","Br","Kr","Rb","Sr","Y","Zr",
            "Nb","Mo","Tc","Ru","Rh","Pd","Ag","Cd","In","Sn","Sb","Te","I","Xe","Cs","Ba","La","Ce","Pr","Nd","Pm","Sm","Eu","Gd","Tb","Dy
","Ho","Er","Tm","Yb","Lu","Hf","Ta","W","Re","Os","Ir","Pt","Au","Hg","Tl",
```

```
        "Pb","Bi","Po","At","Rn","Fr","Ra","Ac","Th","Pa","U","Np","Pu","Am","Cm","Bk","Cf","Es","Fm","Md","No","Lr","Rf","Db","Sg","B
h","Hs","Mt","Ds","Rg","Uub","Uut","Uuq","Uup","Uuh","Uus","Uuo"]

atomicmass = [1.008, 4.003, 6.941, 9.012, 10.81, 12.01, 14.01, 16.00, 19.00, 20.18, 22.99, 24.31, 26.98, 28.09, 30.97, 32.07, 35.45, 39.95, 39.10,
40.08, 44.96, 47.87, 50.94, 52.00, 54.94, 55.84, 58.93, 58.69,
        63.55, 65.39, 69.72, 72.61, 74.92, 78.96, 79.90, 83.80, 85.47, 87.62, 88.91, 91.22, 92.91, 95.94, 99.0, 101.07, 102.91, 106.42, 107.87,
112.41, 114.82, 118.71, 121.76, 127.60, 126.90, 131.29]

def elementID(massno):
                if massno < len(periodictable): return periodictable[massno]
                else: return "XX"

def atomicnumber(element):
        atomicno = 0
        for i in range(0,len(periodictable)):
                if element == periodictable[i]: atomicno = i
        return atomicno

#Write a new Gaussian09 input (*com) file
class writeG09input:

        def __init__(self,file,Ginput,MolSpec):
                print "    Writing", file+"_"+Ginput.Append+".com\n"
                fileout = open(file+"_"+Ginput.Append+".com", "w")
                if hasattr(Ginput, "Mem"): fileout.write("%mem="+Ginput.Mem+"\n")
                if hasattr(Ginput, "Nproc"): fileout.write("%nprocshared="+Ginput.Nproc+"\n")
                if hasattr(Ginput, "Linda"): fileout.write("%nproclinda="+Ginput.Linda+"\n")
                fileout.write("# "+Ginput.Route+"\n\n")
                fileout.write(Ginput.Title+"\n\n")
                fileout.write(str(MolSpec.CHARGE)+" "+str(MolSpec.MULT)+"\n")
                for i in range(0,MolSpec.NATOMS):
                        fileout.write(MolSpec.ATOMTYPES[i])
                        for j in range(0,3):
                                fileout.write("  "+str(Decimal(str((MolSpec.CARTESIANS[i][j])))))
                        fileout.write("\n")
                fileout.write("\n")
                if len(Ginput.Optional) > 0:
                        for option in Ginput.Optional: fileout.write(option+"\n")
                        fileout.write("\n")
                if len(Ginput.Freeze) > 0:
                        for frozen in Ginput.Freeze: fileout.write(frozen+"\n")
                        fileout.write("\n")
                if hasattr(Ginput, "Link1"):
                        fileout.write("--Link1--\n%chk="+Ginput.Link0+"\n")
                        if hasattr(Ginput, "Mem"): fileout.write("%mem="+Ginput.Mem+"\n")
                        if hasattr(Ginput, "Nproc"): fileout.write("%nprocshared="+Ginput.Nproc+"\n")
                        if hasattr(Ginput, "Linda"): fileout.write("%nproclinda="+Ginput.Linda+"\n")
                        fileout.write("# "+Ginput.Link1+"\n\n")
                        fileout.write(Ginput.Title+"\n\n")
                        fileout.write(str(MolSpec.CHARGE)+" "+str(MolSpec.MULT)+"\n")
                        if Ginput.Link1.find("geom=check")==-1:
                                for i in range(0,MolSpec.NATOMS):
                                        fileout.write(MolSpec.ATOMTYPES[i])
                                        for j in range(0,3):
                                                fileout.write("  "+str(Decimal(str((MolSpec.CARTESIANS[i][j])))))
                                        fileout.write("\n")
                                fileout.write("\n")
                        else: fileout.write("\n")
                        if len(Ginput.Optional) > 0:
                                for option in Ginput.Optional: fileout.write(option+"\n")
                                fileout.write("\n")

                if hasattr(Ginput, "Radii"): fileout.write("radii="+Ginput.Radii+"\n\n")

#Define job specificiation for the Gaussian Calculation(s)
class createG09input:
        def __init__(self,file,job):

                def getCutoff(self, job):
                        for keyword in job:
                                if 'CUTOFF' in keyword[0].upper():
                                        self.Cutoff = keyword[1]
                        if not hasattr(self, "Cutoff"):
                                self.Cutoff = "10.0"

                def getAppend(self, job):
                        for keyword in job:
                                if 'APPEND' in keyword[0].upper():
                                        self.Append = keyword[1]
```

```
        if not hasattr(self, "Append"):
            self.Append = "new"


    def getLink0(self, file, job,append):
        for keyword in job:
            if 'LINK0' in keyword[0].upper():
                if keyword[1].find(".chk") == -1:
                    self.Link0 = keyword[1]+".chk"
                else: self.Link0 = keyword[1]
        if not hasattr(self, "Link0"):
            self.Link0 = file+"_"+append+".chk"

    def getLink1(self, file, job):
        for keyword in job:
            if 'LINK1' in keyword[0].upper():
                self.Link1 = keyword[1]


    def getRoute(self,job):
        for keyword in job:
            if 'ROUTE' in keyword[0].upper():
                self.Route = keyword[1]
        if not hasattr(self, "Route"):
            print "\nFATAL ERROR: no Route section specified"
            sys.exit()

    def getTitle(self,file, job):
        for keyword in job:
            if 'TITLE' in keyword[0].upper():
                self.Title = keyword[1]
        if not hasattr(self, "Title"):
            self.Title = file


    def getMem(self, job):
        for keyword in job:
            if 'MEM' in keyword[0].upper():
                self.Mem = keyword[1]


    def getNproc(self, job):
        for keyword in job:
            if 'NPROC' in keyword[0].upper():
                self.Nproc = keyword[1]


    def getLinda(self, job):
        for keyword in job:
            if 'LINDA' in keyword[0].upper():
                self.Linda = keyword[1]


    def getFreeze(self,job):
        self.Freeze = []
        for keyword in job:
            if 'FREEZE' in keyword[0].upper():
                self.Freeze.append(keyword[1])


    def getRadii(self,job):
        for keyword in job:
            if 'RADII' in keyword[0].upper():
                self.Radii = keyword[1]


    def getOptional(self,job):
        self.Optional = []
        for keyword in job:
            if 'OPTIONAL' in keyword[0].upper():
                self.Optional.append(keyword[1])


    getCutoff(self, job)
    getAppend(self, job)
    getLink0(self, file, job, self.Append)
    getRoute(self,job)
    getMem(self,job)
    getNproc(self,job)
```

```
		getLinda(self,job)
		getTitle(self, file, job)
		getFreeze(self,job)
		getRadii(self,job)
		getOptional(self,job)
		getLink1(self, file, job)

if __name__ == "__main__":

	#job specifications
	jobtype = []
	mmodfiles = []

	#input file(s)
	#infiles = []

	# Takes arguments: (1) input file(s) (*maegz) (2) new job parameters
	if len(sys.argv) > 1:
		for i in range(1,len(sys.argv)):
			if sys.argv[i][0:1] == "-" and sys.argv[i][0:3] != "--L":
				if any(sys.argv[i+1]):
					jobtype.append([sys.argv[i],sys.argv[i+1]])
			else:
				if any(sys.argv[i-1]):
					if sys.argv[i-1][0:1] != "-":
						if len(sys.argv[1].split("."))>1:
							#print sys.argv[1]
							if sys.argv[i].split(".")[1]=="maegz":
								mmodfiles.append(sys.argv[i].split(".")[0])


	else:
		print "\nWrong number of arguments used. Correct format: python mmod2g09.py maestrofile -cuttoff X.X -route \"nmr b3lyp/6-
31G(d)\"\n"
		sys.exit()
	#print "1"
	#print mmodfiles
	for file in mmodfiles:
		#print "2"
		Ginput = createG09input(file, jobtype)
		qfile = open(file+"_"+Ginput.Append+".q", "w")
		print ""
		print "o   Applying an energy cutoff of", Ginput.Cutoff, "kJ/mol for the extraction of Macromodel conformers\n"

		#Extract archived Macromodel ouput into readable output

		#Parse - if energy is within CUTOFF of global minimum then save to MolSpec and create G09 input
		print "o   Extracting conformers from", file+".mae\n"
		infile = open(file+".mae","r")
		inlines = infile.readlines()

		line1 =0
		line2 =0
		line3 =0
		line4 =0
		line5 =0

		natom=0
		nconf=0
		confenergy=[]

		# Define molecule
		class MOLECULE: pass
		MolSpec = MOLECULE()

		MolSpec.CHARGE =0
		MolSpec.MULT = 1

		MolSpec.ATOMTYPES=[]
		#Find relative energy
		for i in range(0,len(inlines)):
			if inlines[i].find("f_m_ct") > -1: line1=i
			if inlines[i].find("p_m_ct") > -1: line1=i
			if inlines[i].find("r_mmod_Relative_Potential_Energy") > -1:
				line2=i
			if inlines[i].find(":::") > -1:
				line3=i

			if line1!=0 and line2>line1 and line3>line2:
```

```
nconf = nconf+1
confenergy.append(float(inlines[line2+line3-line1]))

if confenergy[nconf-1] <= float(Ginput.Cutoff):
        print "    Conformer ",nconf," has relative energy", confenergy[nconf-1], "- Creating 
Gaussian Input"

        for j in range(line3,len(inlines)):
                if inlines[j].find("m_atom[") > -1:
                        line4=j
                        natom = int(inlines[line4].translate(None,"m_atom[] {"))
                if inlines[j].find(":::") > -1: line5=j

                #print natom
                if line4!=0 and line5>line4:
                        for k in range(line4,line5+20):
                                if inlines[k].find("i_m_atomic_number") > -1:
                                        index = k-line4+4
                                        print index

                        MolSpec.CARTESIANS=[]
                        #print line4, line5, natom
                        for k in range(line5+1,line5+natom+1):
                                if len(inlines[k].split()) > 6:

MolSpec.CARTESIANS.append([float(inlines[k].split()[2]),float(inlines[k].split()[3]),float(inlines[k].split()[4])])
                                else:

MolSpec.CARTESIANS.append([float(inlines[k].split()[1]),float(inlines[k].split()[2]),float(inlines[k].split()[3])])
                                print inlines[k].split()
                                print inlines[k].split()[20]
                                #print periodictable[int(inlines[k].split()[20])]
                                if len(inlines[k].split()) > 20:

MolSpec.ATOMTYPES.append(periodictable[int(inlines[k].split()[21])])
                                line4 =0
                                line5 =0
                        break

        MolSpec.NATOMS =natom

        Gwrite = writeG09input(file+str(nconf), Ginput, MolSpec)
        qfile.write("g09 "+file+str(nconf)+"_"+Ginput.Append+"\n")
else:
        print "    Conformer ",nconf," has relative energy", confenergy[nconf-1], "- Ignoring due to 
energy cuttof"

line1 =0
line2 =0
line3 =0
```

***getnmr.py**: Computes the Boltzmann averaged shielding tensors for a given element at a specified temperature for any number of G09 output files*
Example usage: python getnmr.py C 298 molecule_conf.out*
Requires: Any number of G09 formatted output files from an NMR shielding tensor calculation

```
##################################################################
# getnmr.py                                              #
# python script to perform a Boltzmann weighting over         #
# conformers computed chemical shifts                    #
##################################################################

#Python Libraries
import subprocess, sys, os, math

#Some useful arrays
periodictable = 
["","H","He","Li","Be","B","C","N","O","F","Ne","Na","Mg","Al","Si","P","S","Cl","Ar","K","Ca","Sc","Ti","V","Cr","Mn","Fe","Co","Ni","Cu","
Zn","Ga","Ge","As","Se","Br","Kr","Rb","Sr","Y","Zr",

"Nb","Mo","Tc","Ru","Rh","Pd","Ag","Cd","In","Sn","Sb","Te","I","Xe","Cs","Ba","La","Ce","Pr","Nd","Pm","Sm","Eu","Gd","Tb","Dy","Ho","Er
","Tm","Yb","Lu","Hf","Ta","W","Re","Os","Ir","Pt","Au","Hg","Tl",
```

```
"Pb","Bi","Po","At","Rn","Fr","Ra","Ac","Th","Pa","U","Np","Pu","Am","Cm","Bk","Cf","Es","Fm","Md","No","Lr","Rf","Db","Sg","Bh","Hs","Mt
","Ds","Rg","Uub","Uut","Uuq","Uup","Uuh","Uus","Uuo"]

def elementID(massno):
	if massno < len(periodictable): return periodictable[massno]
	else: return "XX"

#Parse a G09 output file (*out or *log)
class getoutData:

	def __init__(self, file, elem):

		if not os.path.exists(file+".out"):
			if not os.path.exists(file+".log"):
				print ("\nFATAL ERROR: Output file [ %s ] does not exist"%file)


		def getFORMAT(self, outlines):
			for i in range(0,len(outlines)):
				if outlines[i].find("Gaussian") > -1: self.FORMAT = "Gaussian"; break
				if outlines[i].find("ORCA") > -1: self.FORMAT = "Orca"; break

		def getJOBTYPE(self, outlines, format):
			if format == "Gaussian":
				for i in range(0,len(outlines)):
					if outlines[i].find(" # ") > -1:
						self.JOBTYPE = outlines[i].lstrip(" #").rstrip("\n")
						break


		def getCHARGE(self, outlines, format):
			if format == "Gaussian":
				for i in range(0,len(outlines)):
					if outlines[i].find("Charge = ") > -1:
						self.CHARGE = int(outlines[i].split()[2])
						self.MULT = int(outlines[i].split()[5].rstrip("\n"))
						break

		def getATOMTYPES(self, outlines, format):
			self.ATOMTYPES = []
			self.CARTESIANS = []
			if format == "Gaussian":
				for i in range(0,len(outlines)):
					if outlines[i].find("Standard orientation") > -1:
						standor = i
					if outlines[i].find("Rotational constants") > -1 and outlines[i-1].find("-------") > -1:
						self.NATOMS = i-standor-6
				try: standor
				except NameError: pass
				else:
					for i in range (standor+5,standor+5+self.NATOMS):
						self.ATOMTYPES.append(elementID(int(outlines[i].split()[1])))

	self.CARTESIANS.append([float(outlines[i].split()[3]),float(outlines[i].split()[4]),float(outlines[i].split()[5])])


		def getFREQS(self, outlines, format):
			self.FREQS = []
			if format == "Gaussian":
				for i in range(0,len(outlines)):
					if outlines[i].find("Frequencies") > -1:
						self.FREQS.append(float(outlines[i].split()[2]))
						if len(outlines[i].split()) > 3: self.FREQS.append(float(outlines[i].split()[3]))
						if len(outlines[i].split()) > 4: self.FREQS.append(float(outlines[i].split()[4]))
				if len(self.FREQS) > 0:
					for i in range(0,len(outlines)):
						if outlines[i].find("Zero-point correction") > -1: self.ZPE =
float(outlines[i].split()[2])
						if outlines[i].find("thermal Enthalpies") > -1: self.ENTHALPY =
float(outlines[i].split()[6])
						if outlines[i].find("thermal Free Energies") > -1: self.GIBBS =
float(outlines[i].split()[7])


		def getMULLIKEN(self, outlines, natoms, format):
			if format == "Gaussian":
				for i in range(0,len(outlines)):
					if outlines[i].find("Mulliken atomic charges:") > -1:
```

```
					self.MULLIKEN = []
					for j in range(i+2,i+natoms+2):
						self.MULLIKEN.append(float(outlines[j].split()[2]))


	def getCPU(self, outlines, format):
		days = 0
		hours = 0
		mins = 0
		secs = 0
		if format == "Gaussian":
			for i in range(0,len(outlines)):
				if outlines[i].find("Job cpu time") > -1:
					days = days + int(outlines[i].split()[3])
					hours = hours + int(outlines[i].split()[5])
					mins = mins + int(outlines[i].split()[7])
					secs = secs + int(float(outlines[i].split()[9]))
		self.CPU=[days,hours,mins,secs]


	def getENERGY(self, outlines, format):
		if format == "Orca":
			for i in range(0,len(outlines)):
				if outlines[i].find("FINAL SINGLE POINT ENERGY") > -1 : # Get energy from HF or DFT 
calculation
					self.ENERGY = (float(outlines[i].split()[4]))

		if format == "Gaussian":
			uff = 0
			am1 = 0
			pm3 = 0
			scf = 0
			oniom = 0
			for i in range(0,len(outlines)):
				if outlines[i].find(" UFF") > -1: uff = i
				if outlines[i] .find("AM1") > -1: am1 = i
				if outlines[i].find("PM3") > -1: pm3 = i
				if outlines[i].find("ONIOM") > -1: oniom = i
				if outlines[i].find("SCF Done") > -1: scf = i

			calctype = [uff,am1,pm3,oniom,scf]
			for i in range(0,len(outlines)):
				if scf == max(calctype) and outlines[i].find("SCF Done") > -1 and outlines[i].find("Initial 
convergence to 1.0D-05 achieved")==-1: # Get energy from HF or DFT calculation
					self.ENERGY = (float(outlines[i].split()[4]))
				if oniom == max(calctype) and outlines[i].find("ONIOM: extrapolated energy") > -1: # Get 
energy from ONIOM calculation
					self.ENERGY = (float(outlines[i].split()[4]))
				if pm3 == max(calctype) or am1 == max(calctype) or uff == max(calctype):
					if outlines[i].find("Energy= ") > -1 and outlines[i].find("Predicted")==-1 and 
outlines[i].find("Thermal")==-1: # Get energy from Semi-
						self.ENERGY = (float(outlines[i].split()[1]))
				if outlines[i].find("Total free energy in solution") > -1:
					self.SOLVENERGY = (float(outlines[i+1].split()[7]))


	def getNMR(self, outlines, elem, format):
		self.NMR = []
		self.SHIFTS =[]
		if format == "Gaussian":
			for i in range(0,len(outlines)):
				if outlines[i].find("Isotropic") > -1: # Get Isotropic Shielding Tensor
					if outlines[i].split()[1] == elem:
						self.SHIFTS.append(int(outlines[i].split()[0]))
						self.NMR.append(float(outlines[i].split()[4]))

		if format == "Orca":
			for i in range(0,len(outlines)):
				if outlines[i].find("Nucleus ") > -1: # Get Isotropic Shielding Tensor
					if outlines[i+11].split()[0] == "Total":
						#print outlines[i]
						length = len(outlines[i].split()[1])-1
						string = (outlines[i].split()[1])
						#print string, length
						#print string[length]
						#print string[0:length]
						if string[length] == elem:
							self.SHIFTS.append(int(string[0:length])+1)
							#print outlines[i+11]
```

```
                                                                self.NMR.append(1000000*float(outlines[i+11].split()[5]))


                        if os.path.exists(file+".out"):outfile = open(file+".out","r")
                        else: outfile = open(file+".log","r")

                        outlines = outfile.readlines()

                        getFORMAT(self, outlines)
                        getJOBTYPE(self, outlines, self.FORMAT)
                        getCHARGE(self, outlines, self.FORMAT)
                        getENERGY(self, outlines, self.FORMAT)
                        getFREQS(self, outlines, self.FORMAT)
                        getCPU(self, outlines, self.FORMAT)
                        getATOMTYPES(self, outlines, self.FORMAT)
                        getNMR(self, outlines, elem, self.FORMAT)
                        if hasattr(self, "NATOMS"): getMULLIKEN(self, outlines, self.NATOMS, self.FORMAT)

                        #print "\nSuccessfully read geometry output", file


if __name__ == "__main__":

        #file(s)
        files = []

        # Takes arguments: (1) element of interest (2) input file(s)
        if len(sys.argv) > 3:
                for i in range(3,len(sys.argv)):
                        files.append(sys.argv[i].split(".")[0])
        else:
                print "\nWrong number of arguments used. Correct format: ccParse element temperature file(s)\n"
                sys.exit()

        elem = sys.argv[1]
        temp = float(sys.argv[2])
        energyarray = []
        shiftarray = []
        boltzfactor = []
        weightedshift = []
        globalmin = 0.0
        boltzsum = 0

        print "\no   NMR ANALYSIS PERFORMED FOR ELEMENT:",elem
        print "    STRUCTURE            ENERGY        ZPE        ENTHALPY        GIBBS-ENERGY        SOLVATION-ENERGY\n"
        for file in files:
                fileData = getoutData(file,elem)
                print "o   "+file,
                if hasattr(fileData, "ENERGY"): print fileData.ENERGY,
                else: print "  N/A           ",
                if hasattr(fileData, "ZPE"): print fileData.ZPE,
                else: print "  N/A           ",
                if hasattr(fileData, "ENTHALPY"): print fileData.ENTHALPY,
                else: print "  N/A           ",
                if hasattr(fileData, "GIBBS"): print fileData.GIBBS,
                else: print "  N/A           ",
                if hasattr(fileData, "SOLVENERGY"): print fileData.SOLVENERGY,
                else: print "  N/A",
                print "\n"
                if hasattr(fileData, "SOLVENERGY"): energyarray.append(fileData.SOLVENERGY)
else: energyarray.append(fileData.ENERGY)
                fileData.NMR =[]
                if len(fileData.NMR) == 0:
                        fileData = getoutData(file,elem)
                print "    SHIELDING TENSORS:",elem
                if hasattr(fileData, "NMR"):
                        for i in range(0,len(fileData.NMR)):
                                print "    "+elem+str(fileData.SHIFTS[i]), fileData.NMR[i]

                if hasattr(fileData, "NMR"): shiftarray.append(fileData.NMR)

        if len(energyarray) == len(shiftarray) and len(energyarray)!=0:
                print "\no   BOLTZMANN WEIGHTED ISOTROPIC SHIELDING TENSORS at "+str(temp)+"K"

                for i in range(0,len(energyarray)):
                        if energyarray[i]<globalmin: globalmin = energyarray[i]

                for i in range(0,len(energyarray)):
```

```
                boltzfactor.append(math.exp((globalmin-energyarray[i])*2625500/(8.314*temp)))
                boltzsum = boltzsum + math.exp((globalmin-energyarray[i])*2625500/(8.314*temp))

        for i in range(0,len(energyarray)):
                boltzfactor[i] = boltzfactor[i]/boltzsum

        print "    SHIELDING TENSORS:",elem
        for i in range(0,len(fileData.NMR)):
                weightedshift.append(0.0)

        for i in range(0,len(fileData.NMR)):
                for j in range(0,len(energyarray)):
                        weightedshift[i]=weightedshift[i]+boltzfactor[j]*shiftarray[j][i]


                print "    "+elem+str(fileData.SHIFTS[i]), weightedshift[i]

        print ""
```

compare_NMR_results.py: Compares computed shielding tensors against experimental chemical shifts; performs linear regression to obtain linear scaling constants; computes corrected mean absolute and root mean squared errors and DP4 probabilities; averages symmetry related nuclei; considers alternative assignments
*Example usage: python compare_NMR_results.py exptldata.txt computed_diastereomer*txt (where the computed data is formatted from getnmr.py)*

For example, for the following the computed shifts for a CH_3 (H29, H30, H31) and CH_2 (H26, H27) group are avaraged prior to comparison against experiment, while the assignments of H5/H6, H15/H16 amd H33/H44 are swapped to obtain the smallest error, which is useful for incomplete structural assignments.

```
$ cat NP_H1.txt

  H5 1.79
  H6 2.20
  …
  H38 5.58
  H41 2.83

  SWAP H5 H6
  SWAP H15 H16
  SWAP H33 H34
  AVERAGE H29 H30 H31
  AVERAGE H26 H27
```

```
#################################################################
#                    compare_NMR_results.py                     #
#                                                               #
#    To compare computed NMR (isotropic) shielding tensors against      #
#    an experimental spectrum                              ##
#################################################################

#Python Libraries
from scipy import stats
import subprocess, sys, os, commands, math, time, tarfile, random
from pstat import *
from stats import *
import scipy

## This can be used to assign a systematic shift to halogenated carbon, here we take an average error from our comparison of mPW1PW91/6-311G(d,p) shifts against chlorinated carbons in the NMRshift-DB
halogenshift=5.0
hal = []

## Either use linear scaling of each DFT structure individually (Default is scaling =1)
scaling = 1
C=0.0
M=1.0
## Or specify global scaling parameters, e.g. the shielding tensor for TMS (C) and 1 (M), or take these from the Chemical Shift Repository, http://cheshirenmr.info
#scaling = 0
```

```
# For 13C tensors at the mPW1PW91/6-311+G(d,p) level:
#C=180.127
#M=-0.964
# For 1H tensors at the mPW1PW91/6-311+G(d,p) level:
#C=30.287
#M=-0.945

## List of experimental Chemical Shifts Taken from text file
file = sys.argv[1]
exptfile = open(file,"r")
exptlines = exptfile.readlines()

## Read experimental chemical shifts from a raw text file
print "\no  Reading Experimental Data From", file,"\n"
exptshift=[]
atom=[]
swap=[]
average=[]
k=0; l=0

for i in range(0,len(exptlines)):
		if exptlines[i].find("C") > -1: nucleus = "13C"
		if exptlines[i].find("H") > -1: nucleus = "1H"
		if exptlines[i].find("C") > -1 or exptlines[i].find("H") > -1 and exptlines[i].find("AVERAGE") == -1 and exptlines[i].find("SWAP") == -1:
				exptshift.append(float(exptlines[i].split()[1]))
				atom.append((exptlines[i].split()[0]))
		if len(exptlines[i].split()) > 2:
				if exptlines[i].split()[2] == "hal": hal.append(exptlines[i].split()[0])
				if exptlines[i].split()[0] == "SWAP":
						swap.append([])
						for j in range(1,len(exptlines[i].split())): swap[k].append(exptlines[i].split()[j])
						k = k+1
				if exptlines[i].split()[0] == "AVERAGE":
						average.append([])
		    for j in range(1,len(exptlines[i].split())): average[l].append(exptlines[i].split()[j])
		    l = l+1


## The arrays to contain the computed shielding tensors/chemical shifts
dftshift=[]
dfttens=[]
dfterror=[]
lowval=[]

## Prepare to read DFT computed shielding tensors from any number of text files, each corresponding to a different chemical species
dftfile=[]
for i in range(2,len(sys.argv)):
		dftfile.append(sys.argv[i])


## Obtain the sheilding tensors from each text file
for j in range(0,len(dftfile)):
		print "   Comparison with DFT results from", dftfile[j]
		dfttens.append([])
		dftshift.append([])
		dfterror.append([])
		dft=open(dftfile[j], "r")
		dftlines = dft.readlines()
		for i in range(0,len(dftlines)):
				if dftlines[i].find("BOLTZMANN") > -1:
						startpoint=i
		for i in range(startpoint,len(dftlines)):
				if dftlines[i].find("C") > -1 or dftlines[i].find("H") > -1 and len(dftlines[i].split())<3:
						for k in range(0,len(atom)):
								if dftlines[i].find(" "+atom[k]+" ") > -1:
										#print dftlines[i], atom[k]
										tens = float(dftlines[i].split()[1])
										for carbon in hal:
												if atom[k] == carbon:
														tens = tens + halogenshift
														print "   -> applying halogen shift of", halogenshift, "to", atom[k]
										if atom[k] == lowatom:
												lowval.append(tens)
										#		print lowval
										#shift = C + M*tens
										dfttens[j].append(tens)
```

```
		print "   -> applying global scaling factors to shielding tensors of C=",C," M=",M
		for i in range(0,len(atom)):
			for av in average:
				if atom.index(av[0]) == i:
					avtens = 0.0
					for n in range(0,len(av)):
						avtens = avtens + dfttens[j][atom.index(av[n])]
					avtens = avtens/(n+1)
					#print "averaging", i, avtens
					for n in range(0,len(av)):
			   dfttens[j][atom.index(av[n])] = avtens
			#print i, dfttens[j][i]
			shift = C + M*dfttens[j][i]
			dftshift[j].append(shift)
			dfterror[j].append(exptshift[i]-shift)


## Metrics used to quantify DFT performance
rmserror=[]
meanerror=[]
varerror=[]
rsq=[]

setofexptdata = []
setofexptdata.append(exptshift)
## Generate expt data in which ambiguous assignments are swapped (pairwise)
#print exptshift
for pair in swap:
		for i in range(0,len(setofexptdata)):
			temp = []
			for j in range(0,len(atom)): temp.append(setofexptdata[i][j])
			#print i, temp
			a, b = atom.index(pair[0]), atom.index(pair[1])
			#print "Swapping ", a, "with", b
			temp[b], temp[a] = temp[a], temp[b]
			#print temp
			setofexptdata.append(temp)
			#print len(setofexptdata)


#print len(setofexptdata)
#for i in range(0,len(setofexptdata)):
		#print "Set", i
		#print setofexptdata[i]

## If individual scaling is to be performed, it is done here using a linear regression against the experimental data
## For each structure the errors and correlation is considered separately
for j in range(0,len(dftfile)):
		print "\no  Analysis of errors for", dftfile[j]
		bestsos = 999999999.9
		for k in range(0,len(setofexptdata)):
			gradient, intercept, r_value, p_value, std_err = stats.linregress(dfttens[j],setofexptdata[k])
			if scaling == 1:
				for i in range(0,len(atom)):
					dftshift[j][i] = intercept + gradient*dfttens[j][i]
					dfterror[j][i] = exptshift[i] - dftshift[j][i]
				#print "o  Performing linear scaling, using raw Gradient and intercept", gradient, intercept, r_value**2
			gradient, intercept, r_value, p_value, std_err = stats.linregress(dftshift[j],setofexptdata[k])
			sos = stats.sumdiffsquared(dftshift[j],setofexptdata[k])
			print "o  Sum of Squares", sos
			if sos < bestsos:
				bestsos = sos
				bestassignment = k

		print "Best assignment found ", bestassignment
		gradient, intercept, r_value, p_value, std_err = stats.linregress(dfttens[j],setofexptdata[bestassignment])
		if scaling == 1:
			for i in range(0,len(atom)):
				dftshift[j][i] = intercept + gradient*dfttens[j][i]
				dfterror[j][i] = setofexptdata[bestassignment][i] - dftshift[j][i]
			print "o  Performing linear scaling, using raw Gradient and intercept", gradient, intercept, r_value**2
		gradient, intercept, r_value, p_value, std_err = stats.linregress(dftshift[j],setofexptdata[bestassignment])
		sos = stats.sumdiffsquared(dftshift[j],setofexptdata[bestassignment])


		print "  " ,"No".rjust(3),"  ","DFT tensor".rjust(13),"  ","DFT shift".rjust(13),"  ","Expt".rjust(5), "  ", "Error".rjust(20)
		for i in range(0,len(atom)):
			print "  ",atom[i].rjust(3),"  ",str(dfttens[j][i]).rjust(13),"  ",str(dftshift[j][i]).rjust(13),"
",str(setofexptdata[bestassignment][i]).rjust(5),"  ",str(dfterror[j][i]).rjust(20)
```

```
		print "o  RMSE", math.pow(sos/len(atom),0.5)
		print "o  Gradient and intercept", gradient, intercept
		rsq.append(r_value**2)
		print "o  R-squared", r_value**2

## Across all of the structures, the errors are reported as a function of the atom
## This gives an idea of the variability of individual atoms
print "\no  Error for all structures as a function of position"
print "  ","No".rjust(3),"  ","Expt".rjust(5), "  ","Mean".rjust(16),"  ","RMS".rjust(16),"  ","Var".rjust(20)
for i in range(0,len(atom)):
	rmserror.append(0.0)
	meanerror.append(0.0)
	for j in range(0,len(dftfile)):
		rmserror[i]=rmserror[i]+dfterror[j][i]*dfterror[j][i]
		meanerror[i]=meanerror[i]+dfterror[j][i]
	rmserror[i]=rmserror[i]/(j+1)
	meanerror[i]=meanerror[i]/(j+1)
	varerror.append(rmserror[i]-meanerror[i]*meanerror[i])
	rmserror[i]=math.pow(rmserror[i],0.5)
	print "  ",atom[i].rjust(3),"  ", str(exptshift[i]).rjust(5),"  ", str(meanerror[i]).rjust(16),"  ", str(rmserror[i]).rjust(16),"  ", str(varerror[i]).rjust(20)

totalmeanerror = 0.0
totalrmserror = 0.0
count=0
for i in range(0,len(atom)):
	for j in range(0,len(dftfile)):
		totalrmserror=totalrmserror+dfterror[j][i]*dfterror[j][i]
		totalmeanerror=totalmeanerror+dfterror[j][i]
			count = count+1
totalmeanerror = totalmeanerror/count
totalrmserror = totalrmserror/count
totalvarerror = totalrmserror - totalmeanerror*totalmeanerror
totalrmserror = math.pow(totalrmserror,0.5)
print "  ","All atoms".rjust(8),"     ", str(totalmeanerror).rjust(16),"  ", str(totalrmserror).rjust(16),"  ", str(totalvarerror).rjust(20)


## Calculation of the root-mean-square (RMS) and mean absolute error (MAE) (these are "corrected" if scaling has been performed) for each structure
## Calculation of correlation coefficient (r2) and DP4 probability, using Student's T-distribution

## Underlying distributions necessary for DP4 analysis
# Original data from DP4 JACS paper; Smith, S. G.; Goodman, J. M. JACS 2010  132, 12946
normdist = scipy.stats.norm(0,1)
if nucleus == "13C":
		tdist = scipy.stats.t(11.382)
		sigma = 2.30586
if nucleus == "1H":
		tdist = scipy.stats.t(14.183)
		sigma = 0.18495


## Arrays used for metrics
prob = []
rmse =[]
mae = []
dp4prob = []
totprob = 0.0
totalprob = []
cumulativeprob = 0.0

## DP4 probabilities computed for each atom
for j in range(0,len(dftfile)):
		prob.append([])
		totprob=1.0
		for i in range(0,len(atom)):
				temp = math.fabs(dfterror[j][i]/sigma)
				prob[j].append(tdist.cdf(-temp))
				totprob=totprob*2*prob[j][i]
		totalprob.append(totprob)
		cumulativeprob = cumulativeprob + totprob

## RMSE, MAE computed for each structure
for j in range(0,len(dftfile)):
		rmse.append(0.0)
		mae.append(1.0)
		for i in range(0,len(atom)):
				rmse[j] = rmse[j] + math.pow(dfterror[j][i], 2.0)
				mae[j] = mae[j]+math.fabs(dfterror[j][i])
```

```
            rmse[j] = math.pow(rmse[j]/len(atom), 0.5)
            mae[j] = mae[j]/len(atom)

## Comparison of indiviudal MAE against the average from the structures considered
totalmae = 0.0
totalsquaredmae = 0.0
for j in range(0,len(dftfile)):
            totalmae = totalmae + mae[j]
            totalsquaredmae = totalsquaredmae + mae[j]*mae[j]
meanmae = totalmae/len(dftfile)
varmae = totalsquaredmae/len(dftfile) - meanmae*meanmae
sdmae = math.pow(varmae,0.5)


## Normalize DP4 probablitiies, and output all metrics
print "\n  " ,"".rjust(30),"  ","(C)RMSE".rjust(12), "  ","(C)MAE".rjust(12),"  ","R-Squared".rjust(12),"  ","DP4".rjust(6), "  ", "SDs above average"
for j in range(0,len(dftfile)):
            dp4prob.append(totalprob[j]/cumulativeprob)
            if sdmae > 0.0: print "o  Metrics for", dftfile[j], rmse[j], mae[j], rsq[j], dp4prob[j], (mae[j]-meanmae)/sdmae

print "\no  Average and S.D. for MAEs:", meanmae, sdmae, "\n"
```

2.8 Computational References

1. Sheldrake, H. M.; Jamieson, C.; Burton, J. W. *Angew. Chem. Int. Ed.* **2006**, *45*, 7199-7202; Sheldrake, H. M.; Jamieson, C.; Pascu, S. I.; Burton, J. W. *Org. Biomol. Chem.* **2009**, *7*, 238-252.
2. Abdel-Mageed, W. M.; Ebel, R.; Valeriote, F. A.; Jaspars, M. *Tetrahedron* **2010**, *66*, 2855-2862.
3. (a) mPW1PW91: Adomo, C.; Barone, V. *J. Chem. Phys.* **1998**, *108*, 664; (b) wB97XD: Chai, J.-D. Head-Gordon, M. *Phys. Chem. Chem. Phys*. **2008**, *10*, 6615.
4. Barone, V.; Cossi, M. *J. Phys. Chem. A,* **1998**, *102*, 1995; Cossi, M.; Rega, N.; Scalmani, G.; Barone, V. *J. Comp. Chem.* **2003**, *24*, 669.
5. Lodewyk, M. W.; Tantillo, D. J. J. Nat. Prod. **2011**, 74, 1339; Lodewyk, M. W.; Soldi, C.; Jones, P. B.; Olmstead, M. M.; Rita, J.; Shaw, J. T.; Tantillo, D. J. *J. Am. Chem. Soc.* **2012**, *134*, 18550; Quasdorf, K. W.; Huters, A. D.; Lodewyk, M. W.; Tantillo, D. J.; Garg, N. K. *J. Am. Chem. Soc.* **2012**, *134*, 1396.
6. For recent examples of DFT calculations of NMR chemical shifts in natural product structure determination see : (a) Aplidinones A-C - Aiello, A.; Fattorusso, E.; Luciano, P.; Mangoni, A.; Menna, M. *Eur. J. Org. Chem.* **2005**, 5024–5030. (b) Jungianol - da Silva, G. V. J.; Neto, Á. C. *Tetrahedron* **2005**, *61*, 7763–7767. (c) Hexacyclinol - Rychnovsky, S. D. *Org. Lett.* **2006**, *8*, 2895–2898; Saielli, G.; Bagno, A. *Org. Lett.* **2009**, *11*, 1409–1412. (d) Maitotoxin - Nicolaou, K. C.; Frederick, M. O. *Angew. Chem., Int. Ed.* **2007**, *46*, 5278–5282. (d) Jungianol - da Silva, G. V. J.; Neto, Á. C. *Tetrahedron* **2005**, *61*, 7763–7767. (e) Gloriosaols - Bassarello, C.; Bifulco, G.; Montoro, P.; Skhirtladze, A.; Kemertelidze, E.; Pizza, C.; Piacente, S. *Tetrahedron* **2007**, *63*, 148–154. (f) Kadlongilatones D and F - Pu, J.-X.; Huang, S.-X.; Ren, J.; Xiao, W.-L.; Li, L.-M.; Li, R.-T.; Li, L.-B.; Liao, T.-G.; Lou, L.-G.; Zhu, H.-J.; Sun, H.-D. *J. Nat. Prod.* **2007**, *70*, 1707–1711. (g) Artarborol - Fattorusso, C.; Stendardo, E.; Appendino, G.; Fattorusso, E.; Luciano, P.; Romano, A.; Taglialatela-Scafati, O. *Org. Lett.* **2007**, *9*, 2377–2380. (h) Obtusallenes - Braddock, D. C.; Rzepa, H. S. *J. Nat. Prod.* **2008**, *71*, 728–730. (i) Spiroleucettadine - White, K. N.; Amagata, T.; Oliver, A. G.; Tenney, K.; Wenzel, P. J.; Crews, P. *J. Org. Chem.* **2008**, *73*, 8719–8722. (j) Samoquasine A - Timmons, C.; Wipf, P. *J. Org. Chem.* **2008**, *73*, 9168–9170. (k) Mururin C - Hu, G.; Liu, K.; Williams, L. J. *Org. Lett.* **2008**, *10*, 5493–5496. (l) Hassananes - Yang, J.; Huang, S.-X.; Zhao, Q.-S. *J. Phys. Chem. A* **2008**, *112*, 12132–12139. (m) Ketopelenolides C and D - Fattorusso, E.; Luciano, P.; Romano, A.; Taglialatela-Scafati, O.; Appendino, G.; Borriello, M.; Fattorusso, C. *J. Nat. Prod.* **2008**, *71*, 1988–1992. (n) 6β-Hydroxyhyoscyamine - Muñoz, M. A.; Joseph-Nathan, P. *Magn. Reson. Chem.* **2009**, *47*, 578– 584. (o) Dolichodial - Wang, B.; Dossey, A. T.; Walse, S. S.; Edison, A. S.; Merz, K. M., Jr. *J. Nat. Prod.* **2009**, *72*, 709–713. (p) Hypuriticin - Mendoza-Espinoza, J. A.; López-Vallejo, F.; Fragoso-Serrano, M.; Pereda-Miranda, R.; Cerda-García-Rojas, C.M. *J. Nat. Prod.* **2009**, *72*, 700–708. (q) Santalol derivatices - Stappen, I.; Buchbauer, G.; Robien, W.; Wolchann, P. *Magn. Reson. Chem.* **2009**, *47*, 720–726. (r) Aplysqualenols - Vera, B.; Rodríguez, A. D.; Avilés, E.; Ishikawa, Y. *Eur. J. Org. Chem.* **2009**, 5327-5336. (s) Fusapyrones - Honma, M.; Kudo, S.; Takada, N.; Tanaka, K.; Miura, T.; Hashimoto, M. *Bioorg. Med. Chem. Lett.* **2010**, *20*, 709–712. (t) 9-*epi*-Presilphiperfolan-1-ol - Pinto, S. C.; Leitão, G. G.; Bizzo, H. R.; Martinez, N.; Dellacassa, E.; dos Santos, F. M., Jr.; Costa, F. L. P.; de Amorim, M. B.; Leitão, S. G. *Tetrahedron Lett.* **2009**, *50*, 4785–4787; Joseph-Nathan, P.; Leitão, S. G.; Pinto, S. C.; Leitão, G. G.; Bizzo, H. R.; Costa, F. L. P.; de Amorim, M. B.; Martinez, N.; Dellacassa, E.; Hernández-Barragán, A.; Pérez-Hernández, N. *Tetrahedron Lett.* **2010**, *51*, 1963–1965. (u) For a retrospective look at how DFT calculations would have been useful in determiming the full stereostructure of vannusal B see: Saielli, G.; Nicolaou, K. C.; Ortiz, A.; Zhang, H. J.; Bagno, A. *J. Am. Chem. Soc.* **2011**, *133*, 6072-6077. (v) Chloroscabrolides - Fattorusso, E.; Luciano, P.; Putra, M. Y.; Taglialatela-Scafati, O.; Ianaro, A.; Panza, E.; Bavestrello, G.; Cerrano, C. *Tetrahedron* **2011**, *67*, 7983-7988. (w) Perthamides - Festa, C.; De Marino, S.; Sepe, V.; D'Auria, M. V.; Bifulco, G.; Andrés, R.; Terencio, M. C.; Payá, M.; Debitus, C.; Zampella, A. *Tetrahedron* **2011**, *67*, 7780-7786. (x) Solomonamindes - Festa, C.; De Marino, S.; Sepe, V.; D'Auria, M. V.; Bifulco, G.; Débitus, C.; Bucci, M.; Vellecco, V.; Zampella, A. *Org. Lett.* **2011**, *13*, 1532-1535. (y) Malaitasterol A - De Marino, S.; Sepe, V.; D'Auria, M. V.; Bifulco, G.; Renga, B.; Petek, S.; Fiorucci, S.; Zampella, A. *Org. Biomol. Chem.* **2011**, *9*, 4856-4862. (z) Iosmalyngamide K - Han, B.; Reinscheid, U. M.; Gerwick, W. H.; Gross, H. *J. Mol. Struct.* **2011**, *989*, 109-113.

7. For recent reviews see: Bifulco, G.; Dambruoso, P.; Gomez-Paloma, L.; Riccio, R. *Chem. Rev.* **2007**, *107*, 3744; Di Micco, S.; Chini, M. G.; Riccio, R.; Bifulco, G. *Eur. J. Org. Chem.* **2010**, 1411; Lodewyk, M. W.; Siebert, M. R.; Tantillo, D. J. *Chem. Rev.* **2012**, *112*, 1839. Tantillo and co-workers' Chemical Shift Repository contains scaling factors obtained for various functionals and basis sets obtained for test sets of organic molecules: http://cheshirenmr.info
8. Smith, S. G.; Goodman, J. M. *J. Am. Chem. Soc.* **2010**, *132*, 12946-12959.
9. Chang, G.; Guida, W. C.; Still, W. C. *J. Am. Chem. Soc.* **1989**, *111*, 4379-4386.
10. Halgren, T. A. *J. Comput. Chem.* **1996**, *17*, 490–519, 520-552, 553-586, 587-615, 616-641 and *J. Comput Chem*. **1999**, 20, 730-748.
11. Mohamadi, F.; Richards, N. G. J.; Guida, W. C.; Liskamp, R.; Lipton, M.; Caufield, C.; Chang, G.; Hendrickson, T.; Still, W. C. *J. Comput. Chem.* **1990**, *11*, 440.
12. D.A. Case, T.A. Darden, T.E. Cheatham, III, C.L. Simmerling, J. Wang, R.E. Duke, R. Luo, R.C. Walker, W. Zhang, K.M. Merz, B. Roberts, S. Hayik, A. Roitberg, G. Seabra, J. Swails, A.W. Goetz, I. Kolossváry, K.F. Wong, F. Paesani, J. Vanicek, R.M. Wolf, J. Liu, X. Wu, S.R. Brozell, T. Steinbrecher, H. Gohlke, Q. Cai, X. Ye, J. Wang, M.-J. Hsieh, G. Cui, D.R. Roe, D.H. Mathews, M.G. Seetin, R. Salomon-Ferrer, C. Sagui, V. Babin, T. Luchko, S. Gusarov, A. Kovalenko, and P.A. Kollman (2012), *AMBER 12*, University of California, San Francisco.
13. MOPAC2012, Stewart, J. J. P. Stewart Computational Chemistry, Colorado Springs, CO, USA, http://OpenMOPAC.net
14. Paton, R. S.; Goodman, J. M. *J. Chem. Inf. Model.* **2009,** *49,* 944.
15. Gaussian 09, Revision A.1, M. J. Frisch, G. W. Trucks, H. B. Schlegel, G. E. Scuseria, M. A. Robb, J. R. Cheeseman, G. Scalmani, V. Barone, B. Mennucci, G. A. Petersson, H. Nakatsuji, M. Caricato, X. Li, H. P. Hratchian, A. F. Izmaylov, J. Bloino, G. Zheng, J. L. Sonnenberg, M. Hada, M. Ehara, K. Toyota, R. Fukuda, J. Hasegawa, M. Ishida, T. Nakajima, Y. Honda, O. Kitao, H. Nakai, T. Vreven, J. A. Montgomery, Jr., J. E. Peralta, F. Ogliaro, M. Bearpark, J. J. Heyd, E. Brothers, K. N. Kudin, V. N. Staroverov, R. Kobayashi, J. Normand, K. Raghavachari, A. Rendell, J. C. Burant, S. S. Iyengar, J. Tomasi, M. Cossi, N. Rega, J. M. Millam, M. Klene, J. E. Knox, J. B. Cross, V. Bakken, C. Adamo, J. Jaramillo, R. Gomperts, R. E. Stratmann, O. Yazyev, A. J. Austin, R. Cammi, C. Pomelli, J. W. Ochterski, R. L. Martin, K. Morokuma, V. G. Zakrzewski, G. A. Voth, P. Salvador, J. J. Dannenberg, S. Dapprich, A. D. Daniels, Ö. Farkas, J. B. Foresman, J. V. Ortiz, J. Cioslowski, and D. J. Fox, Gaussian, Inc., Wallingford CT, 2009.
